# Supplementary material for: Powerful Potential of Polyfluoroalkyl-Containing 4-Arylhydrazinylidenepyrazol-3-ones for Pharmaceuticals
Source: Molecules. 2022 Dec 21;28(1):59. doi: 10.3390/molecules28010059 (PMC9821843; doi:10.3390/molecules28010059)

Powerful Potential of Polyfluoroalkyl-containing 4-Arylhydrazinylidenepyrazol-3-ones for Pharmaceuticals

Yanina V. Burgart [1], Natalia A. Elkina [1], Evgeny V. Shchegolkov [1], Olga P. Krasnykh [2], Galina F. Makhaeva [3], Galina A. Triandafilova [2], Sergey Yu. Solodnikov [2], Natalia P. Boltneva [3], Elena V. Rudakova [3], Nadezhda V. Kovaleva [3], Olga G. Serebryakova [3], Mariya V. Ulitko [4], Sophia S. Borisevich [1], Natalia A. Gerasimova [5], Natalia P. Evstigneeva [5], Sergey A. Kozlov [6], Yuliya V. Korolkova [6], Artem S. Minin [4,7], Anna V. Belousova [8], Evgenii S. Mozhaitsev [9], Artem M. Klabukov [10] and Victor I. Saloutin [1,*]

[1] Postovsky Institute of Organic Synthesis of the Ural Branch of the Russian Academy of Science (IOS UB RAS), S. Kovalevskoi St., 22, Ekaterinburg 620108, Russia; ya.burgart@yandex.ru (Y.V.B.); natali741258@mail.ru (N.A.E.); e.schegolkov@yandex.ru (E.V.S.); monrel@mail.ru (S.S.B.)

[2] Scientific and Educational Center for Applied Chemical-Biological Research, Perm National Research Poly-technic University, Komsomolsky Av., 29, Perm 614990, Russia; ol.krasnykh@gmail.com (O.P.K.); lindick@yandex.ru (G.A.T.); s.u.solodnikov@rambler.ru (S.Y.S.)

[3] Institute of Physiologically Active Compounds at Federal Research Center of Problems of Chemical Physics and Medicinal Chemistry, Russian Academy of Sciences (IPAC RAS), Severny proezd 1, Chernogolovka 142432, Russia; gmakh@ipac.ac.ru (G.F.M.); kovalevanv@ipac.ac.ru (N.V.K.); boltneva@ipac.ac.ru (N.P.B.); rudakova@ipac.ac.ru (E.V.R.); sog@ipac.ac.ru (O.G.S.)

[4] Institute of Natural Sciences and Mathematics of the Ural Federal University Named after the First President of Russia B. N. Yeltsin, Lenina Av., 51, Ekaterinburg 620083, Russia; mv.ulitko@urfu.ru (M.V.U.)

[5] Ural Research Institute for Dermatology, Venereology and Immunopathology, Shcherbakova St., 8, Ekaterinburg 620076, Russia; ngerasimova2010@gmail.com (N.A.G.); evstigneeva-np@yandex.ru (N.P.E.)

[6] Shemyakin-Ovchinnikov Institute of Bioorganic Chemistry RAS, Miklukho-Maklaya St., 16/10, Moscow 117997, Russia; serg@ibch.ru (S.A.K.); july@mx.ibch.ru (Y.V.K.)

[7] M.N. Mikheev Institute of Metal Physics of the Ural Branch of the Russian Academy of Sciences, S. Kovalevskoi 18, Ekaterinburg 620108, Russia; calamatica@gmail.com (A.S.M.)

[8] Institute of Immunology and Physiology of the Ural Branch of the Russian Academy of Sciences, Pervomayskaya 106, Ekaterinburg 620108, Russia (IIP UB RAS); a1b2v3@mail.ru (A.V.B.)

[9] N.N. Vorozhtsov Novosibirsk Institute of Organic Chemistry of Siberian Branch of Russian Academy of Sciences, 9 Lavrentiev Avenue, Novosibirsk, 630090, Russia; mozhaitsev@gmail.com (E.S.M.)

[10] Smorodintsev Research Institute of Influenza of the Ministry of Health of the Russian Federation, 15/17 prof. Popov street, Saint-Petersburg, 197376, Russia; temaklab@gmail.com (A.M.K.)

* Correspondence: saloutin@ios.uran.ru

Table of contents

Table S1. *In silico* ADME predictions of pyrazolones **5-9** 5

Table S2. Antifungal activity of 4-arylhydrazinylidenepyrazolones **5-9** 7

Figure S1. Z, E isomers of compound **5a** 8

Figure S2. Images of Vero cells stained with compounds **5f** (**A**) and **9a** (**B**) exciting by lasers with different wavelengths. 9

Figure S3. ^{1}H NMR spectrum of compound **5a** 10

Figure S4. ^{13}C NMR spectrum of compound **5a** 11

Figure S5. ^{19}F NMR spectrum of compound **5a** 12

Figure S6. ^{1}H NMR spectrum of compound **5b** 13

Figure S7. ^{19}F NMR spectrum of compound **5b** 14

Figure S8. ^{1}H NMR spectrum of compound **5c** 15
Figure S9. ^{13}C NMR spectrum of compound **5c** 16
Figure S10. ^{19}F NMR spectrum of compound **5c** 17
Figure S11. ^{1}H NMR spectrum of compound **5d** 18
Figure S12. ^{19}F NMR spectrum of compound **5d** 19
Figure S13. ^{1}H NMR spectrum of compound **5e** 20
Figure S14. ^{19}F NMR spectrum of compound **5e** 21
Figure S15. ^{1}H NMR spectrum of compound **5f** 22
Figure S16. ^{13}C NMR spectrum of compound **5f** 23
Figure S17. ^{19}F NMR spectrum of compound **5f** 24
Figure S18. ^{1}H NMR spectrum of compound **5g** 25
Figure S19. ^{13}C NMR spectrum of compound **5g** 26
Figure S20. ^{19}F NMR spectrum of compound **5g** 27
Figure S21. ^{1}H NMR spectrum of compound **5h** 28
Figure S22. ^{13}C NMR spectrum of compound **5h** 29
Figure S23. ^{19}F NMR spectrum of compound **5h** 30
Figure S24. ^{1}H NMR spectrum of compound **5i** 31
Figure S25. ^{13}C NMR spectrum of compound **5i** 32
Figure S26. ^{19}F NMR spectrum of compound **5i** 33
Figure S27. ^{1}H NMR spectrum of compound **5j** 34
Figure S28. ^{13}C NMR spectrum of compound **5j** 35
Figure S29. ^{19}F NMR spectrum of compound **5j** 36
Figure S30. ^{1}H NMR spectrum of compound **5k** 37
Figure S31. ^{13}C NMR spectrum of compound **5k** 38
Figure S32. ^{19}F NMR spectrum of compound **5k** 39
Figure S33. ^{1}H NMR spectrum of compound **5l** 40
Figure S34. ^{13}C NMR spectrum of compound **5l** 41
Figure S35. ^{19}F NMR spectrum of compound **5l** 42
Figure S36. ^{1}H NMR spectrum of compound **5m** 43
Figure S37. ^{13}C NMR spectrum of compound **5m** 44
Figure S38. ^{19}F NMR spectrum of compound **5m** 45
Figure S39. ^{1}H NMR spectrum of compound **5n** 46
Figure S40. ^{13}C NMR spectrum of compound **5n** 47
Figure S41. ^{19}F NMR spectrum of compound **5n** 48
Figure S42. ^{1}H NMR spectrum of compound **5o** 49
Figure S43. ^{13}C NMR spectrum of compound **5o** 50
Figure S44. ^{19}F NMR spectrum of compound **5o** 51

Figure S45. ^{1}H NMR spectrum of compound **5p** 52
Figure S46. ^{13}C NMR spectrum of compound **5p** 53
Figure S47. ^{19}F NMR spectrum of compound **5p** 54
Figure S48. ^{1}H NMR spectrum of compound **5q** 55
Figure S49. ^{19}F NMR spectrum of compound **5q** 56
Figure S50. ^{1}H NMR spectrum of compound **5r** 57
Figure S51. ^{13}C NMR spectrum of compound **5r** 58
Figure S52. ^{19}F NMR spectrum of compound **5r** 59
Figure S53. ^{1}H NMR spectrum of compound **6a** 60
Figure S54. ^{13}C NMR spectrum of compound **6a** 61
Figure S55. ^{19}F NMR spectrum of compound **6a** 62
Figure S56. ^{1}H NMR spectrum of compound **6b** 63
Figure S57. ^{13}C NMR spectrum of compound **6b** 64
Figure S58. ^{19}F NMR spectrum of compound **6b** 65
Figure S59. ^{1}H NMR spectrum of compound **6c** 66
Figure S60. ^{13}C NMR spectrum of compound **6c** 67
Figure S61. ^{19}F NMR spectrum of compound **6c** 68
Figure S62. ^{1}H NMR spectrum of compound **6d** 69
Figure S63. ^{13}C NMR spectrum of compound **6d** 70
Figure S64. ^{19}F NMR spectrum of compound **6d** 71
Figure S65. ^{1}H NMR spectrum of compound **6e** 72
Figure S66. ^{13}C NMR spectrum of compound **6e** 73
Figure S67. ^{19}F NMR spectrum of compound **6e** 74
Figure S68. ^{1}H NMR spectrum of compound **6f** 75
Figure S69. ^{13}C NMR spectrum of compound **6f** 76
Figure S70. ^{19}F NMR spectrum of compound **6f** 77
Figure S71. ^{1}H NMR spectrum of compound **6g** 78
Figure S72. ^{13}C NMR spectrum of compound **6g** 79
Figure S73. ^{19}F NMR spectrum of compound **6g** 80
Figure S74. ^{1}H NMR spectrum of compound **6h** 81
Figure S75. ^{13}C NMR spectrum of compound **6h** 82
Figure S76. ^{19}F NMR spectrum of compound **6h** 83
Figure S77. ^{1}H NMR spectrum of compound **6i** 84
Figure S78. ^{19}F NMR spectrum of compound **6i** 85
Figure S79. ^{1}H NMR spectrum of compound **7a** 86
Figure S80. ^{13}C NMR spectrum of compound **7a** 87
Figure S81. ^{19}F NMR spectrum of compound **7a** 88

Figure S82. ^{1}H NMR spectrum of compound **7b** 89
Figure S83. ^{13}C NMR spectrum of compound **7b** 90
Figure S84. ^{19}F NMR spectrum of compound **7b** 91
Figure S85. ^{1}H NMR spectrum of compound **8a** 92
Figure S86. ^{13}C NMR spectrum of compound **8a** 93
Figure S87. ^{19}F NMR spectrum of compound **8a** 94
Figure S88. ^{1}H NMR spectrum of compound **8b** 95
Figure S89. ^{13}C NMR spectrum of compound **8b** 96
Figure S90. ^{19}F NMR spectrum of compound **8b** 97
Figure S91. ^{1}H NMR spectrum of compound **9a** 98
Figure S92. ^{13}C NMR spectrum of compound **9a** 99
Figure S93. ^{1}H NMR spectrum of compound **9b** 100
Figure S94. ^{13}C NMR spectrum of compound **9b** 101

Table S1. *In silico* ADME predictions of pyrazolones **5-9**

ID of ligand	dipole	IP (eV)	EA (eV)	SASA	FOSA	FISA	PISA	WPSA	volume	glob	QPpolrz	QPlogPC16	QPlogPoct
min	1.0	7.9	0.9	300	0	7	0	0	500	0.75	13.0	4.0	8.0
max	12.5	10.5	1.7	1000	750	330	450	175	2000	0.95	70.0	18.0	35.0
**5a**	2.2	8.9	1.6	456	1	128	212	115	742	0.87	23.6	6.9	12.0
**5b**	2.6	8.7	1.5	621	89	64	354	114	1054	0.81	37.5	10.2	15.2
**5c**	2.5	9.0	1.7	485	1	128	164	192	795	0.86	25.3	7.6	12.9
**5d**	2.5	8.8	1.6	523	179	73	156	115	865	0.84	28.0	7.0	11.7
**5e**	2.6	8.8	1.5	589	1	64	410	115	994	0.82	35.6	10.0	14.6
**5f**	2.4	8.8	1.6	488	89	128	157	115	802	0.85	25.5	7.0	12.6
**5g**	1.3	8.5	1.5	627	94	64	354	115	1070	0.81	37.5	10.3	15.5
**5h**	3.8	8.9	1.6	598	1	64	372	161	1010	0.81	35.9	9.5	15.0
**5i**	0.9	8.9	1.6	604	1	57	373	173	1031	0.82	36.7	10.4	15.2
**5j**	2.7	8.9	1.6	609	1	54	348	207	1057	0.82	37.5	10.8	15.7
**5k**	3.5	8.9	1.6	618	1	64	361	192	1047	0.81	37.3	10.7	15.6
**5l**	2.1	9.1	1.8	714	142	118	340	114	1218	0.77	42.6	12.1	18.1
**5m**	7.7	9.3	1.8	675	84	142	333	115	1161	0.79	40.9	11.6	19.5
**5n**	9.0	9.4	1.9	660	1	205	338	116	1130	0.79	39.1	12.3	22.6
**5o**	8.5	9.4	1.9	658	1	201	340	117	1124	0.79	38.8	12.0	20.6
**5p**	6.4	8.9	1.8	688	89	202	281	116	1184	0.79	40.7	12.4	22.5
**5r**	5.2	8.9	1.7	702	172	140	275	115	1214	0.78	42.5	11.7	19.5
**5s**	2.9	8.7	1.5	653	177	64	298	115	1114	0.80	39.3	10.4	15.7
**6a**	2.7	8.8	1.6	521	89	122	149	162	876	0.85	27.8	7.4	13.5
**6b**	2.7	8.8	1.6	649	89	56	343	161	1121	0.80	39.4	10.4	16.0
**6c**	1.2	8.9	1.7	631	0	50	361	219	1097	0.82	38.6	10.7	16.0
**6d**	4.4	9.0	1.7	641	0	56	351	233	1105	0.81	38.9	10.8	16.3
**6e**	3.6	9.3	1.5	650	0	45	336	269	1139	0.81	40.1	11.2	16.8
**6f**	1.8	8.9	1.8	655	0	50	314	291	1142	0.81	40.0	11.3	16.7
**6g**	2.1	9.1	1.8	746	141	112	332	161	1292	0.77	44.9	12.5	19.0
**6h**	7.1	9.4	1.9	703	84	135	323	161	1229	0.79	42.9	11.9	20.1
**7a**	2.8	8.8	1.7	551	89	118	149	195	937	0.84	29.6	7.7	14.3
**7b**	2.9	8.8	1.6	687	88	56	343	200	1198	0.79	41.9	10.9	17.0
**8a**	2.9	8.8	1.7	597	88	121	149	239	1031	0.83	32.7	8.3	15.5

**8b**	3.4	8.7	1.6	738	88	53	352	245	1290	0.78	45.0	11.6	18.3
**9a**	1.7	8.6	1.2	470	174	134	161	0	763	0.86	24.0	7.5	11.5
**9b**	1.3	8.5	1.1	602	174	70	358	0	1015	0.81	36.0	10.6	14.1

dipole – computed dipole moment of the molecule;
IP (ev) – PM3 calculated ionization potential (negative of HOMO energy);
EA (eV) – PM3 calculated electron affinity (negative of LUMO energy);
SASA – total solvent accessible surface area (SASA) in square angstroms using a probe with a 1.4 Å radius;
FOSA – hydrophobic component of the SASA (saturated carbon and attached hydrogen);
FISA – hydrophilic component of the SASA (SASA on N. O. H on heteroatoms. carbonyl C);
PISA – π (carbon and attached hydrogen) component of the SASA;
WPSA – weakly polar component of the SASA (halogens. P. and S);
volume – total solvent-accessible volume in cubic angstroms using a probe with a 1.4 Å radius;
glob – globularity descriptor.$(4\pi r^2)/(SASA)$. where r is the radius of a sphere with a volume equal to the molecular volume. Globularity is 1.0 for a spherical molecule;
QPpolrz – predicted polarizability in cubic angstroms;
QPlogPC16 – predicted hexadecane/gas partition coefficient;
QPlogPoct – predicted octanol/gas partition coefficient.

Table S2. Antifungal activity of 4-arylhydrazinylidenepyrazolones **5-9**

**No**	**Compounds** 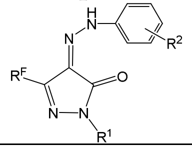			**MIC (μg/ml) for fungi strains inhibition**			
	**R^F**	**R^1**	**R^2**	***T. rubrum***	***E. floccosum***	***M. canis***	***C. albicans***
**5b**	CF_3	H	Me-4	50	>200	100	200
**5c**	CF_3	H	Br-4	**12.5**	**12.5**	**25**	>200
**5d**	CF_3	Me	Me-4	200	200	200	>200
**5e**	CF_3	Ph	H	200	200	200	>200
**5f**	CF_3	Ph	Me-4	>200	200	200	200
**5h**	CF_3	Ph	F-4	>200	>200	>200	>200
**5i**	CF_3	Ph	Cl-2	>200	>200	>200	>200
**5j**	CF_3	Ph	Cl_2-2,6	>200	>200	>200	>200
**5k**	CF_3	Ph	Br-4	>200	>200	>200	>200
**5m**	CF_3	Ph	SO_2Me-4	100	>200	>200	>200
**5p**	CF_3	$C_6H_4SO_2NH_2$-4	Me-4	>200	>200	>200	>200
**5r**	CF_3	$C_6H_4SO_2Me$-4	Me-4	>200	>200	>200	>200
**6c**	C_2F_5	Ph	Cl-2	200	>200	>200	>200
**6d**	C_2F_5	Ph	Cl-3	>200	200	>200	>200
**6e**	C_2F_5	Ph	Cl_2-2,6	>200	200	>200	>200
**6f**	C_2F_5	Ph	Cl_2-2,4	>200	200	>200	>200
**6g**	C_2F_5	Ph	CO_2Et-4	>200	>200	>200	>200
**6h**	C_2F_5	Ph	SO_2Me-4	>200	>200	>200	>200
**7a**	C_3F_7	H	Me-4	>200	200	>200	>200
**8a**	C_4F_9	H	Me-4	>200	>200	>200	>200
**9b**	Me	Ph	Me-4	>200	>200	>200	>200
Fluconazole				3.12	1.56	3.12	1.56

Figure S1. Z, E isomers of compound **5a**.

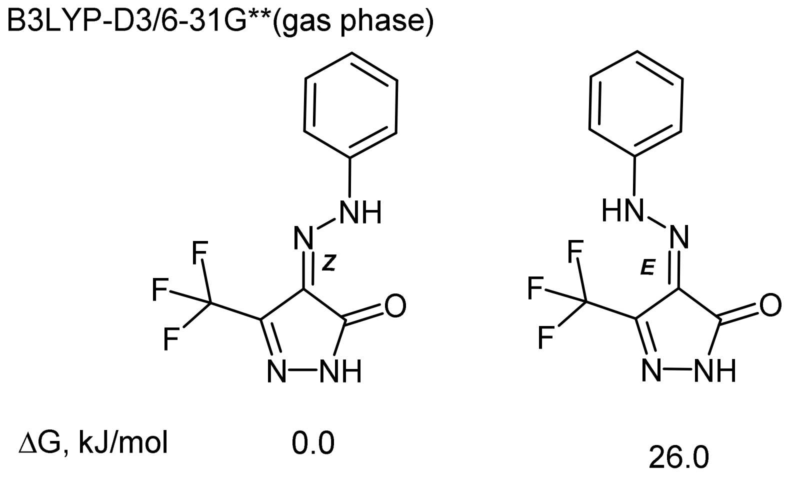

Figure S2. Images of Vero cells stained with compounds **5f** (**A**) and **9a** (**B**) exciting by lasers with different wavelengths.

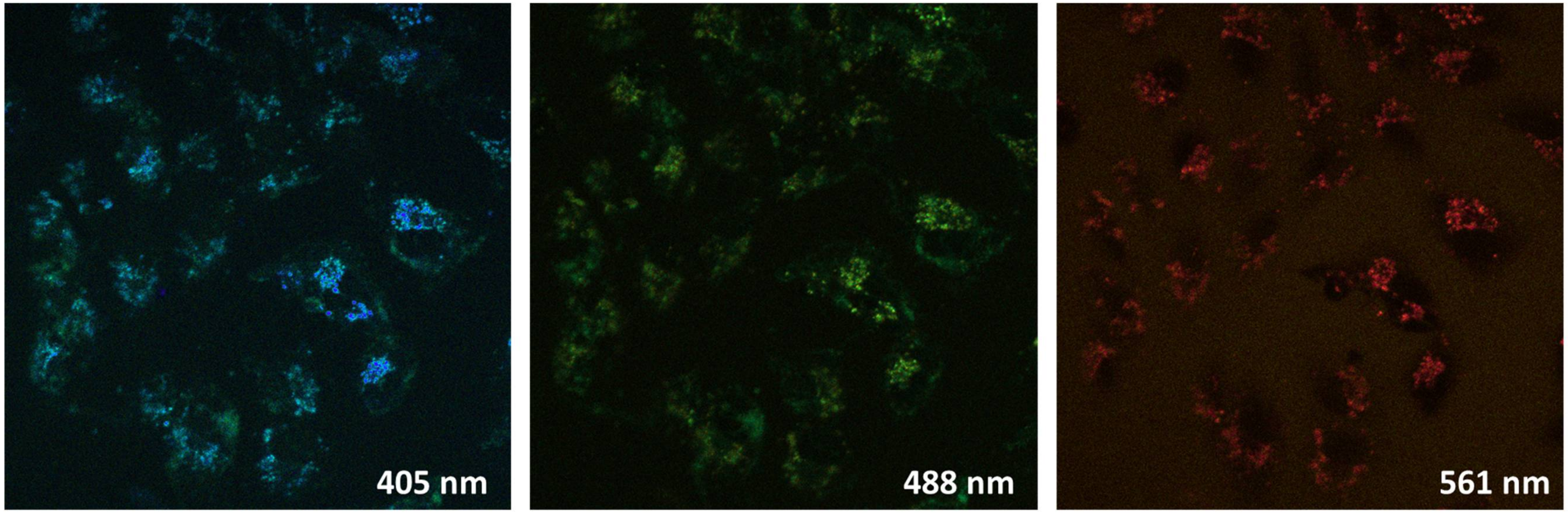

(**A**)

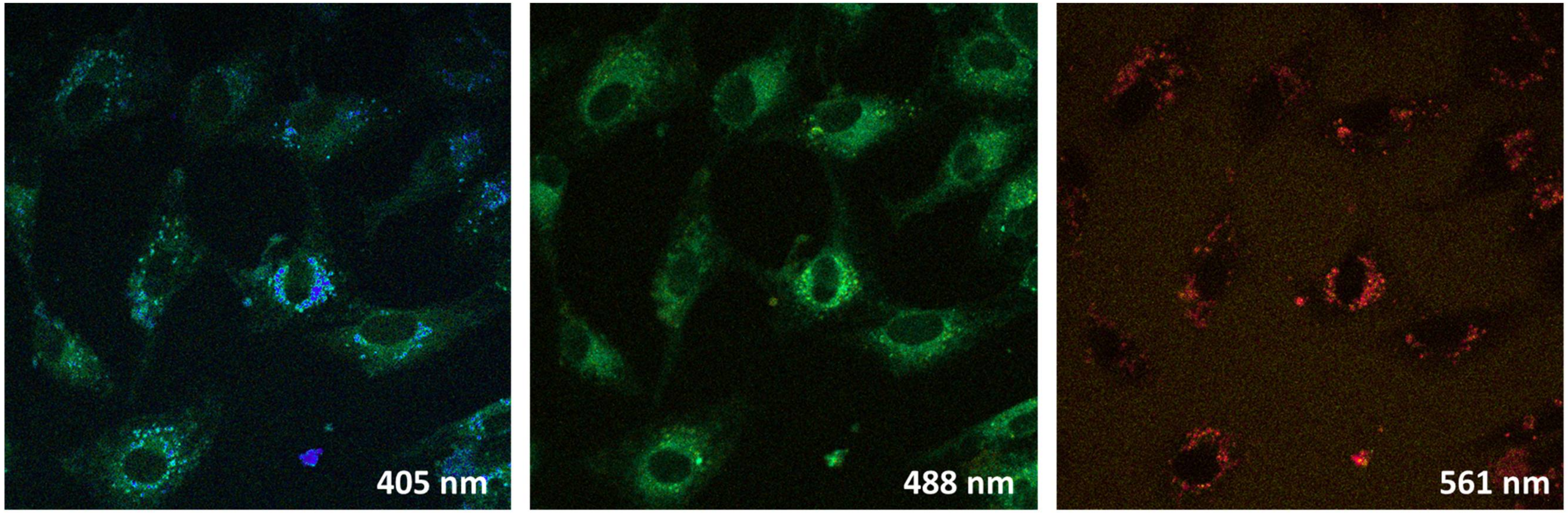

(**B**)

Figure S3. ^{1}H NMR spectrum of compound **5a**

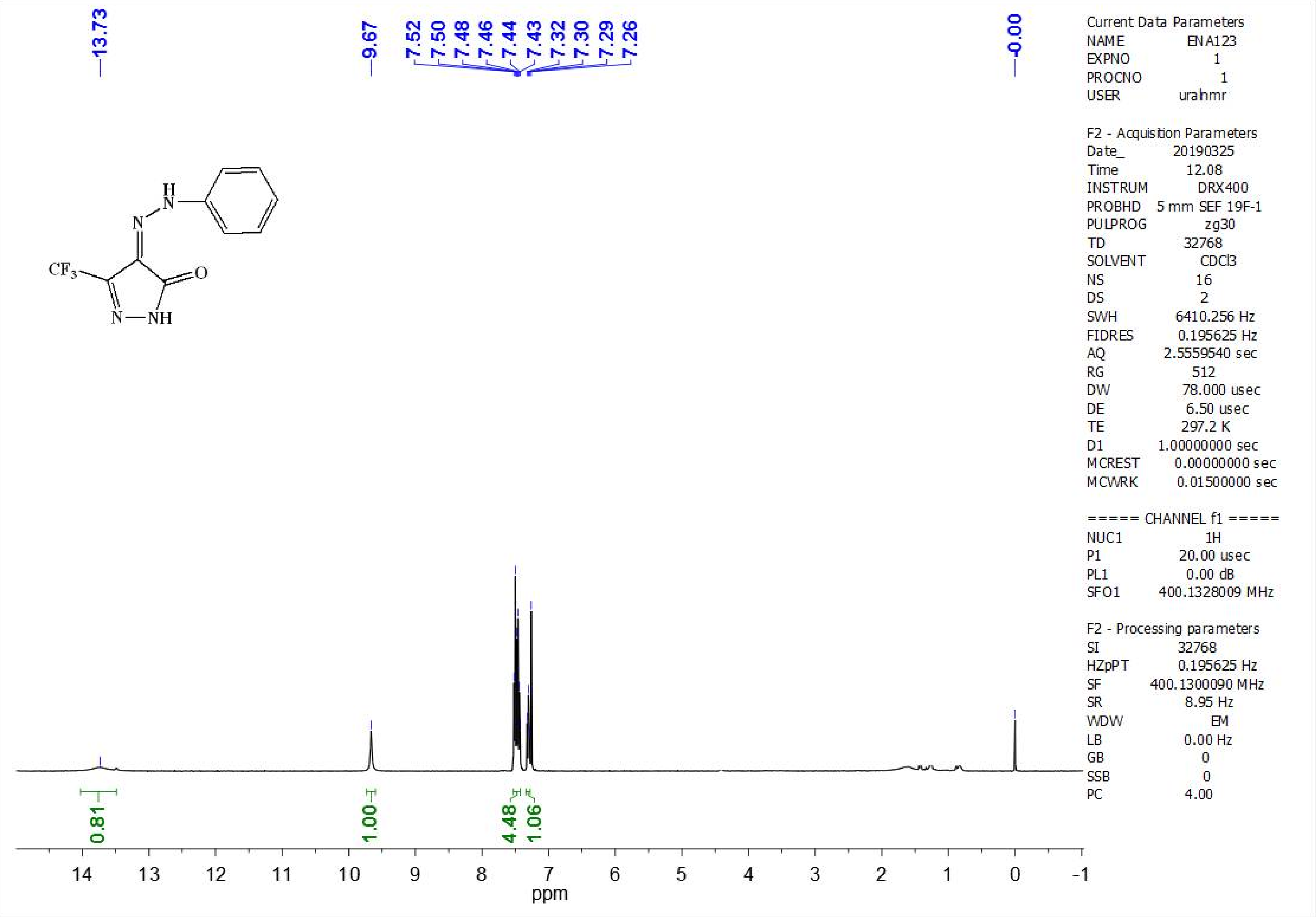

Figure S4. ^{13}C NMR spectrum of compound **5a**

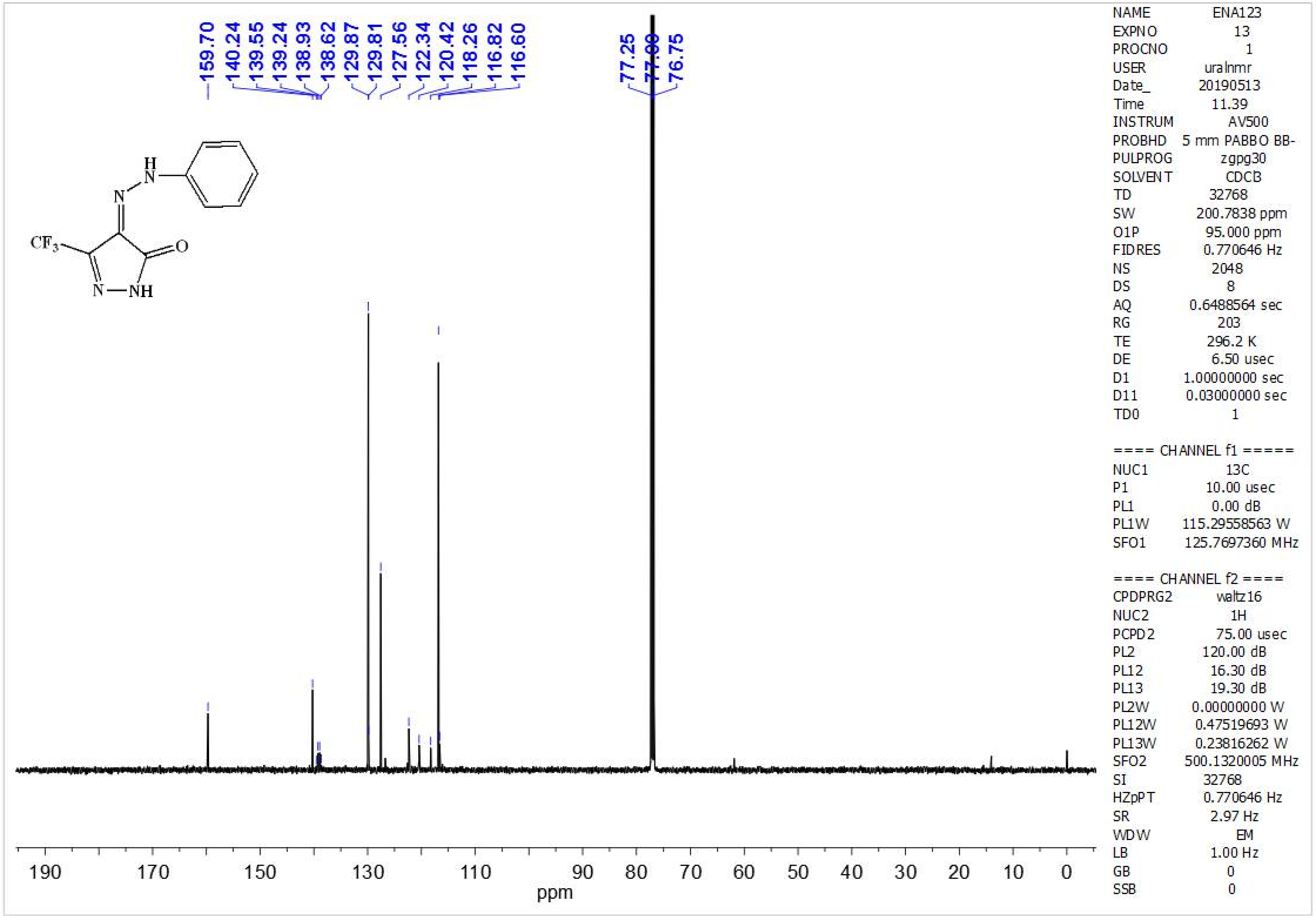

Figure S5. ^{19}F NMR spectrum of compound **5a**

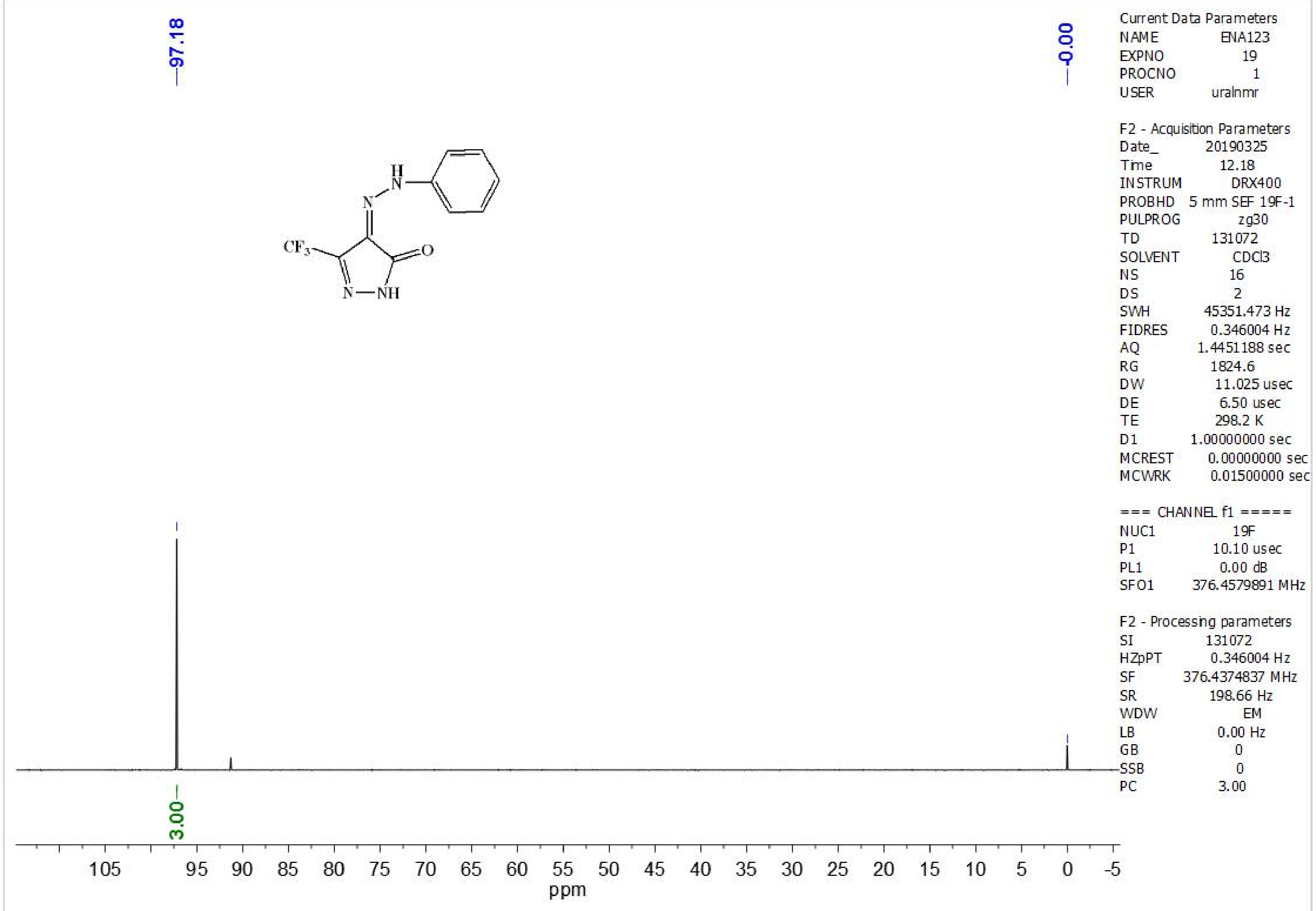

Figure S6. ¹H NMR spectrum of compound **5b**

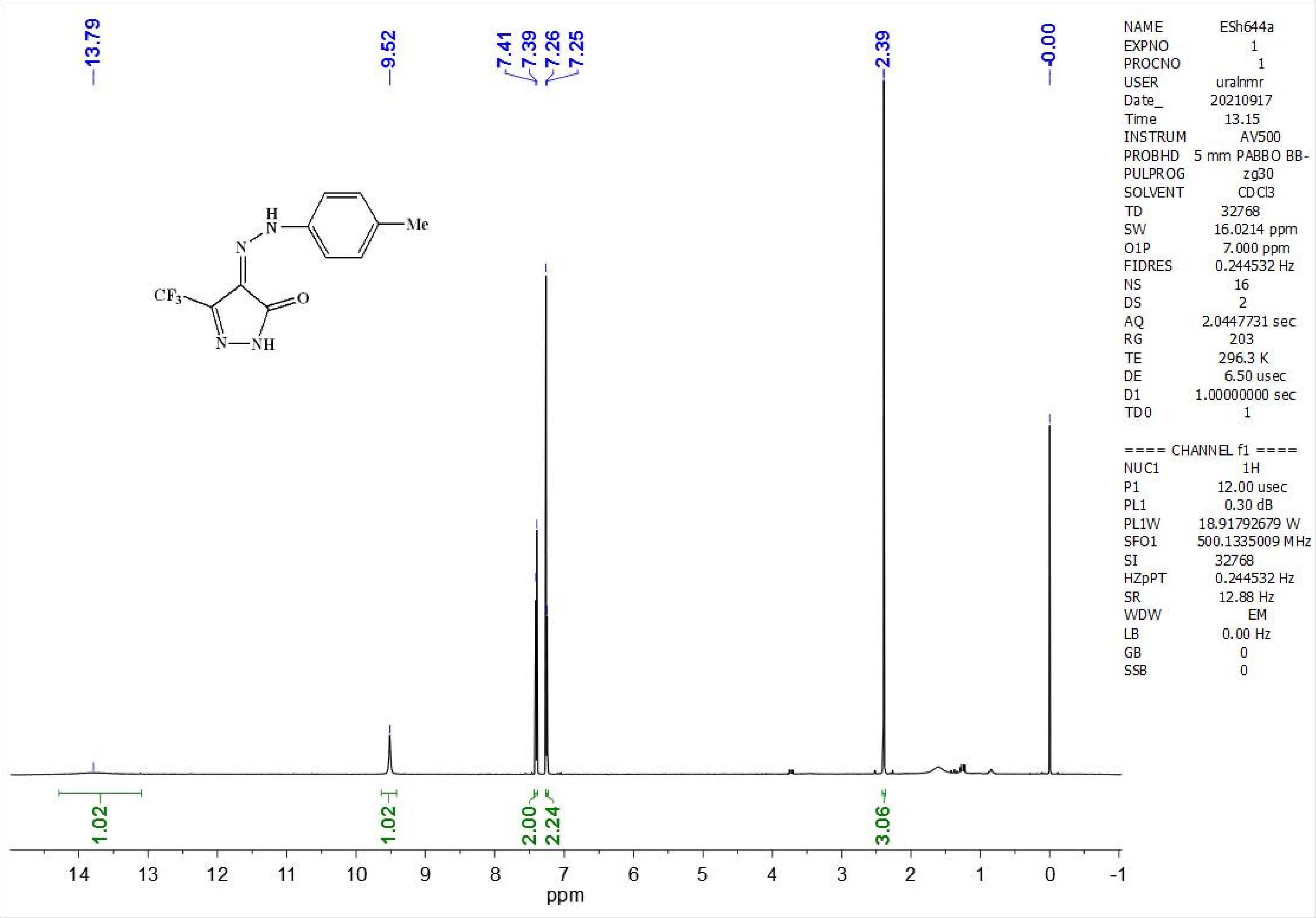

Figure S7. ^{19}F NMR spectrum of compound **5b**

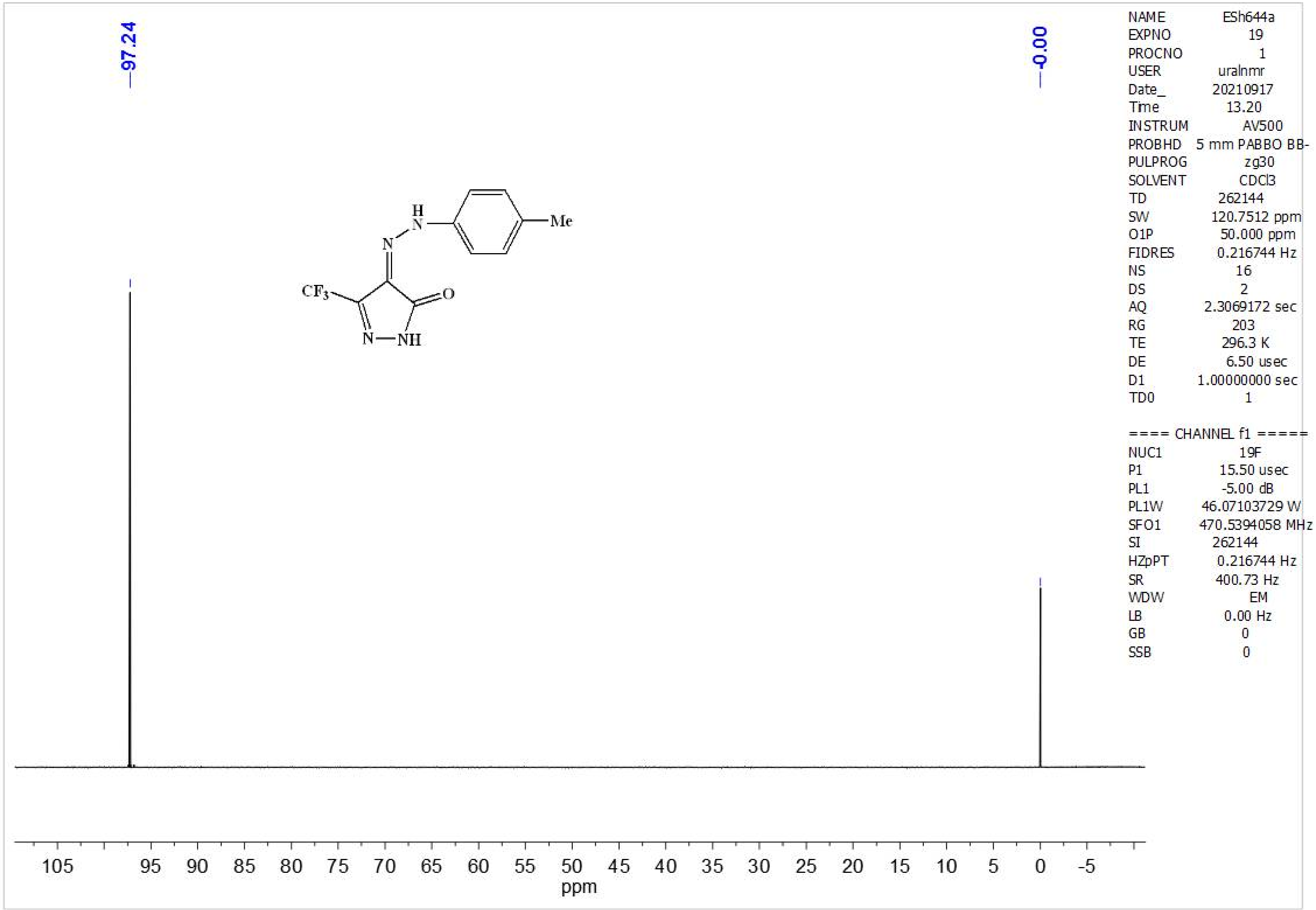

Figure S8. ^{1}H NMR spectrum of compound **5c**

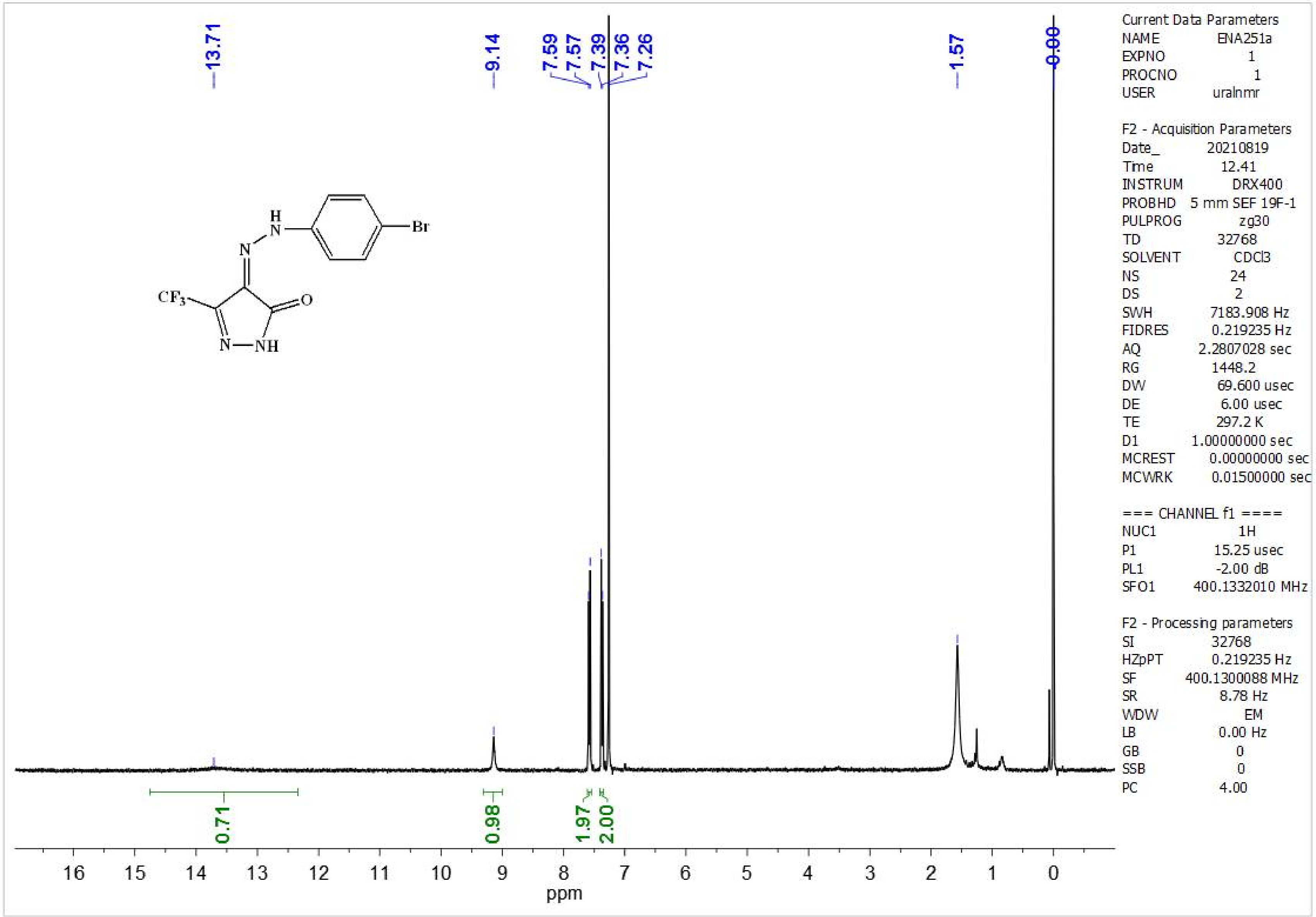

Figure S9. ^{13}C NMR spectrum of compound **5c**

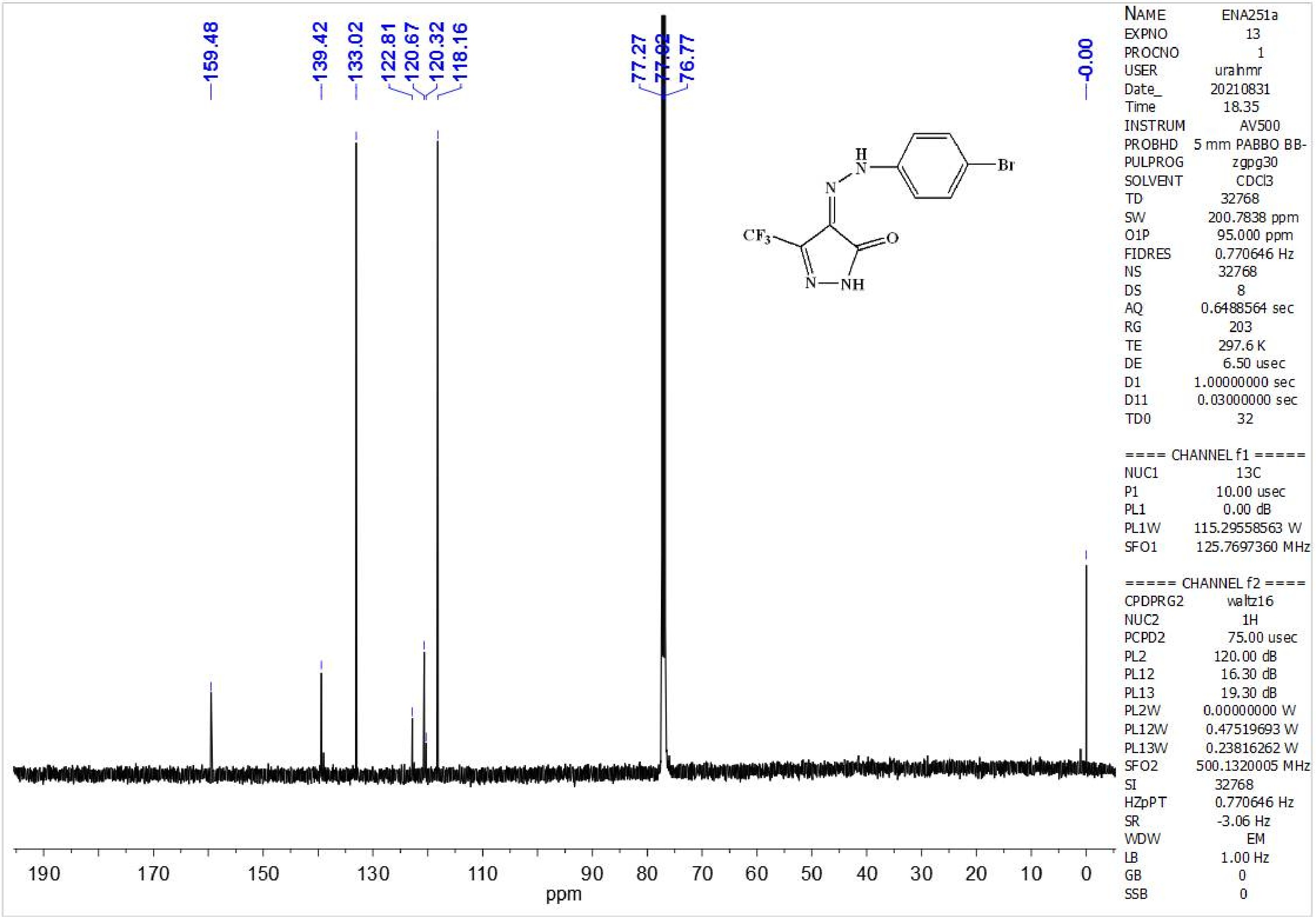

Figure S10. ^{19}F NMR spectrum of compound **5c**

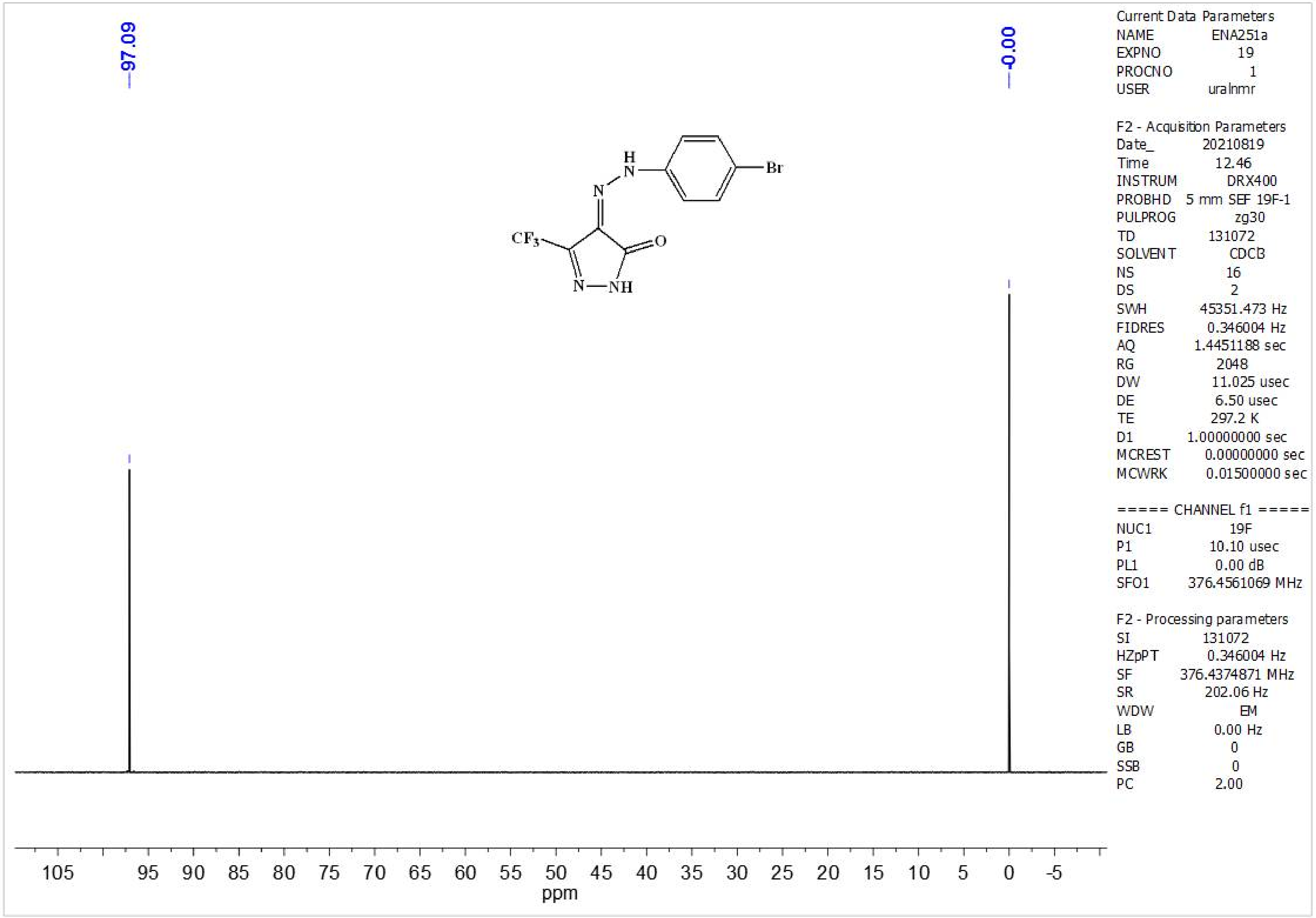

Figure S11. ^{1}H NMR spectrum of compound **5d**

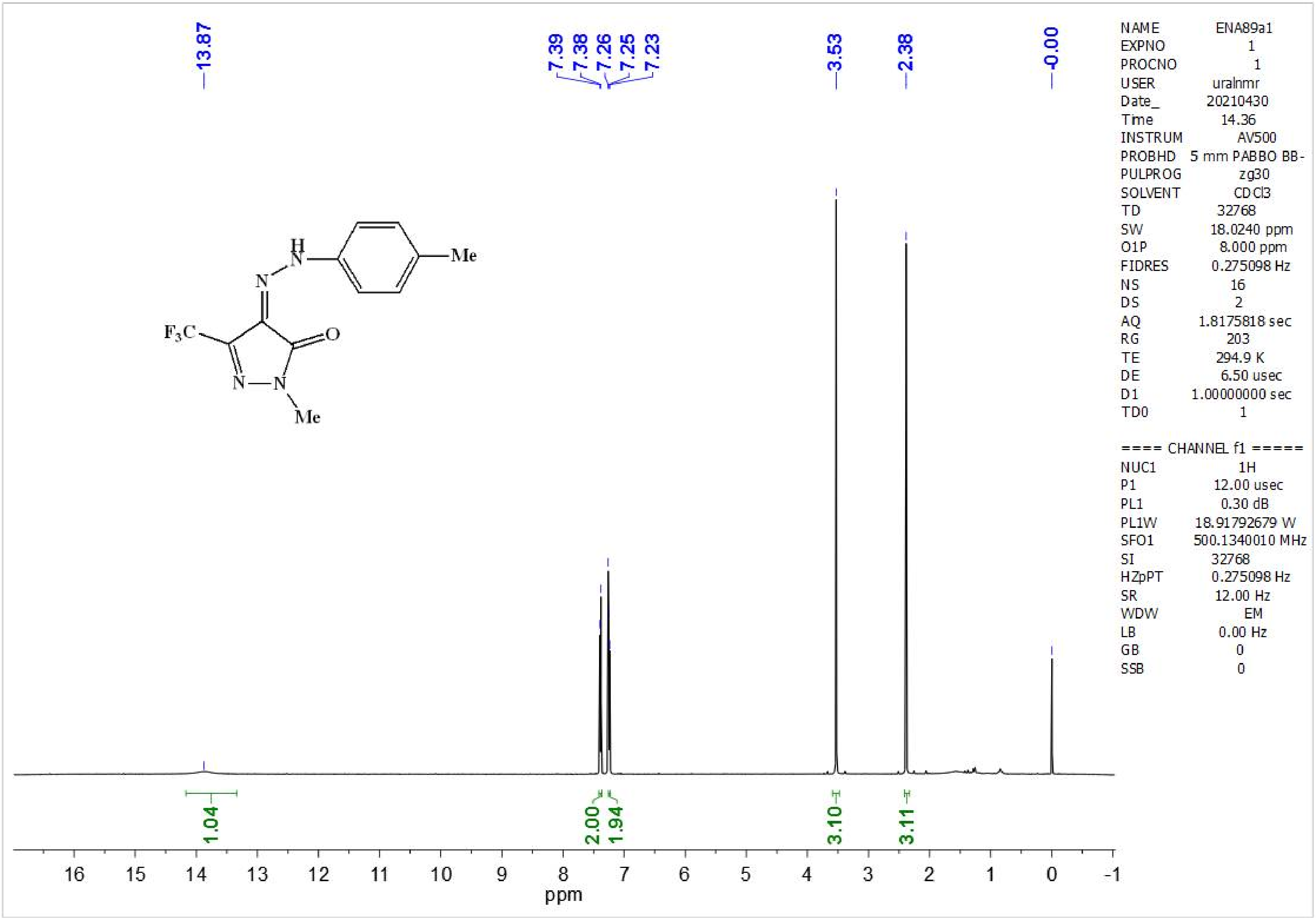

Figure S12. ¹⁹F NMR spectrum of compound **5d**

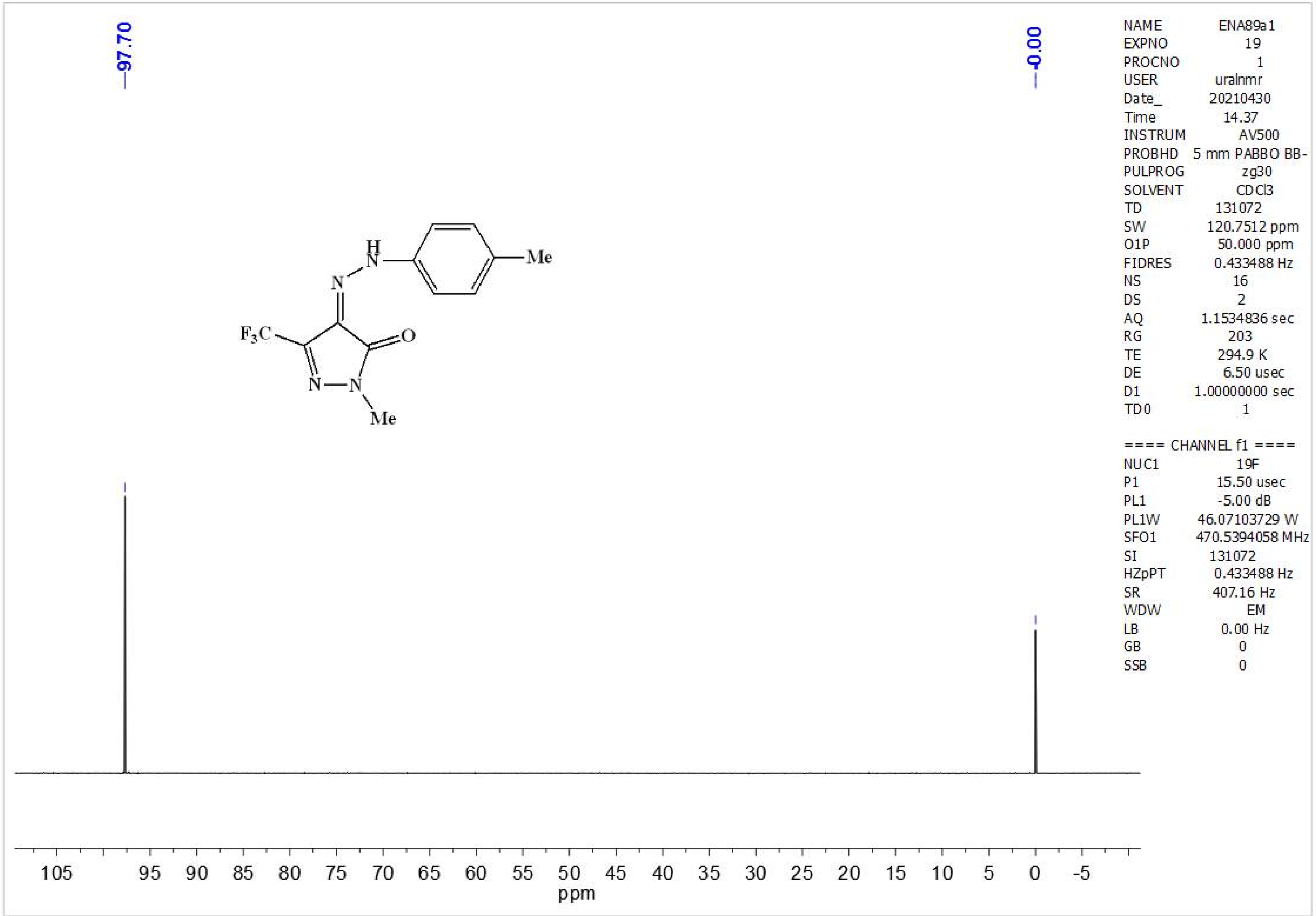

Figure S13. ^{1}H NMR spectrum of compound **5e**

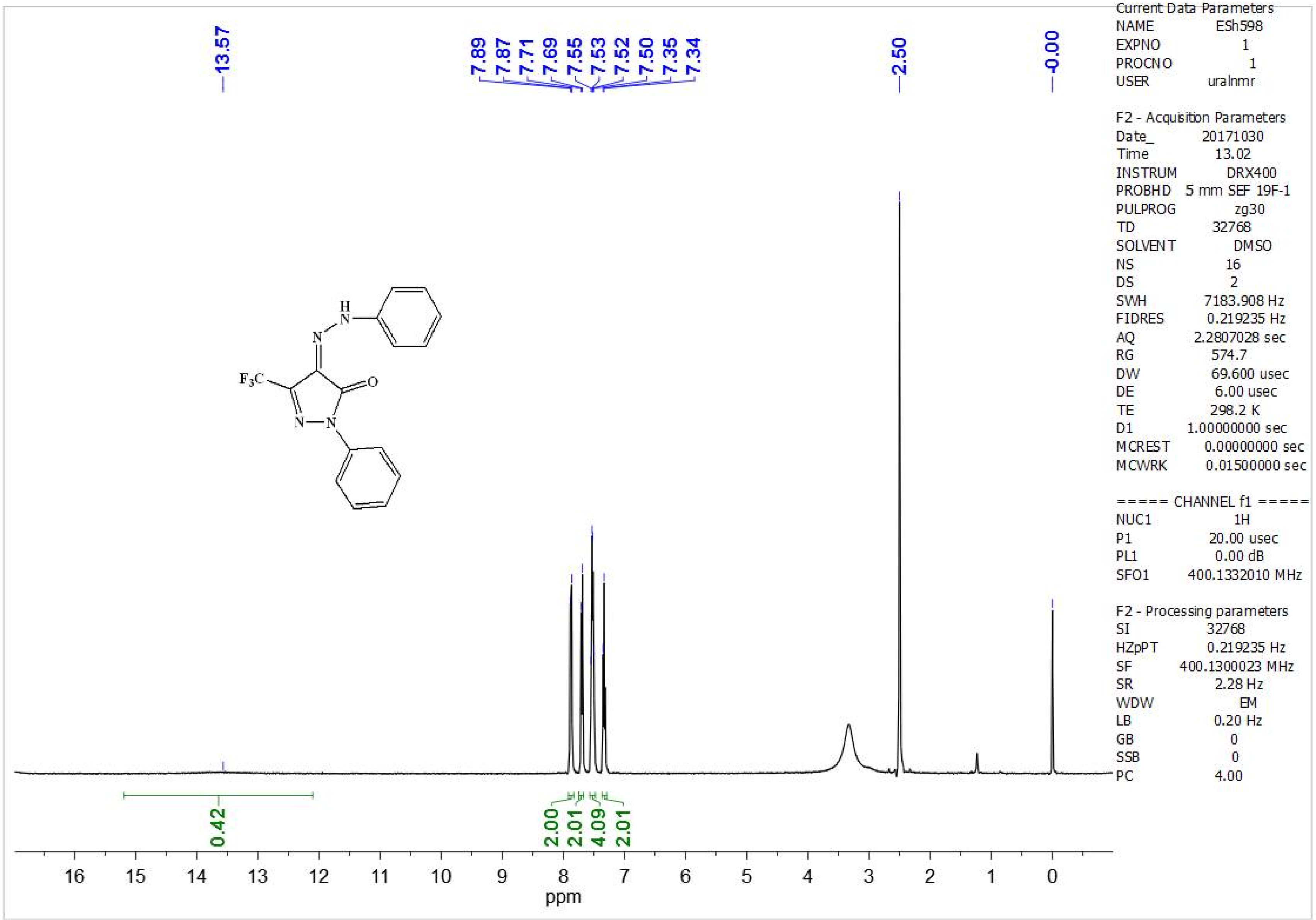

Figure S14. ^{19}F NMR spectrum of compound **5e**

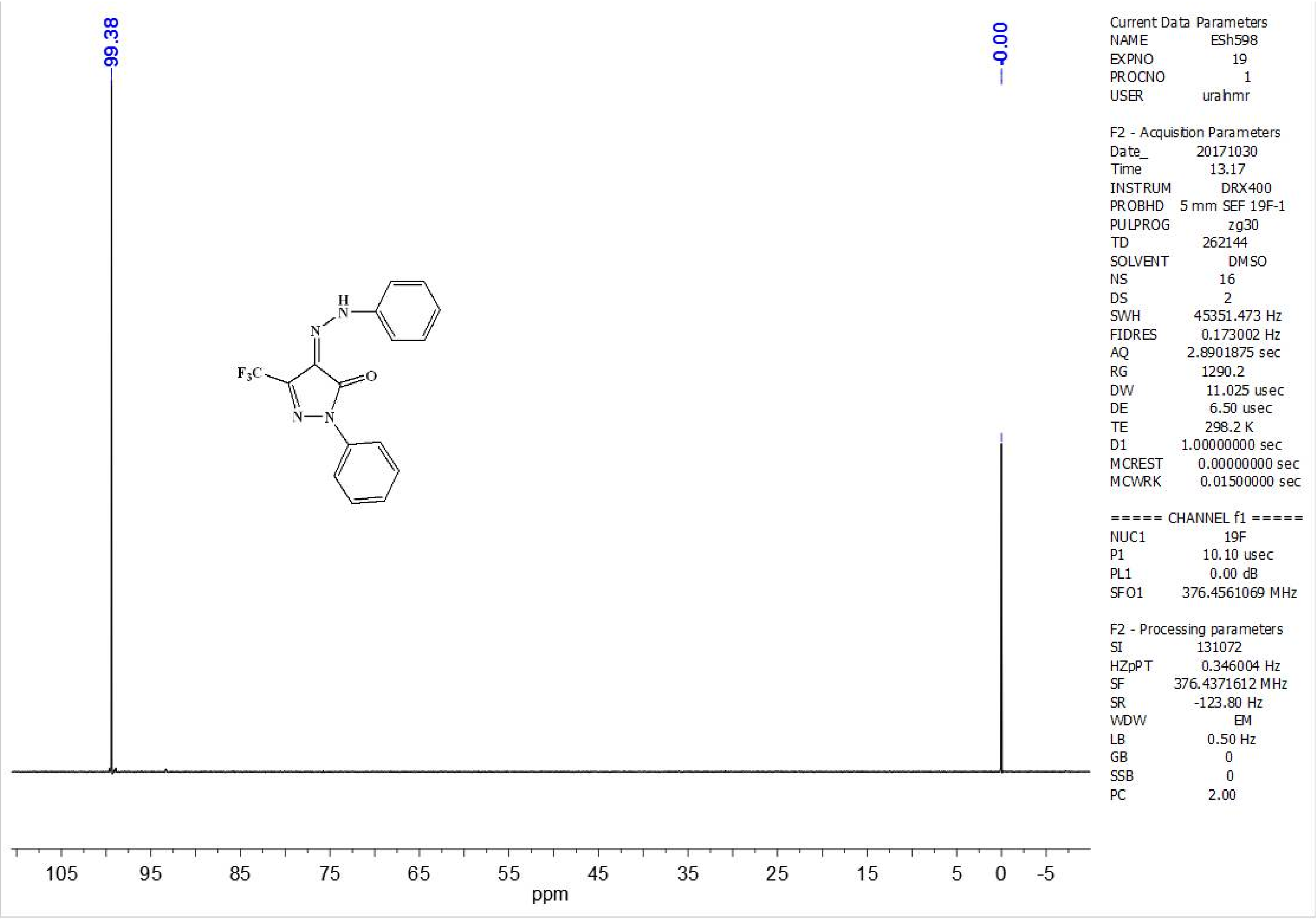

Figure S15. ^{1}H NMR spectrum of compound **5f**

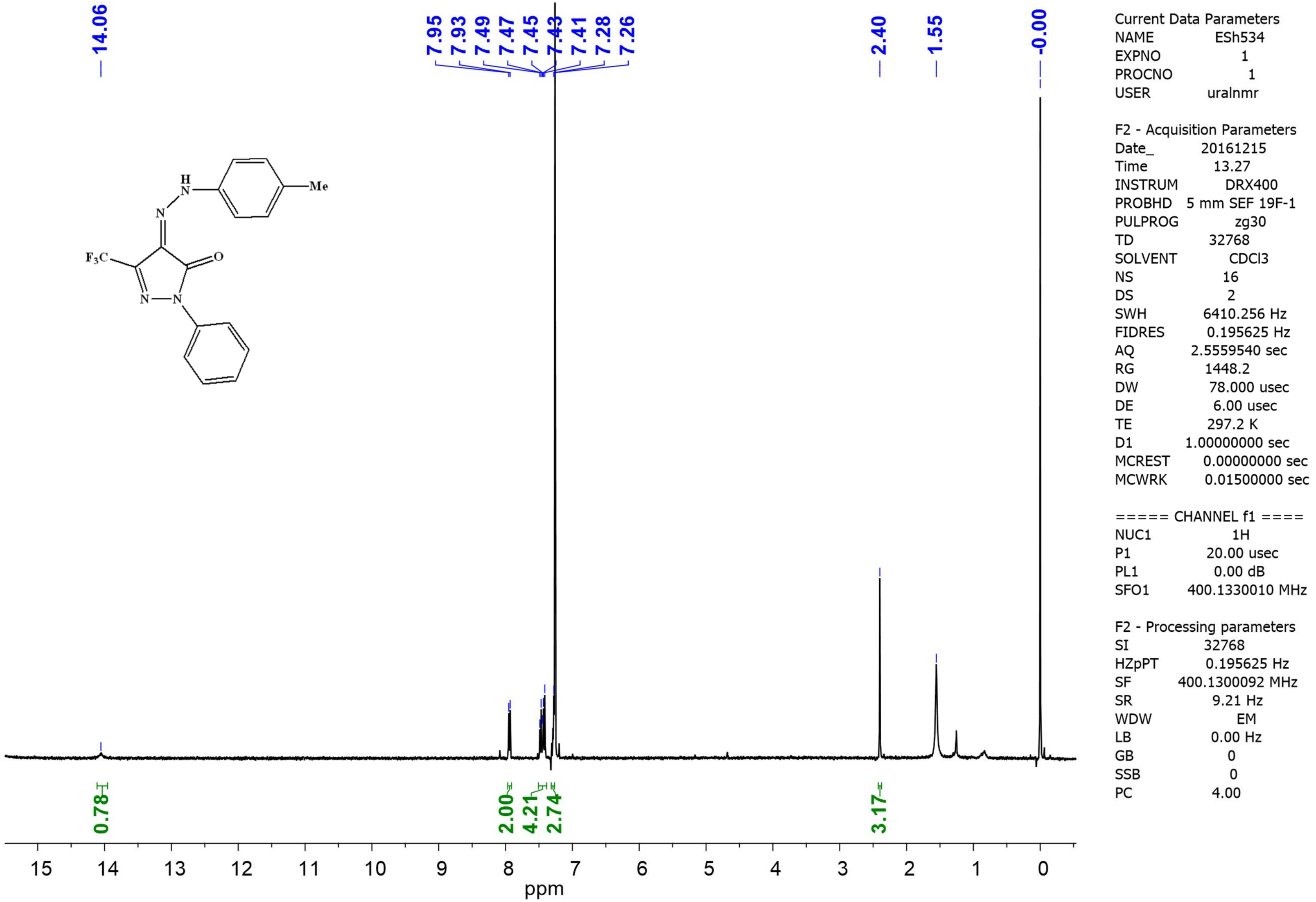

Figure S16. ^{13}C NMR spectrum of compound **5f**

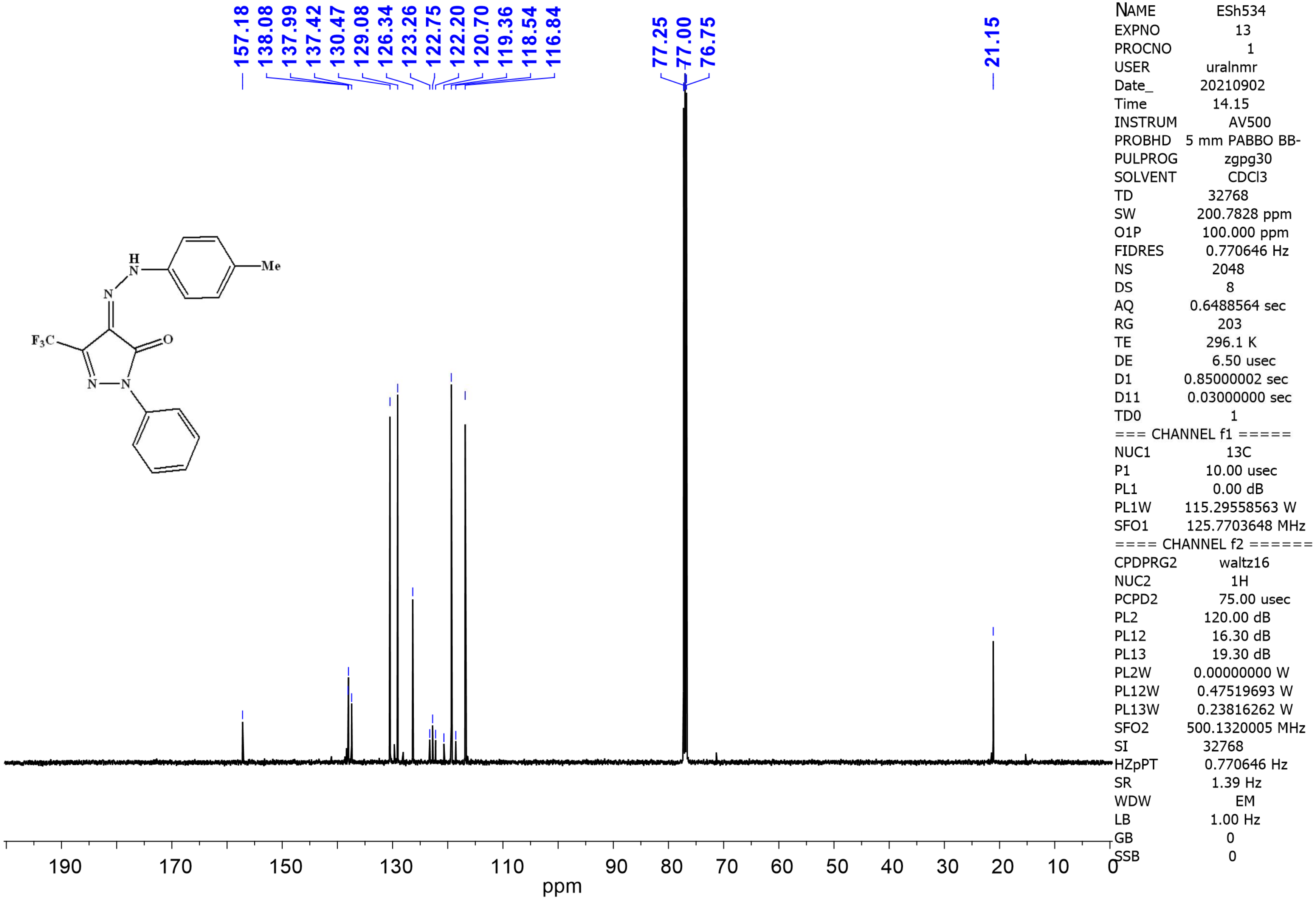

Figure S17. ^{19}F NMR spectrum of compound **5f**

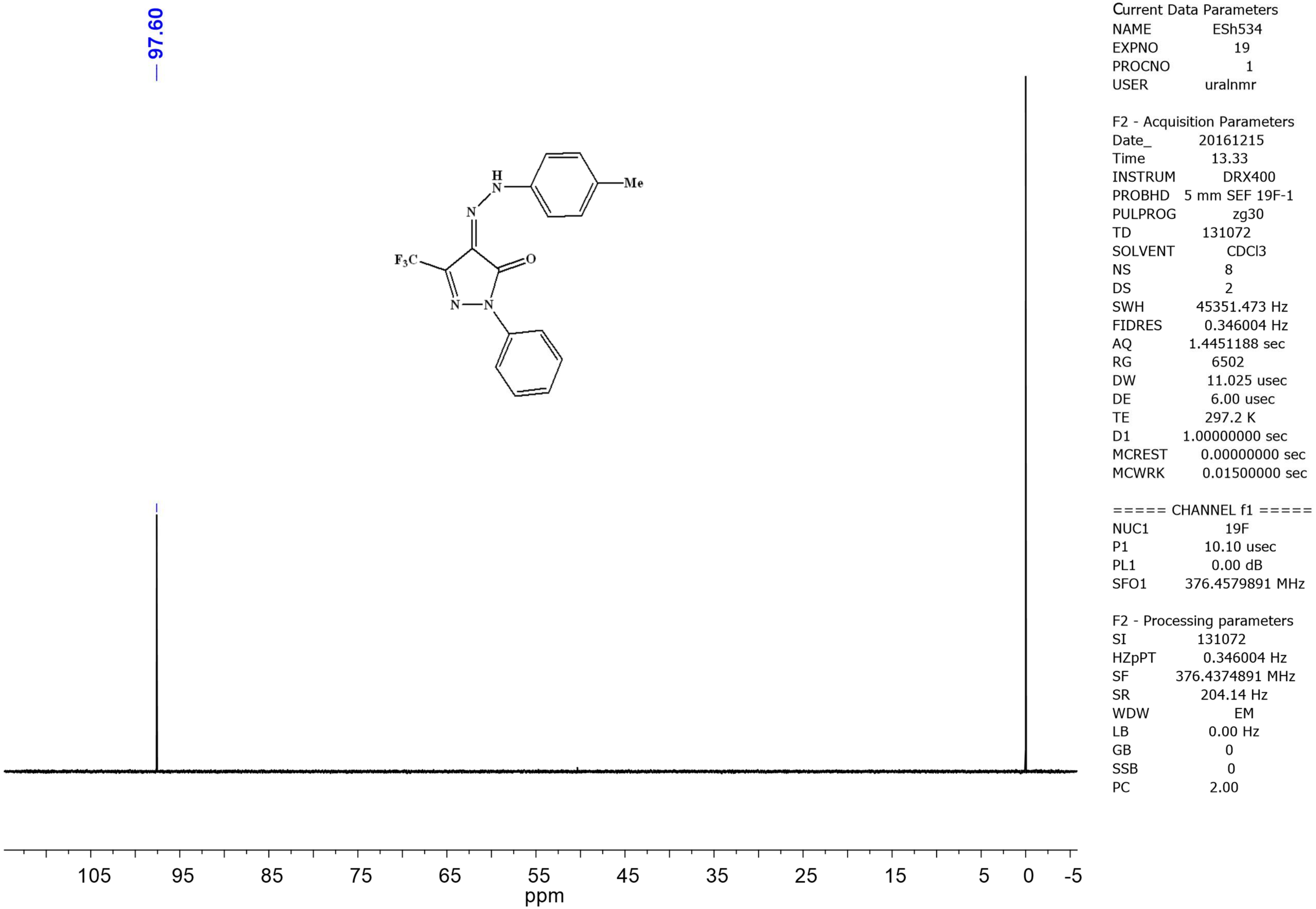

Figure S18. ^{1}H NMR spectrum of compound **5g**

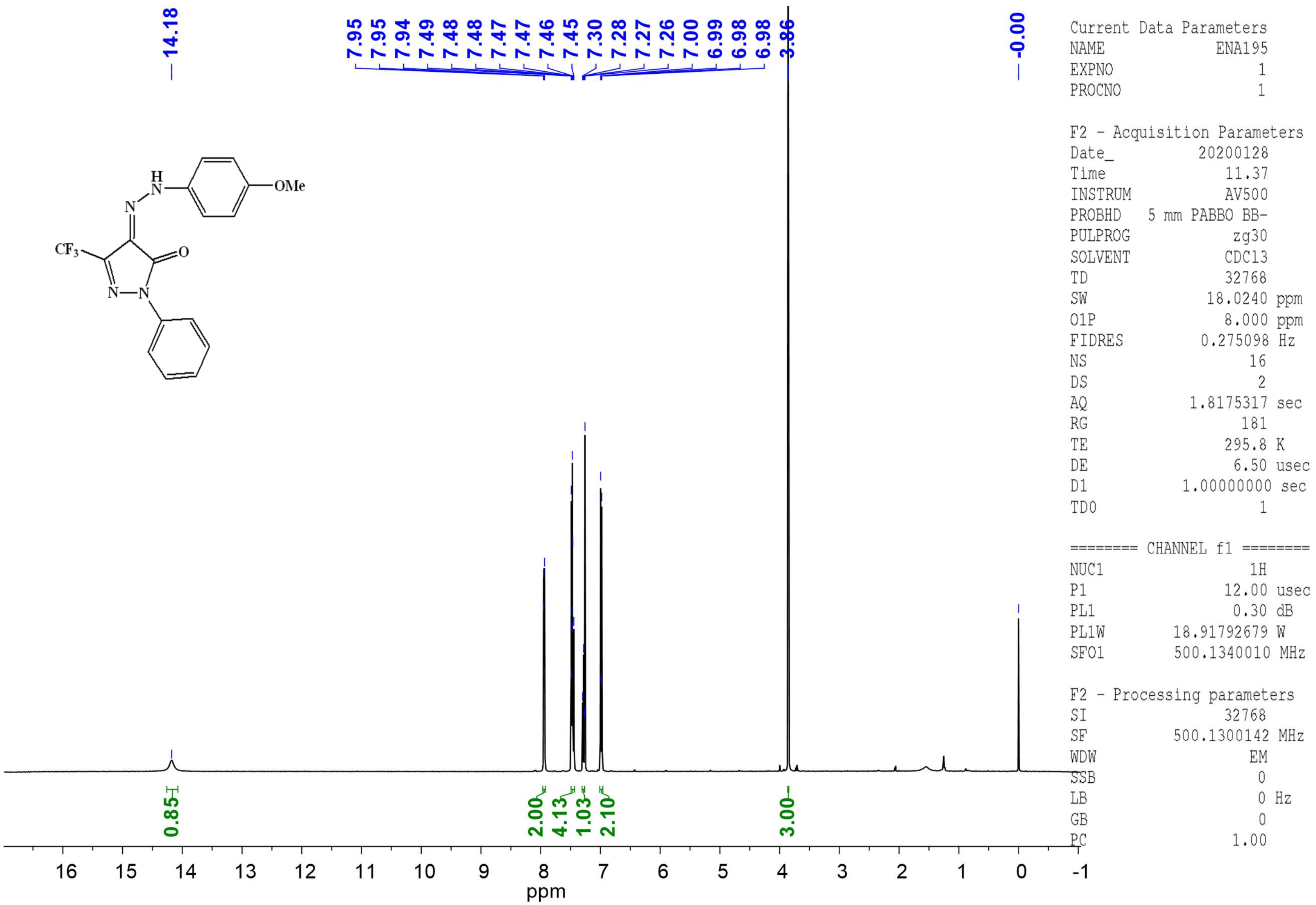

Figure S19. ^{13}C NMR spectrum of compound **5g**

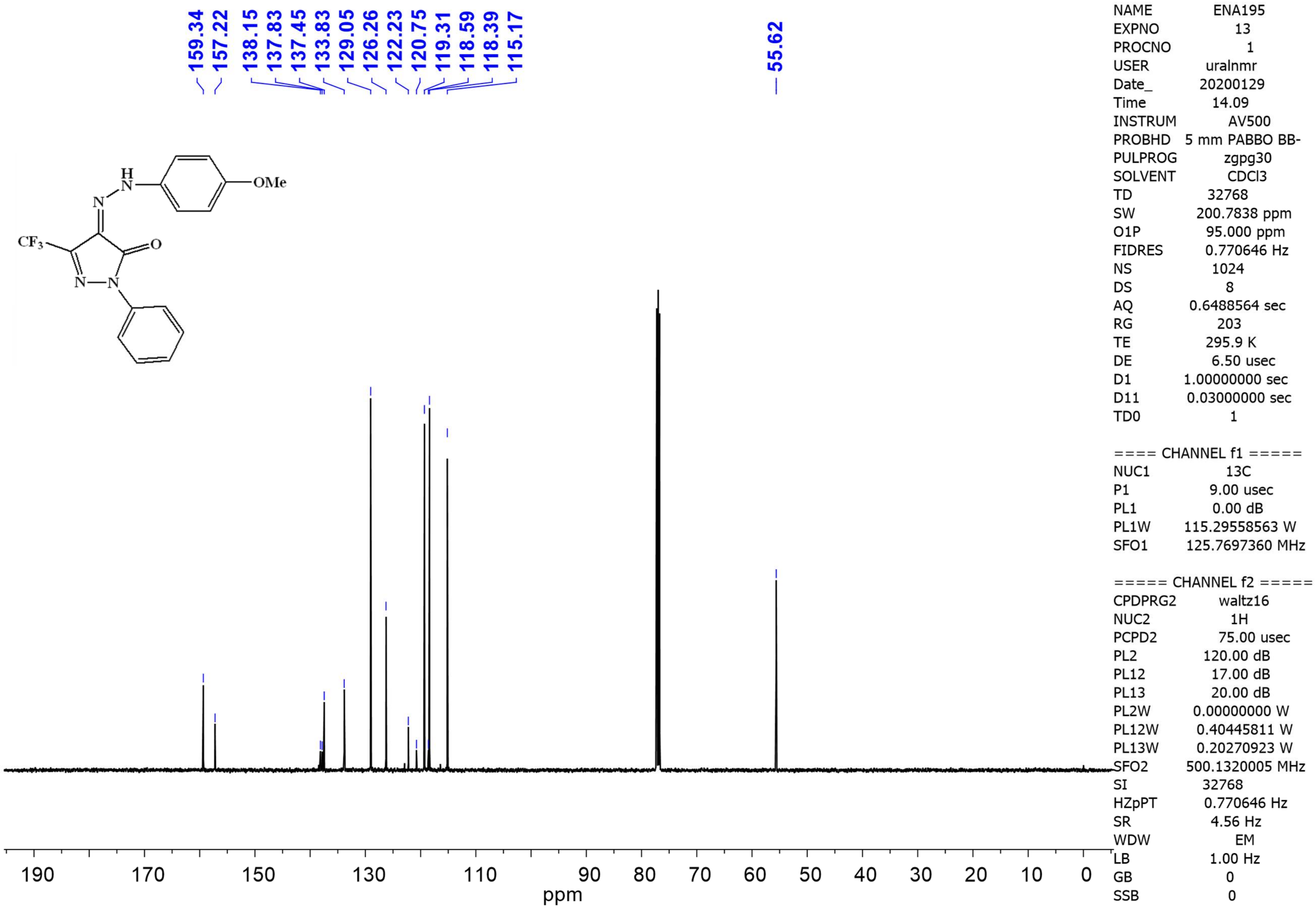

Figure S20. ^{19}F NMR spectrum of compound **5g**

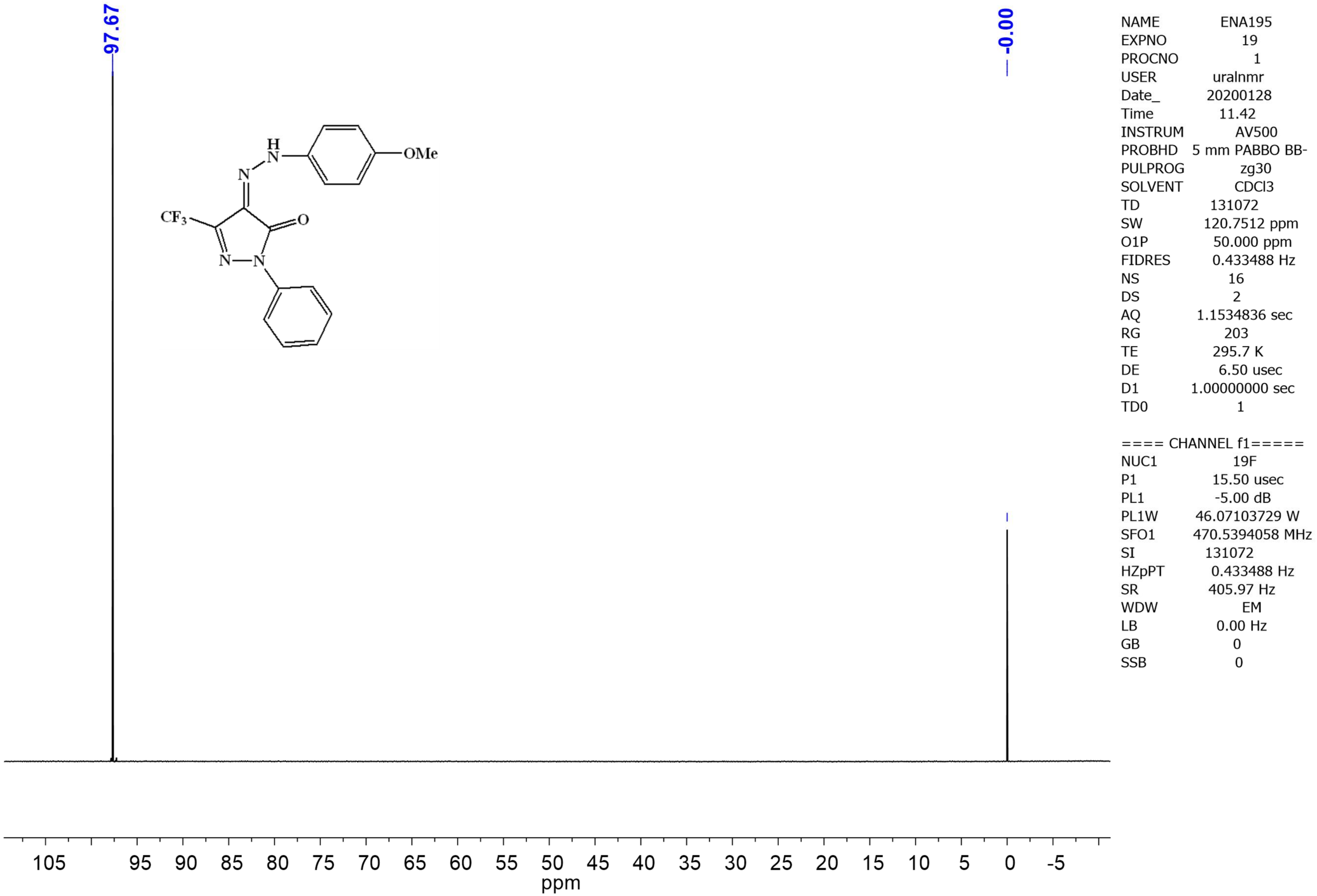

Figure S21. ^{1}H NMR spectrum of compound **5h**

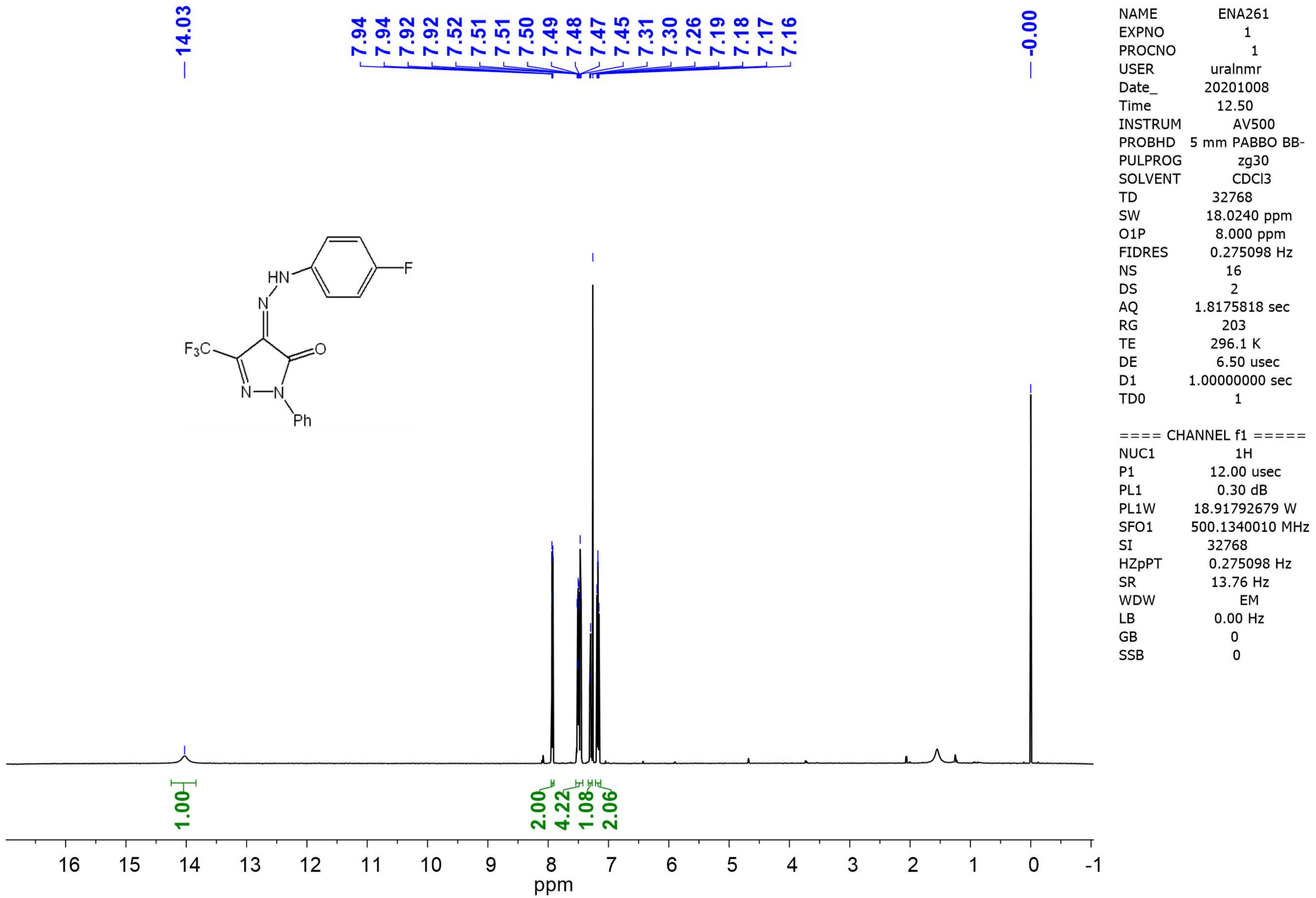

Figure S22. ^{13}C NMR spectrum of compound **5h**

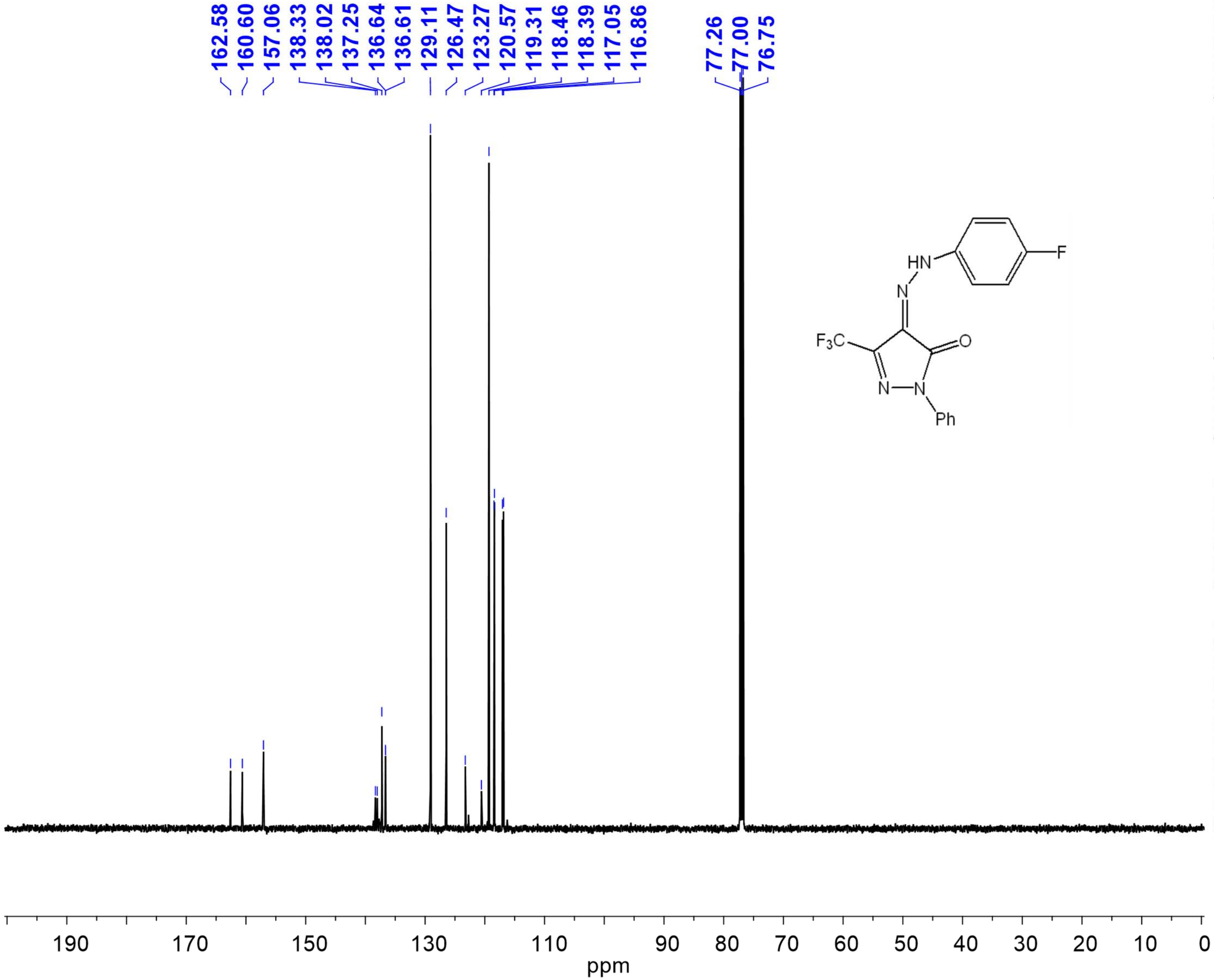

NAME	ENA261
EXPNO	13
PROCNO	1
USER	uralnmr
Date_	20201015
Time	15.00
INSTRUM	AV500
PROBHD	5 mm PABBO BB-
PULPROG	zgpg30
SOLVENT	CDCl3
TD	32768
SW	200.7828 ppm
O1P	100.000 ppm
FIDRES	0.770646 Hz
NS	1024
DS	8
AQ	0.6488564 sec
RG	203
TE	296.9 K
DE	6.50 usec
D1	1.00000000 sec
D11	0.03000000 sec
TD0	1
==== CHANNEL f1 =====	
NUC1	13C
P1	9.00 usec
PL1	0.00 dB
PL1W	115.29558563 W
SFO1	125.7703648 MHz
===== CHANNEL f2 ======	
CPDPRG2	waltz16
NUC2	1H
PCPD2	75.00 usec
PL2	120.00 dB
PL12	17.00 dB
PL13	20.00 dB
PL2W	0.00000000 W
PL12W	0.40445811 W
PL13W	0.20270923 W
SFO2	500.1320005 MHz
SI	32768
HZpPT	0.770646 Hz
SR	2.97 Hz
WDW	EM
LB	1.00 Hz
GB	0
SSB	0

Figure S23. ^{19}F NMR spectrum of compound **5h**

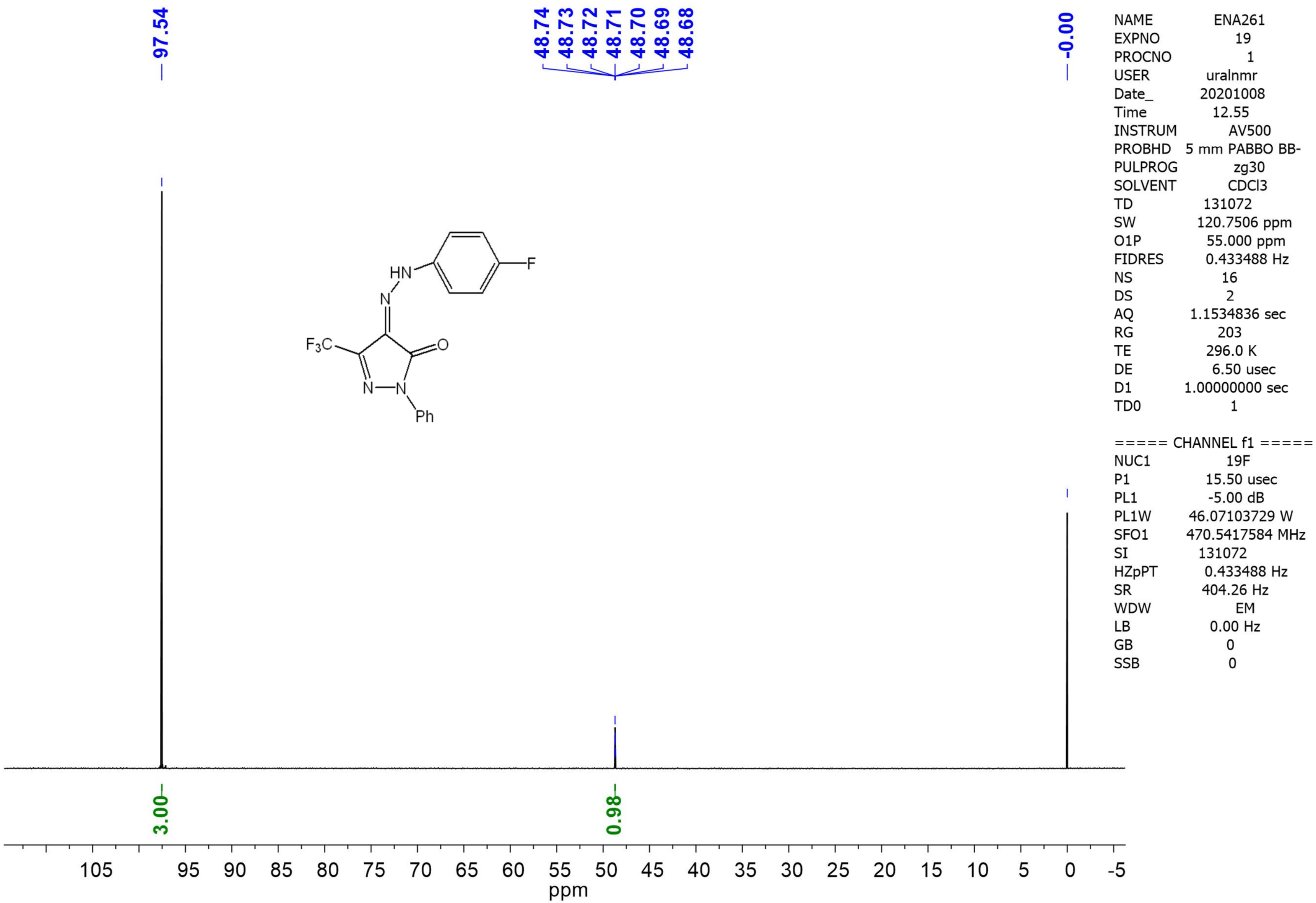

Figure S24. ^{1}H NMR spectrum of compound **5i**

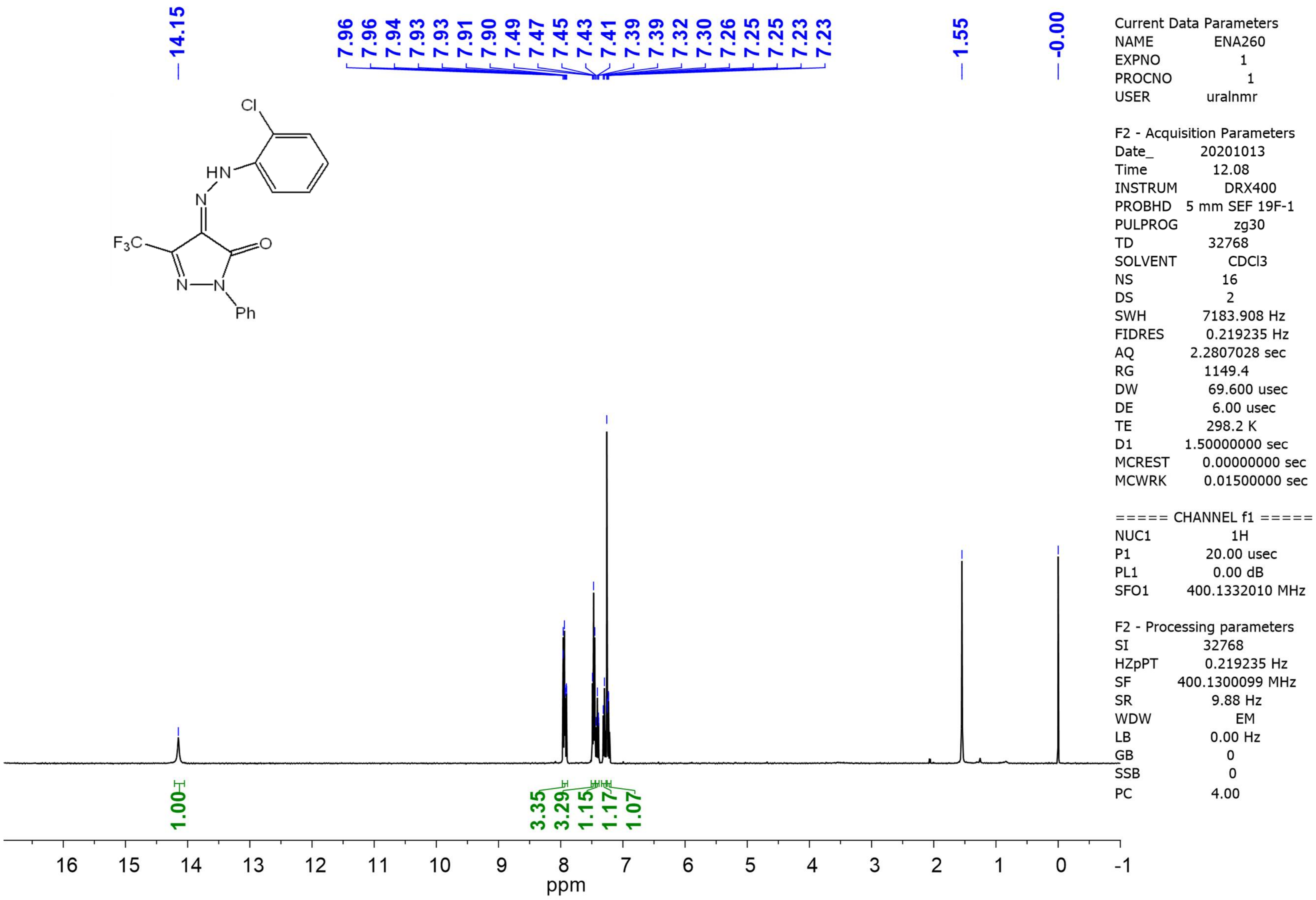

Figure S25. ^{13}C NMR spectrum of compound **5i**

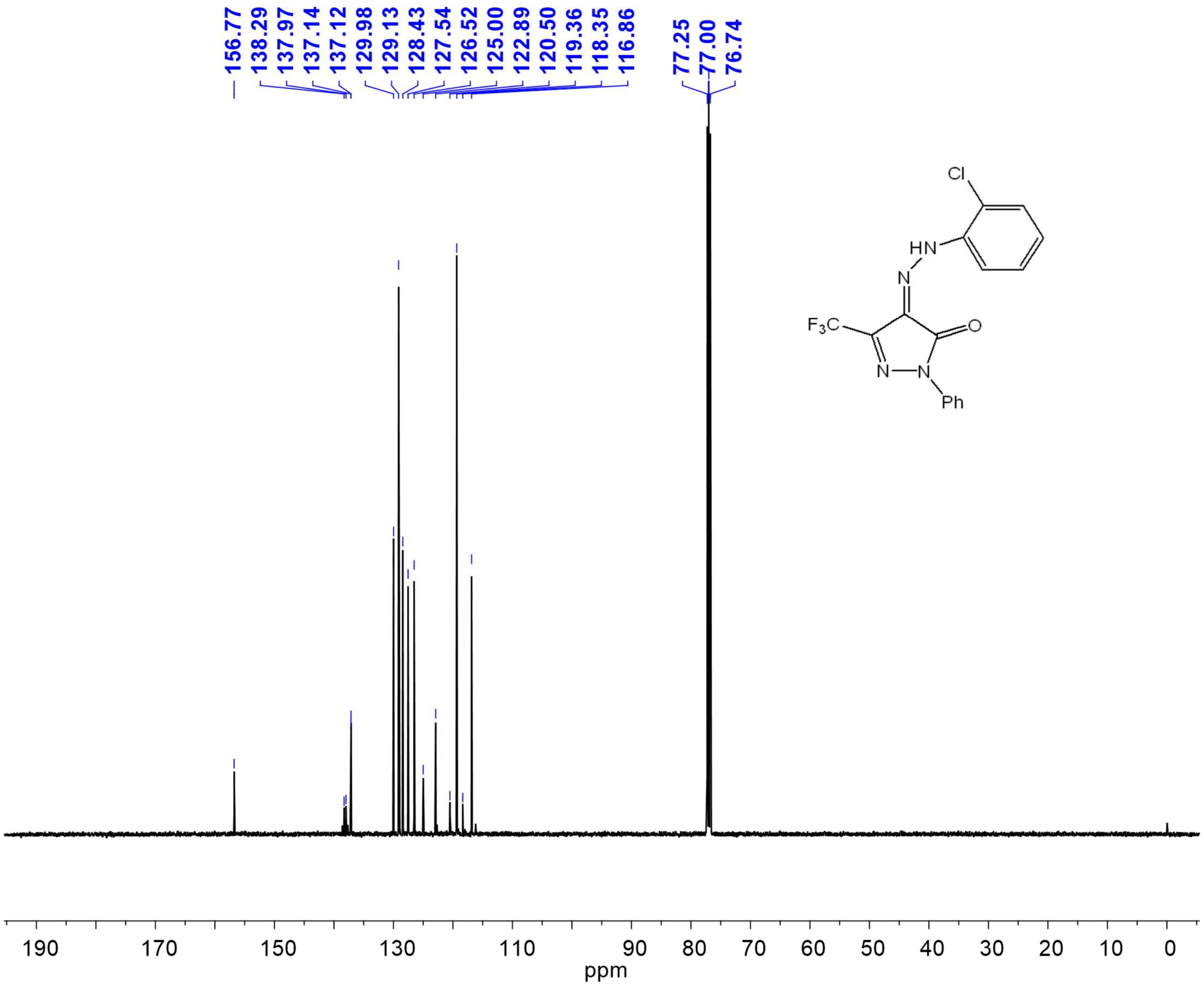

NAME ENA260
EXPNO 13
PROCNO 1
USER uralnmr
Date_ 20201016
Time 14.59
INSTRUM AV500
PROBHD 5 mm PABBO BB-
PULPROG zgpg30
SOLVENT CDCl3
TD 32768
SW 200.7838 ppm
O1P 95.000 ppm
FIDRES 0.770646 Hz
NS 2048
DS 8
AQ 0.6488564 sec
RG 203
TE 296.5 K
DE 6.50 usec
D1 0.85000002 sec
D11 0.03000000 sec
TD0 1

==== CHANNEL f1 =====
NUC1 13C
P1 9.00 usec
PL1 0.00 dB
PL1W 115.29558563 W
SFO1 125.7697360 MHz

==== CHANNEL f2 ====
CPDPRG2 waltz16
NUC2 1H
PCPD2 75.00 usec
PL2 120.00 dB
PL12 17.00 dB
PL13 20.00 dB
PL2W 0.00000000 W
PL12W 0.40445811 W
PL13W 0.20270923 W
SFO2 500.1320005 MHz
SI 32768
HZpPT 0.770646 Hz
SR 3.09 Hz
WDW EM
LB 1.00 Hz
GB 0
SSB 0

Figure S26. ^{19}F NMR spectrum of compound **5i**

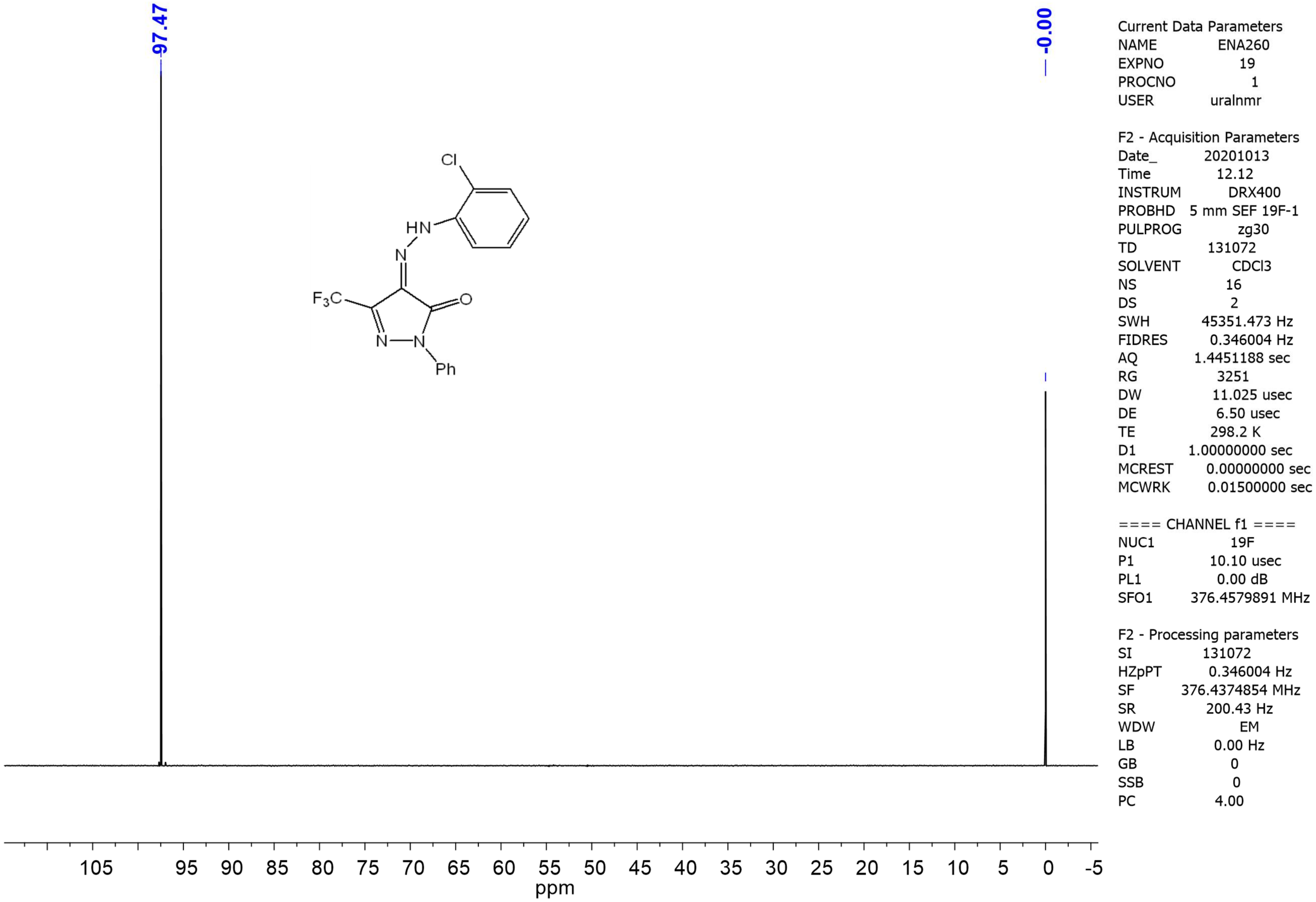

Figure S27. ¹H NMR spectrum of compound **5j**

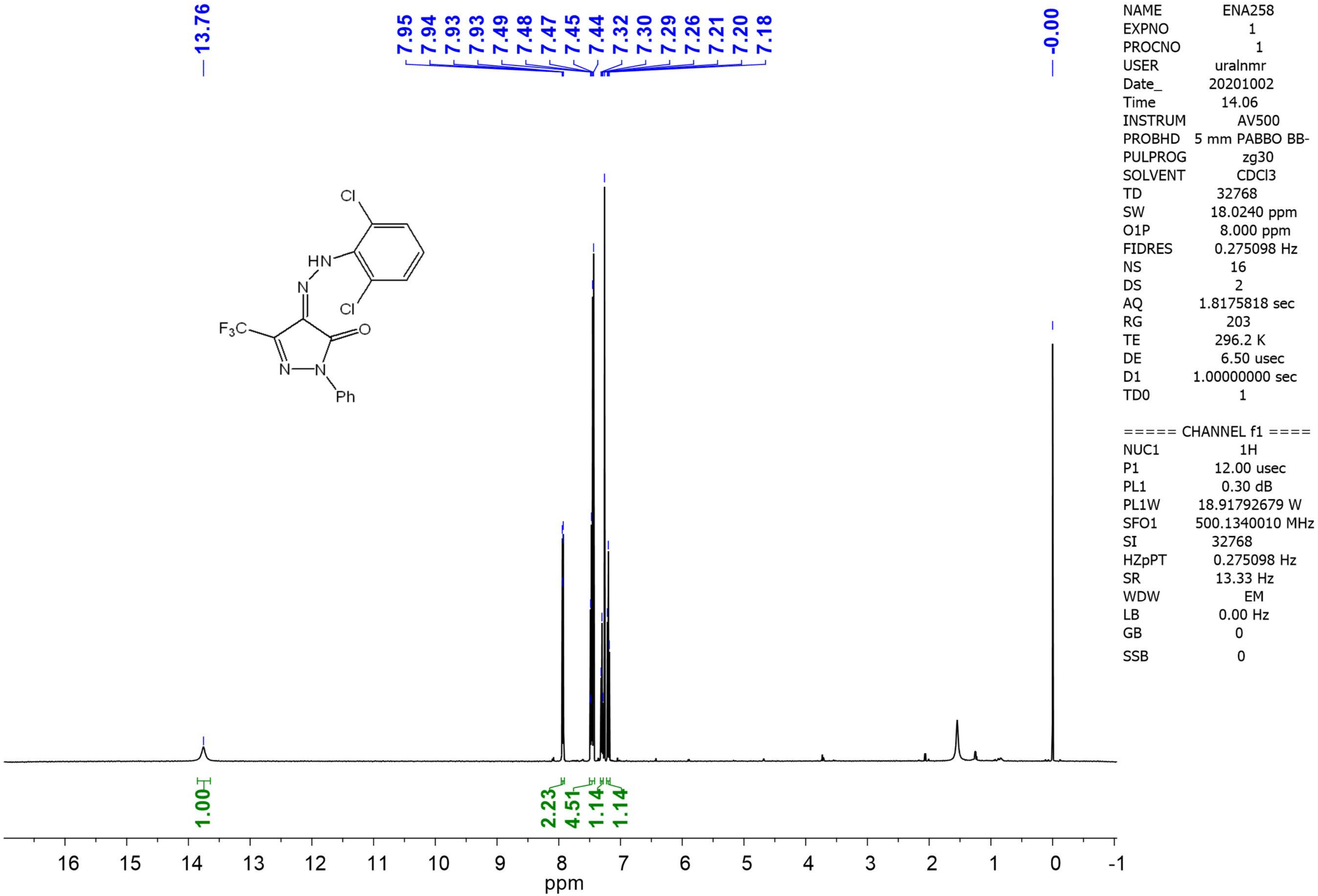

Figure S28. ¹³C NMR spectrum of compound **5j**

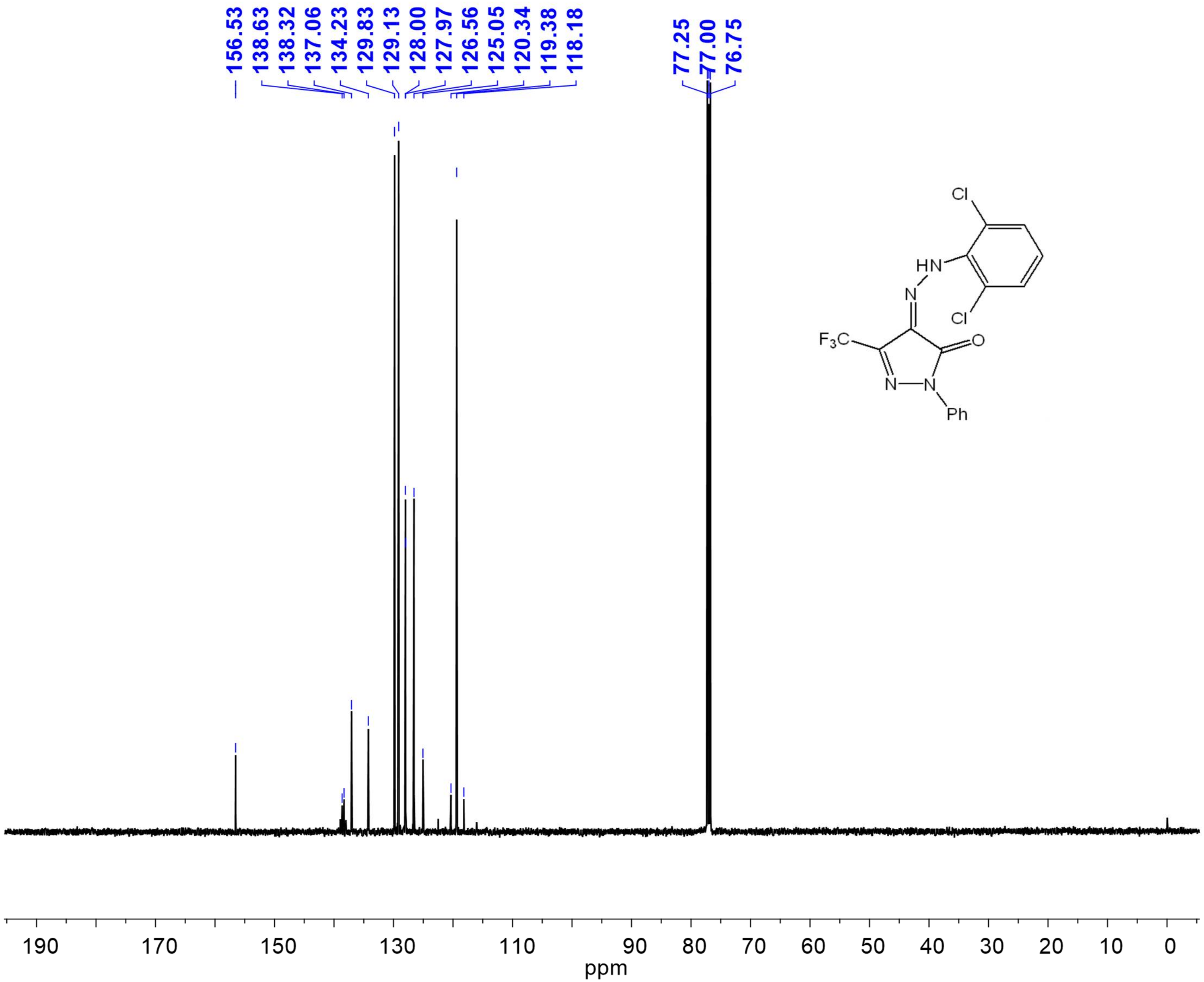

NAME ENA258
EXPNO 13
PROCNO 1
USER uralnmr
Date_ 20201008
Time 11.07
INSTRUM AV500
PROBHD 5 mm PABBO BB-
PULPROG zgpg30
SOLVENT CDCl3
TD 32768
SW 200.7838 ppm
O1P 95.000 ppm
FIDRES 0.770646 Hz
NS 1024
DS 8
AQ 0.6488564 sec
RG 203
TE 296.8 K
DE 6.50 usec
D1 1.00000000 sec
D11 0.03000000 sec
TD0 1

=== CHANNEL f1 ====
NUC1 13C
P1 9.00 usec
PL1 0.00 dB
PL1W 115.29558563 W
SFO1 125.7697360 MHz

==== CHANNEL f2 ====
CPDPRG2 waltz16
NUC2 1H
PCPD2 75.00 usec
PL2 120.00 dB
PL12 17.00 dB
PL13 20.00 dB
PL2W 0.00000000 W
PL12W 0.40445811 W
PL13W 0.20270923 W
SFO2 500.1320005 MHz
SI 32768
HZpPT 0.770646 Hz
SR 4.06 Hz
WDW EM
LB 1.00 Hz
GB 0
SSB 0

Figure S29. ^{19}F NMR spectrum of compound **5j**

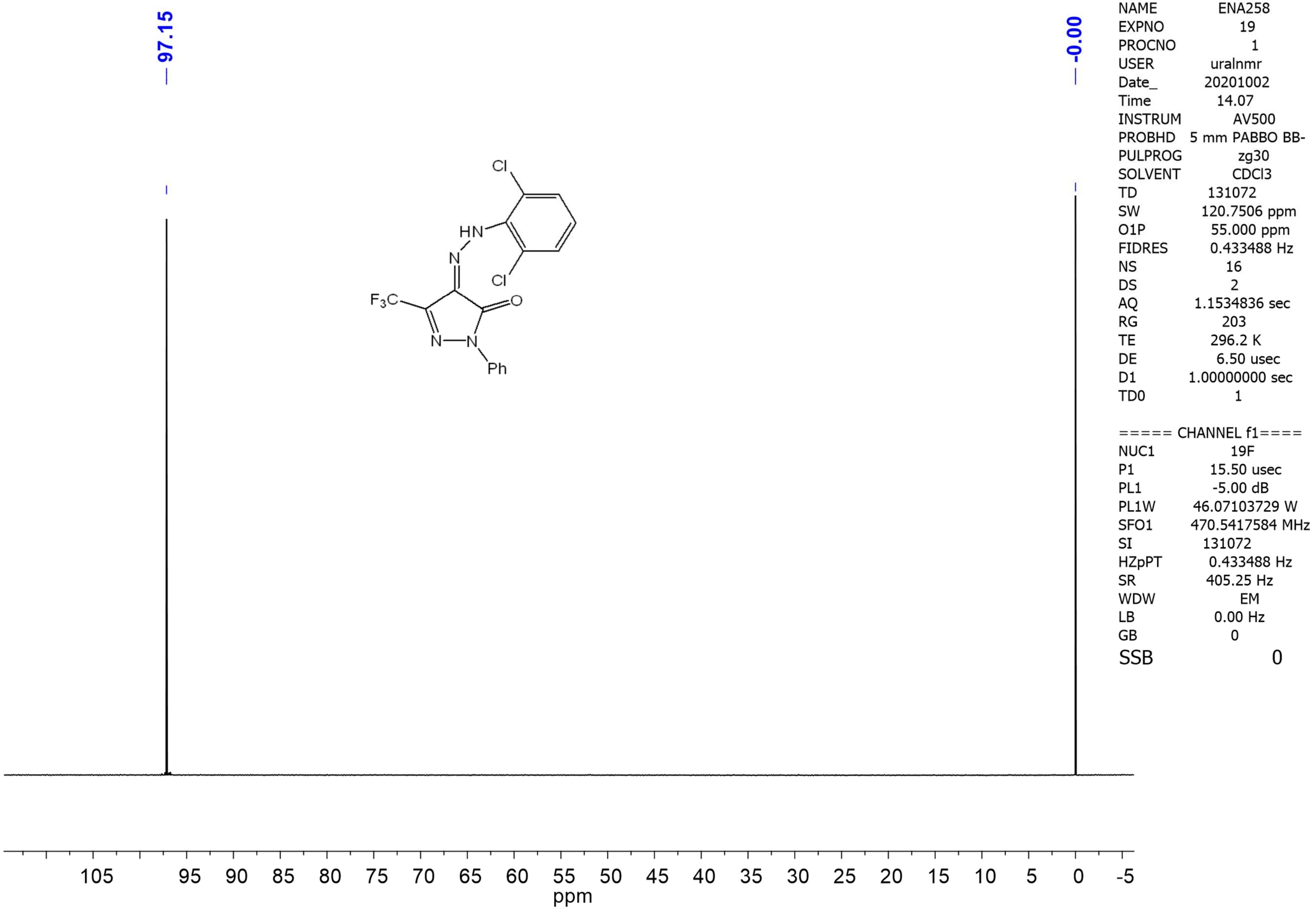

Figure S30. ^{1}H NMR spectrum of compound **5k**

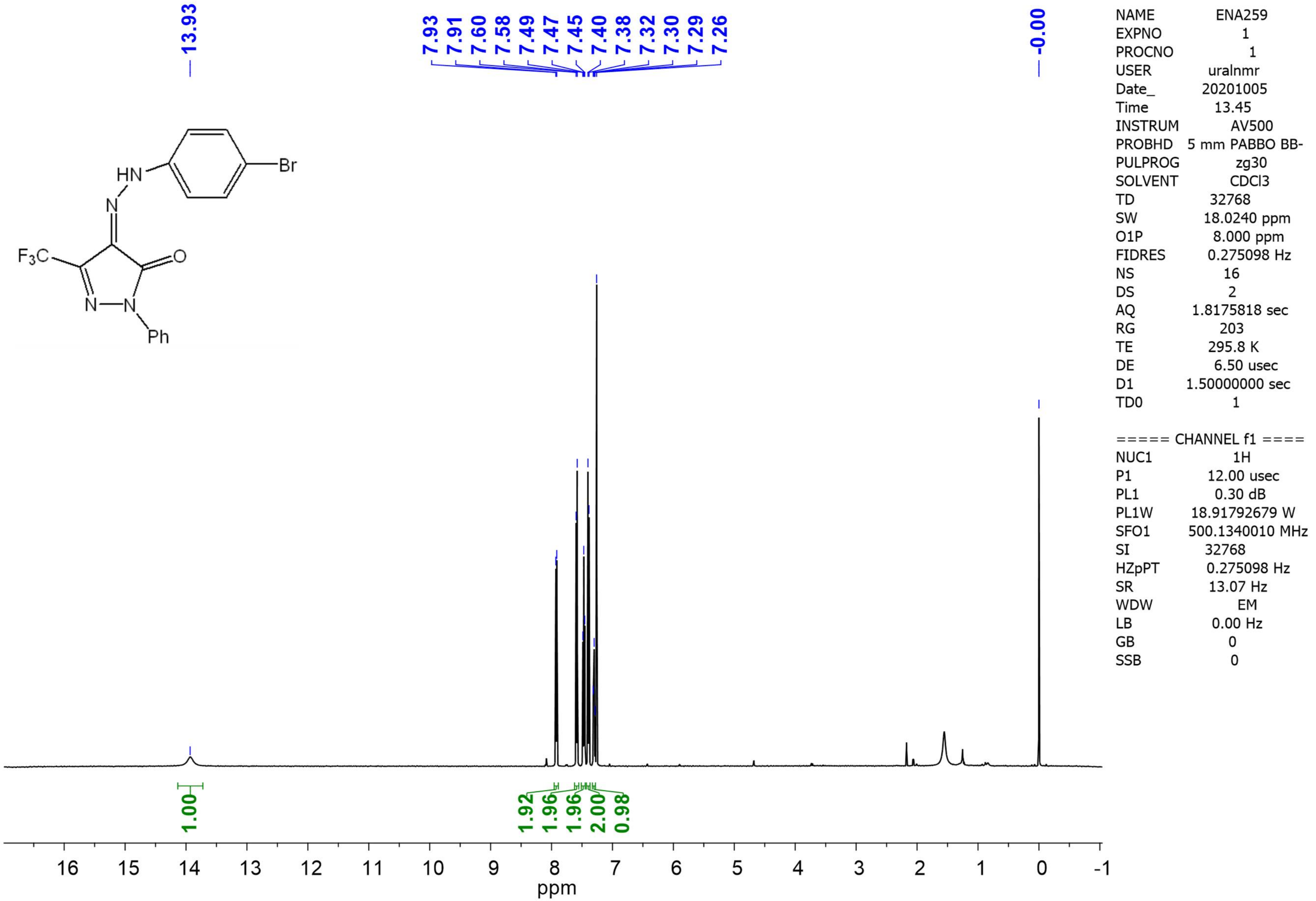

Figure S31. ^{13}C NMR spectrum of compound **5k**

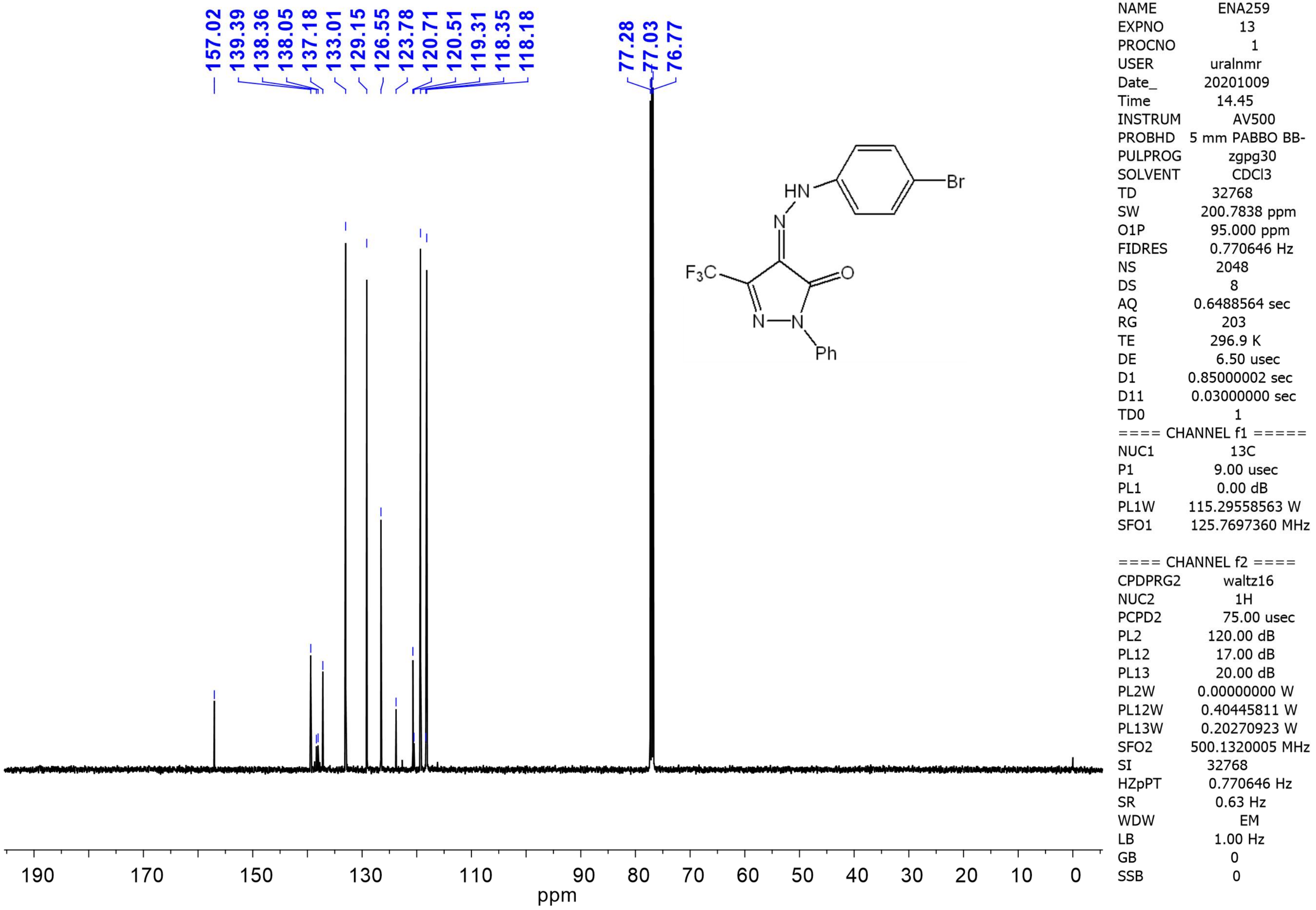

Figure S32. ^{19}F NMR spectrum of compound **5k**

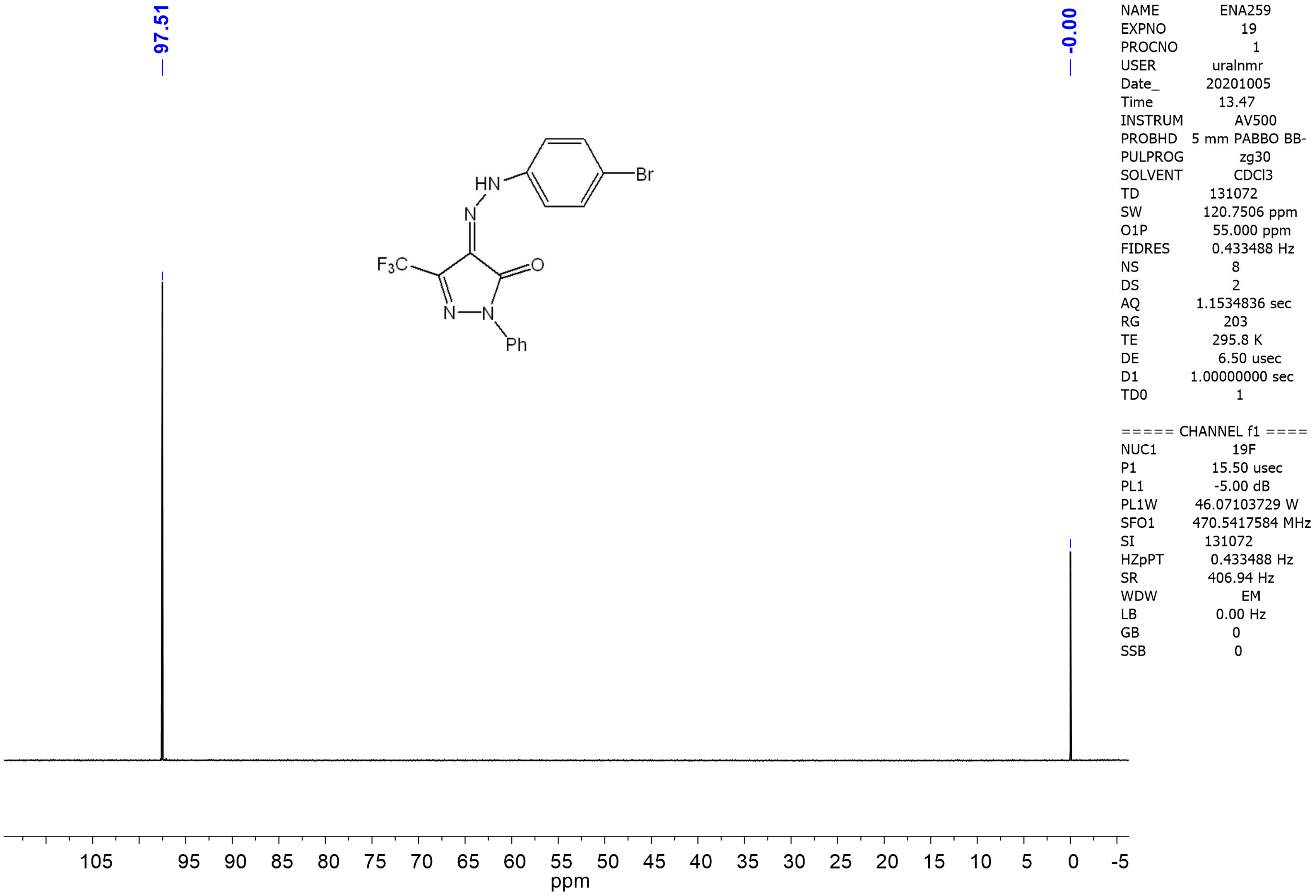

Figure S33. ^{1}H NMR spectrum of compound **5l**

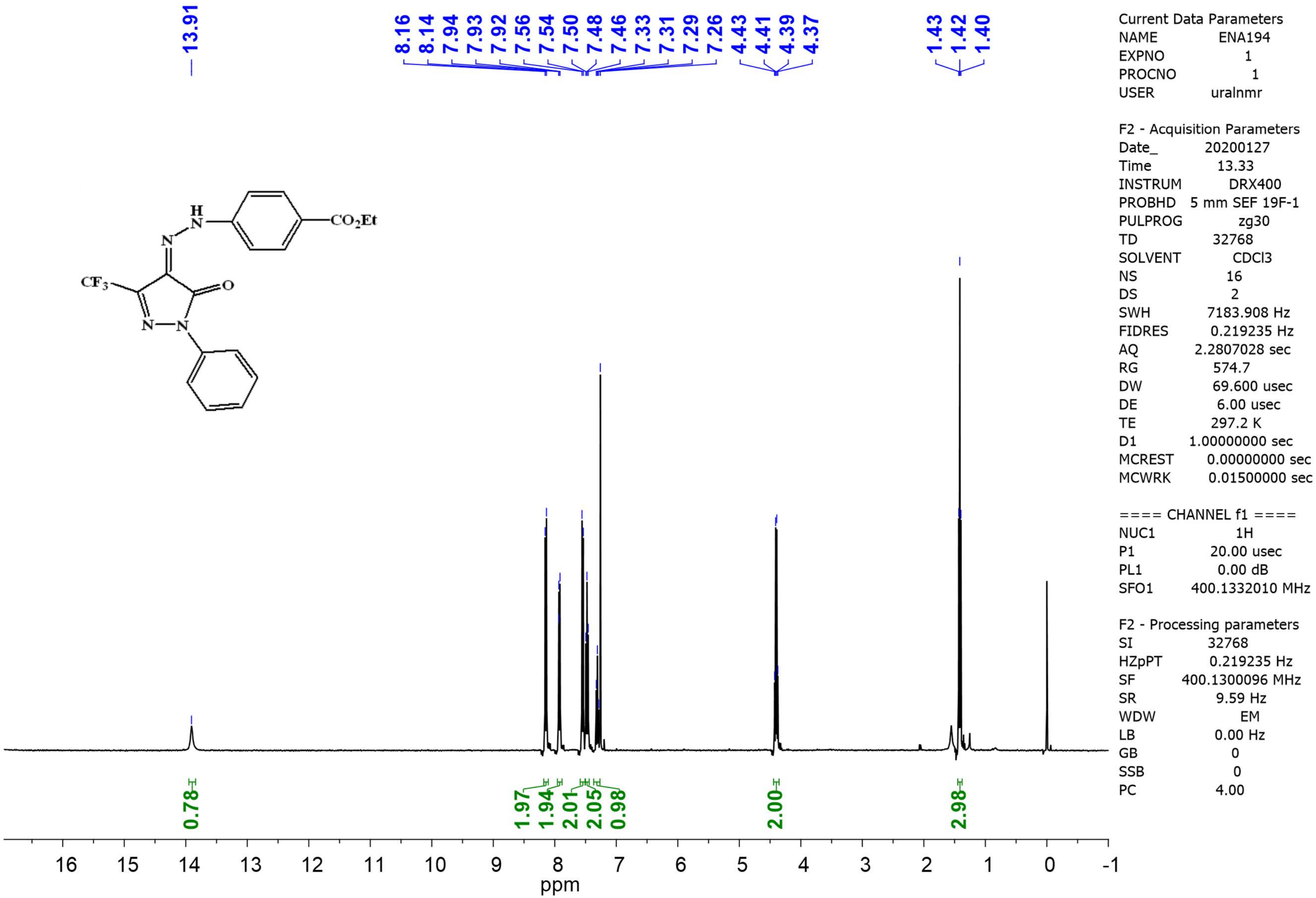

Figure S34. ^{13}C NMR spectrum of compound **5l**

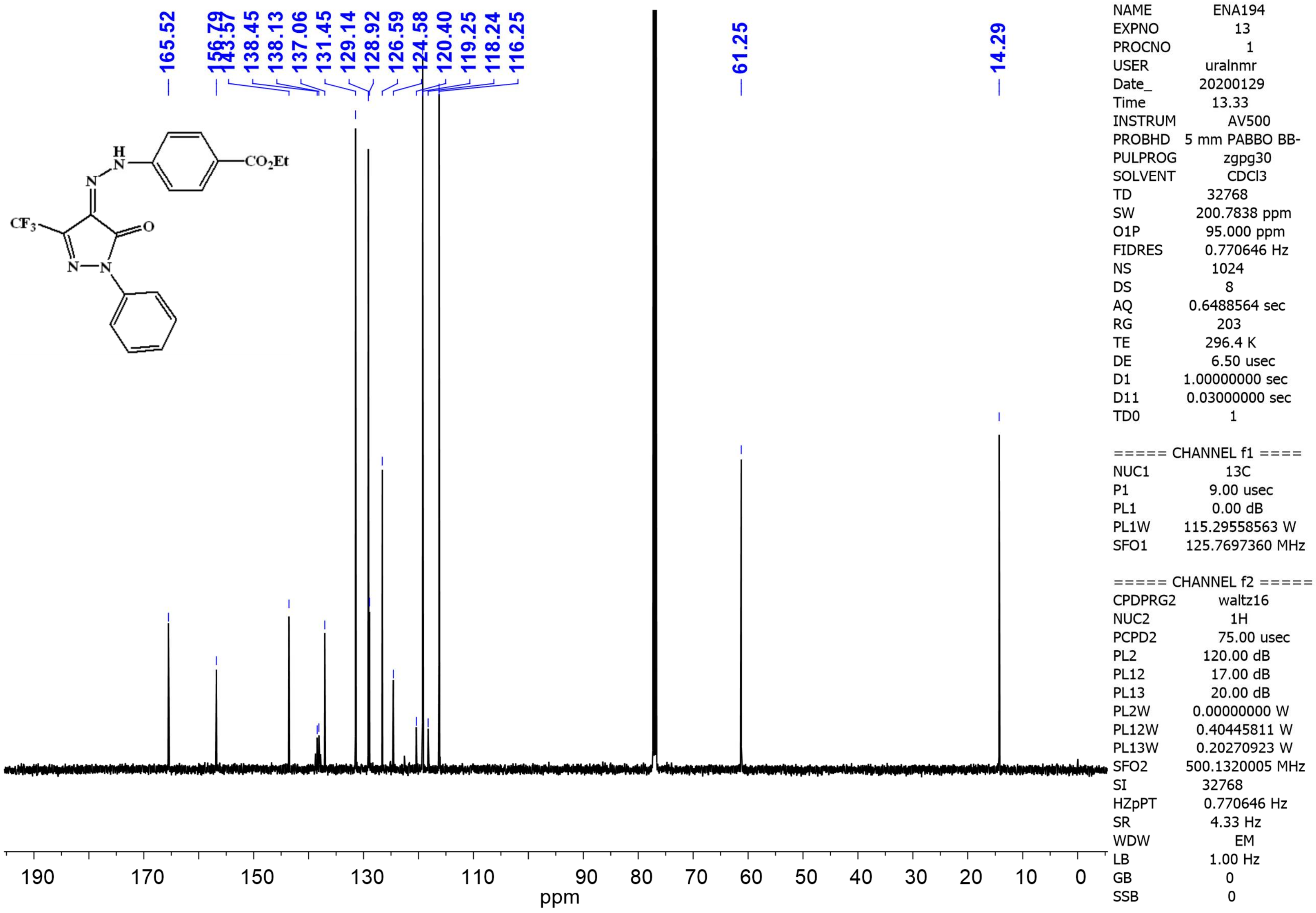

Figure S35. ^{19}F NMR spectrum of compound **5l**

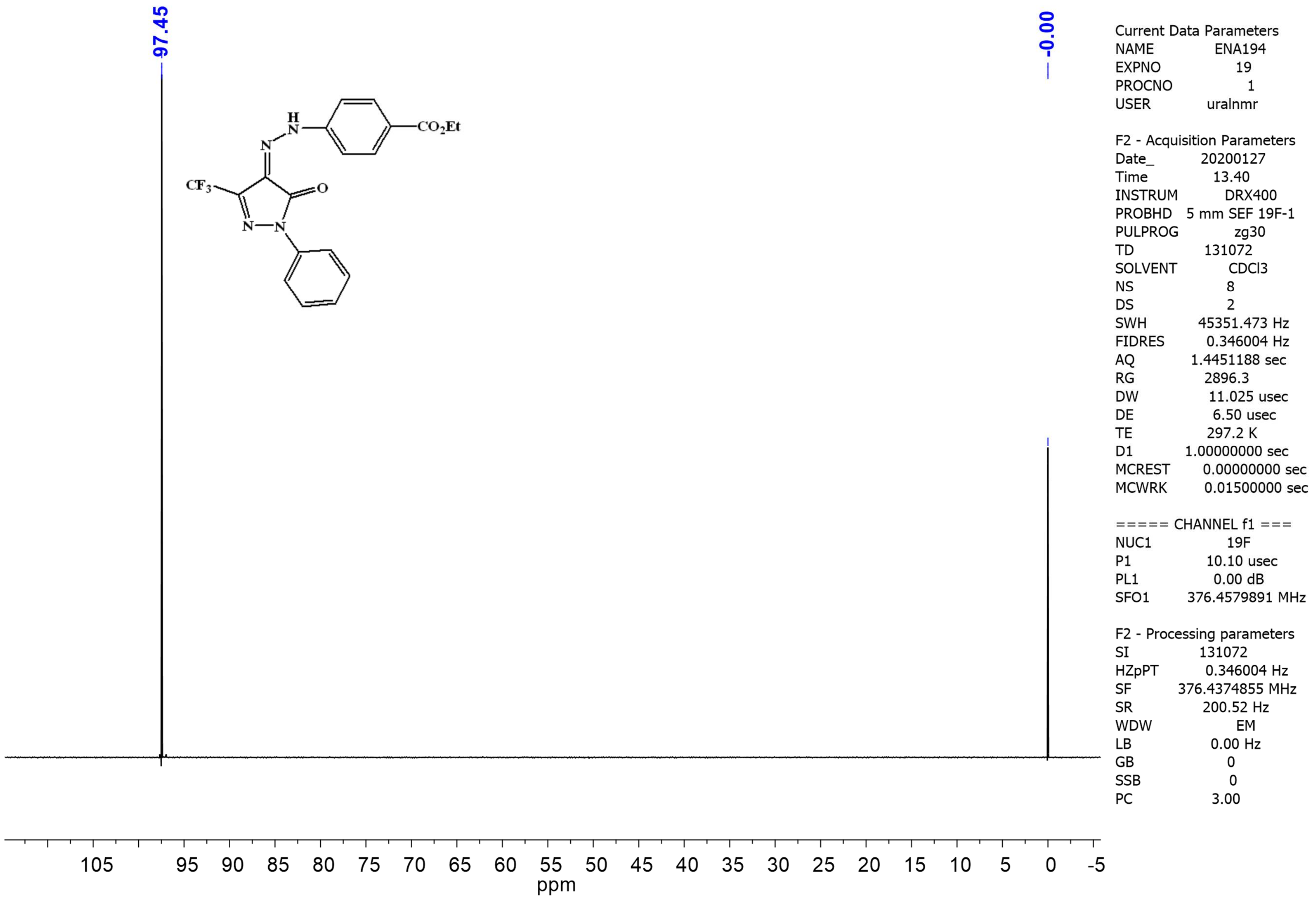

Figure S36. ^{1}H NMR spectrum of compound **5m**

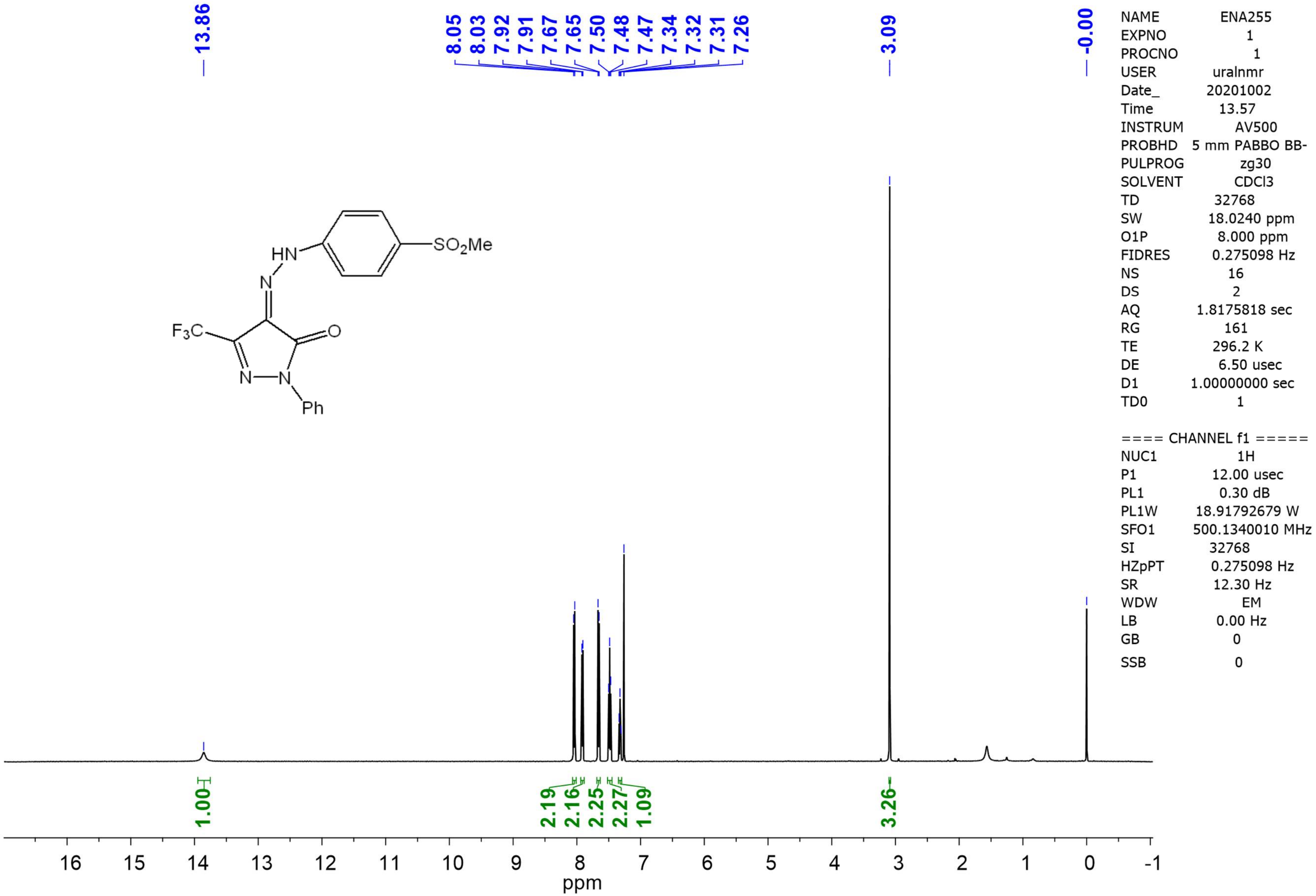

Figure S37. ^{13}C NMR spectrum of compound **5m**

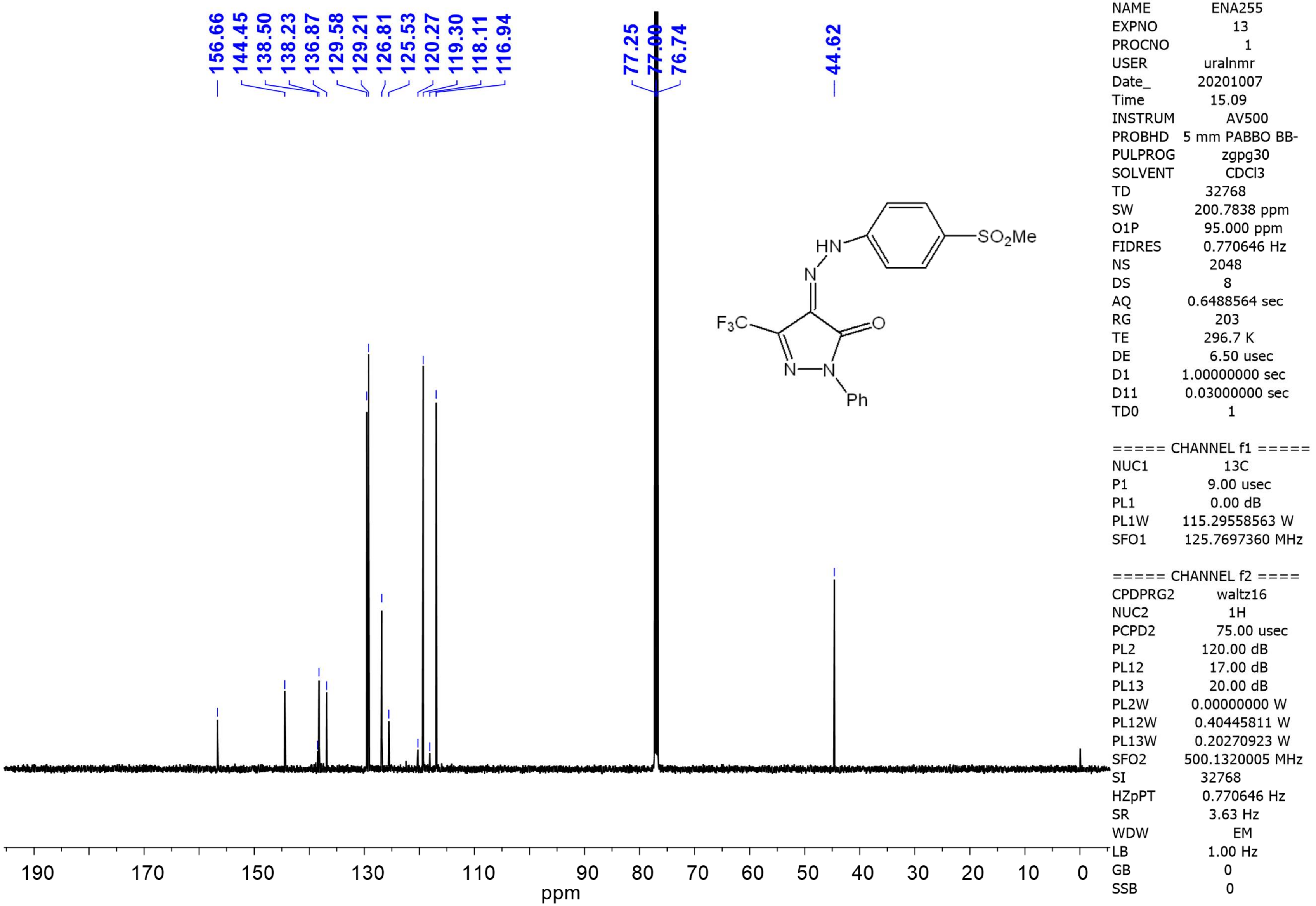

Figure S38. ^{19}F NMR spectrum of compound **5m**

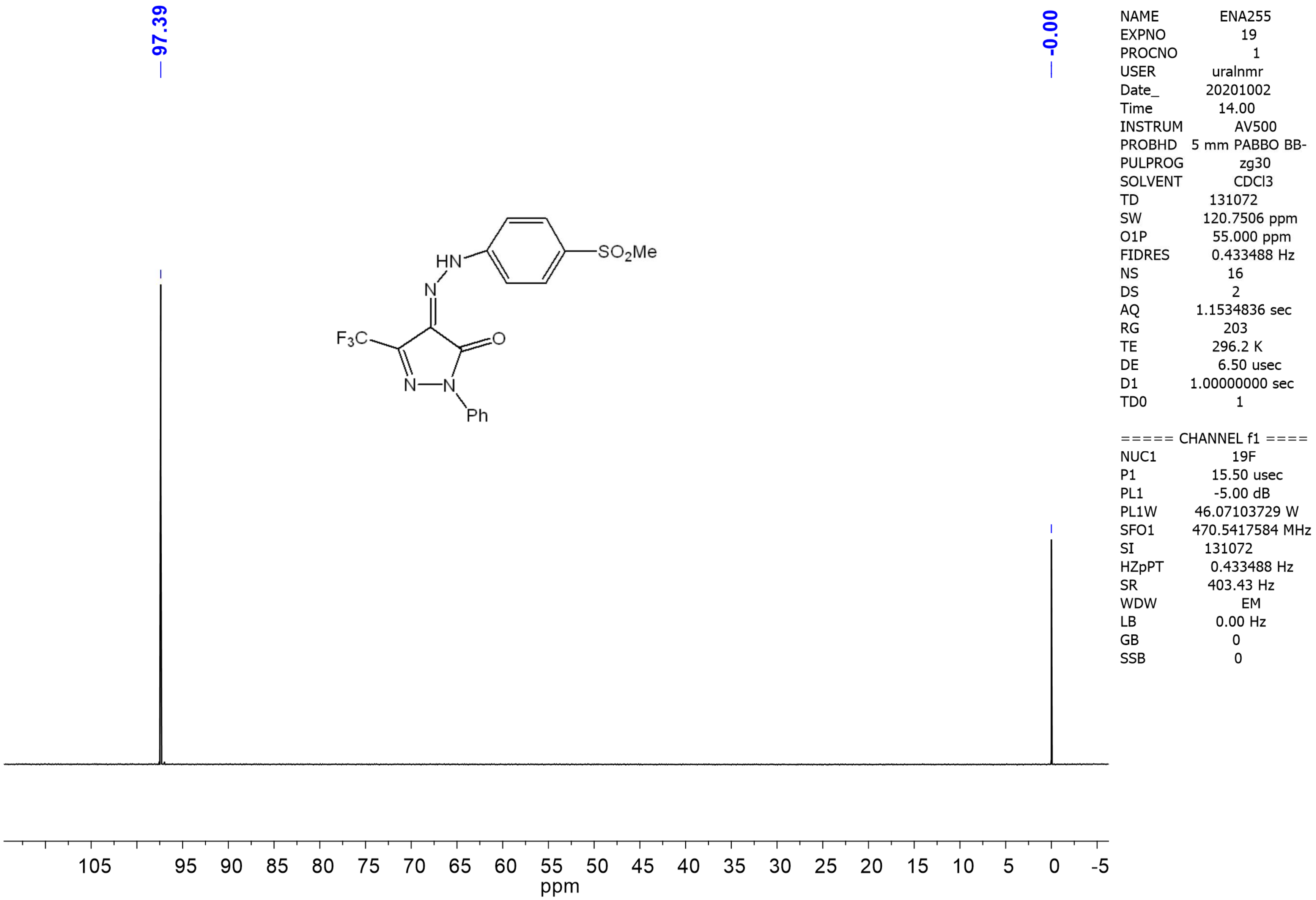

Figure S39. ^{1}H NMR spectrum of compound **5n**

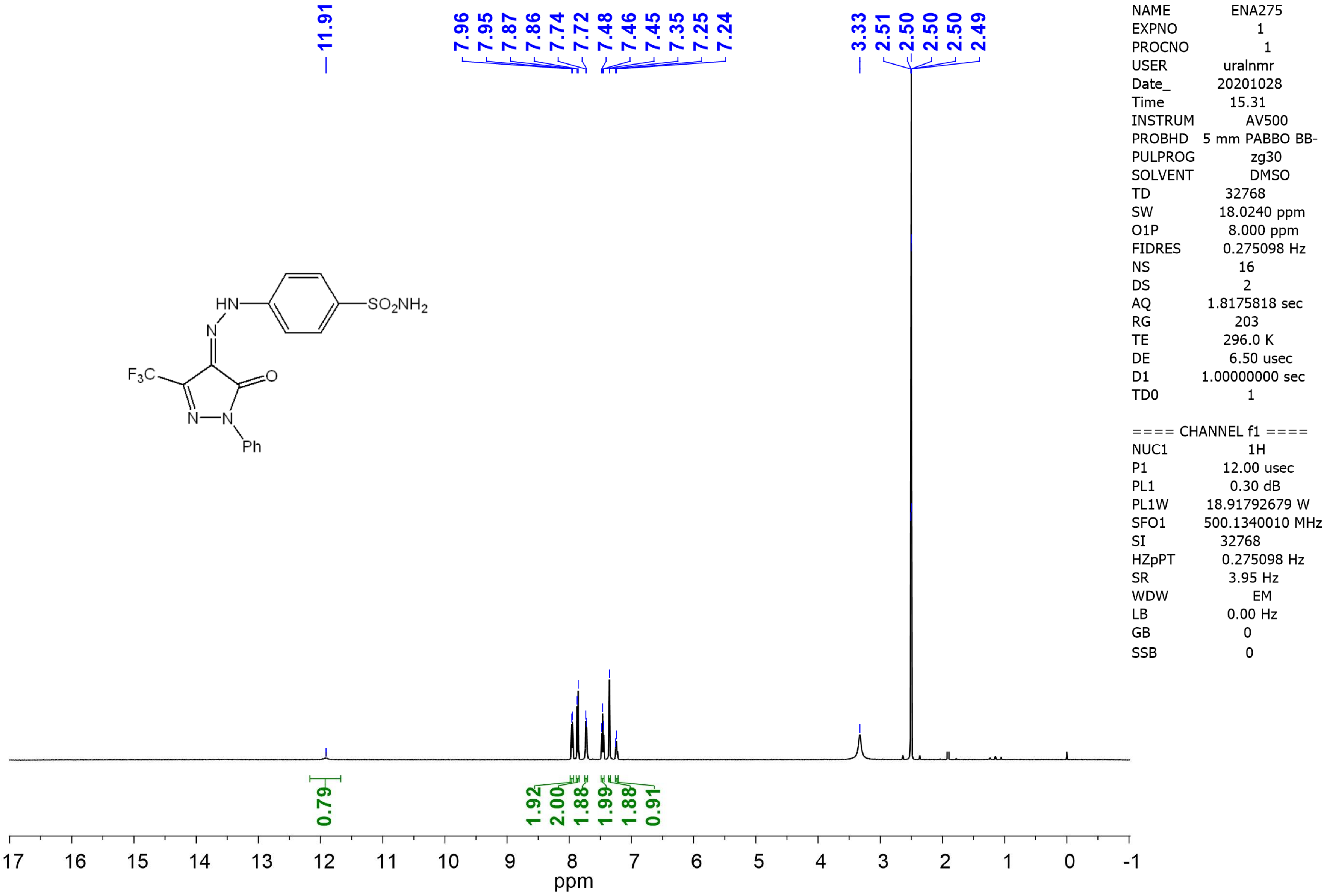

Figure S40. ^{13}C NMR spectrum of compound **5n**

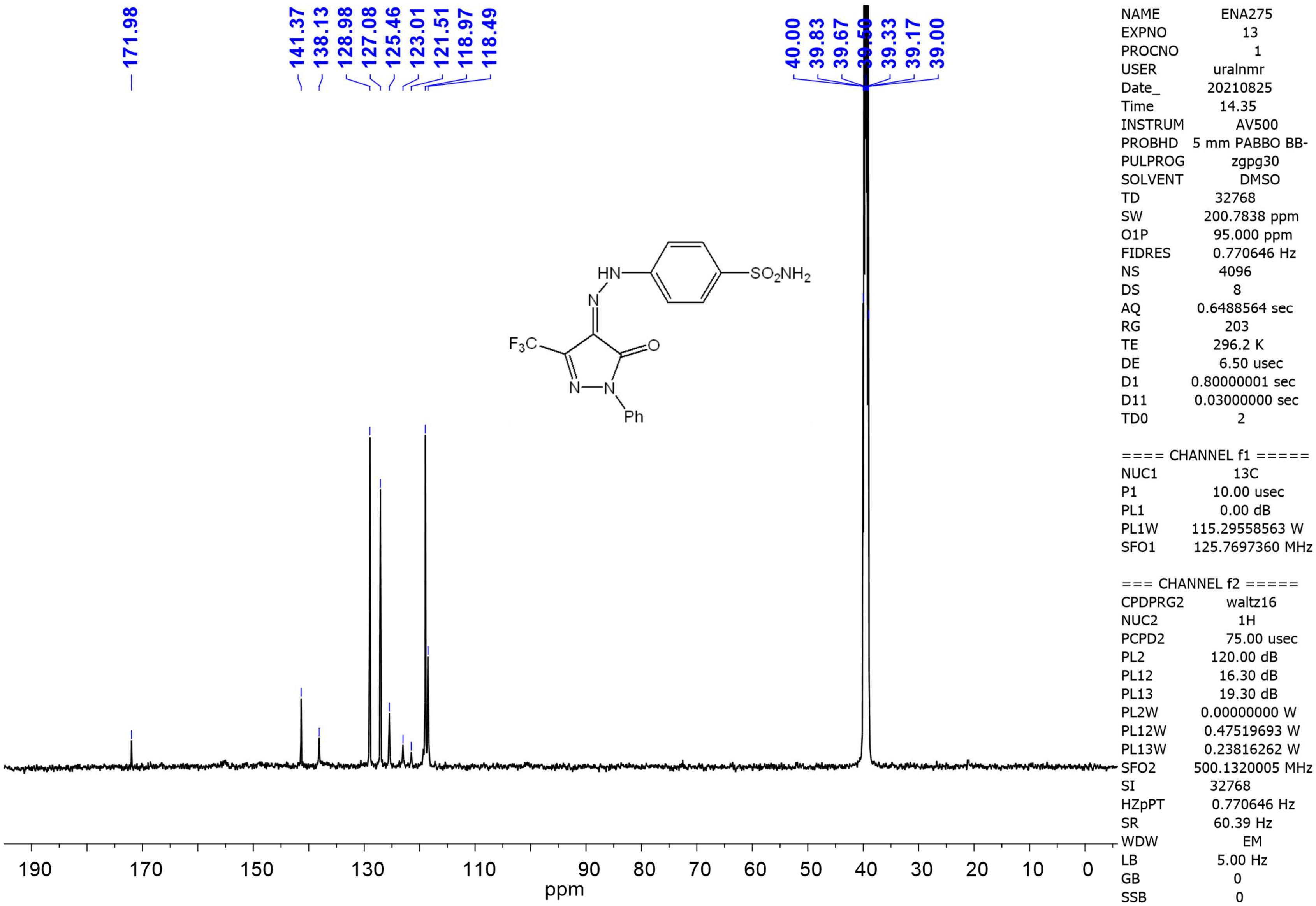

Figure S41. ^{19}F NMR spectrum of compound **5n**

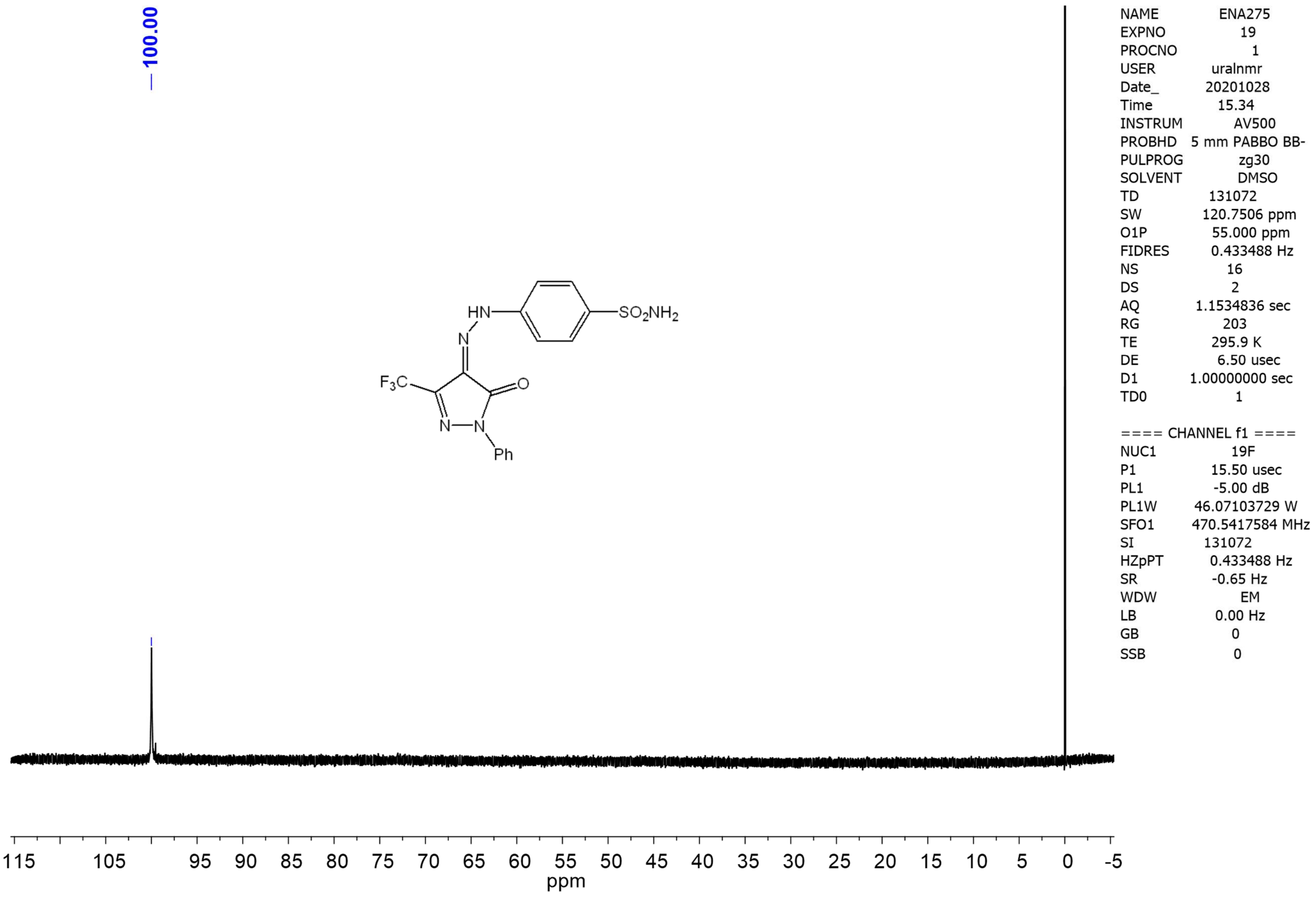

Figure S42. ^{1}H NMR spectrum of compound **5o**

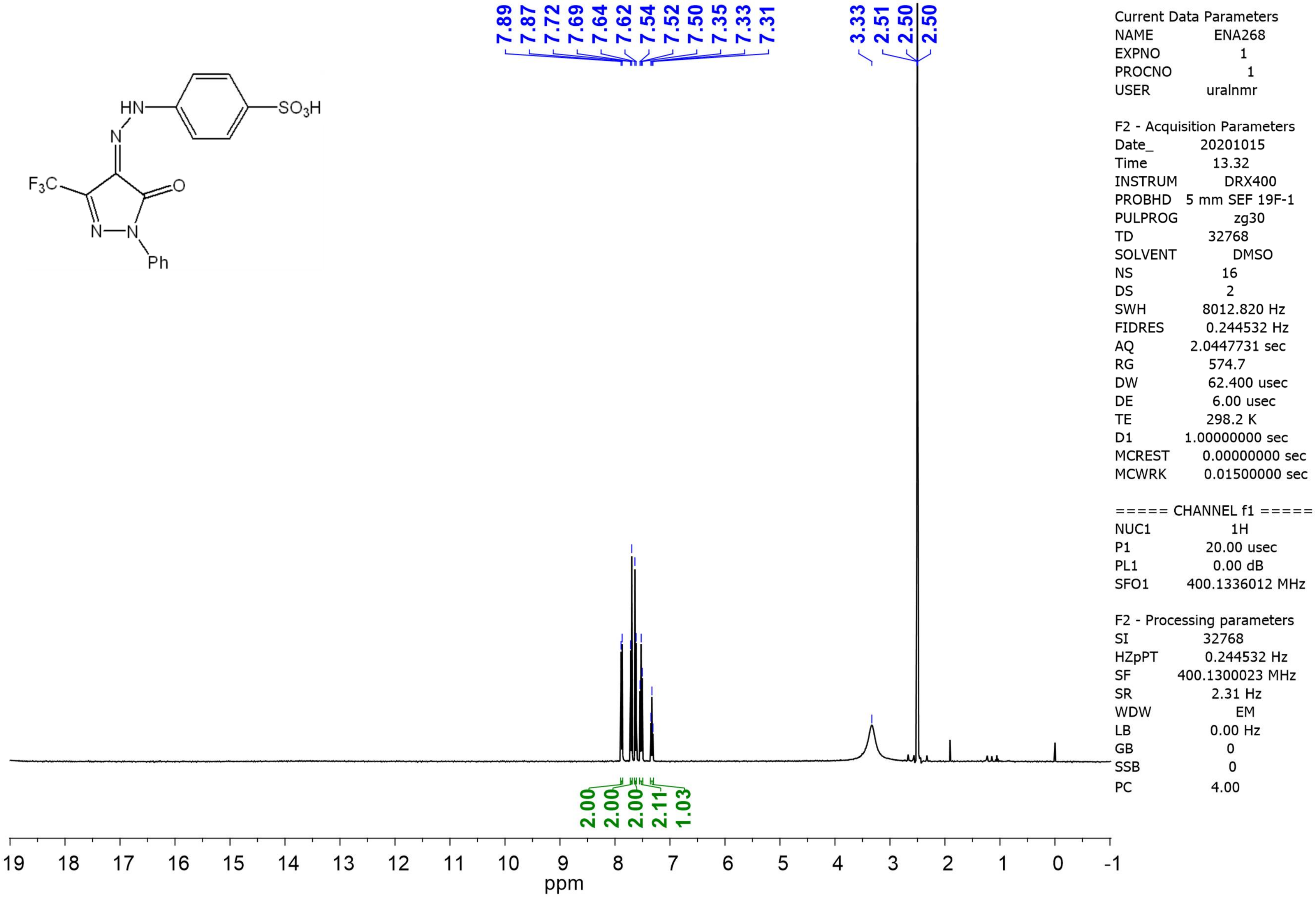

Current Data Parameters
NAME ENA268
EXPNO 1
PROCNO 1
USER uralnmr

F2 - Acquisition Parameters
Date_ 20201015
Time 13.32
INSTRUM DRX400
PROBHD 5 mm SEF 19F-1
PULPROG zg30
TD 32768
SOLVENT DMSO
NS 16
DS 2
SWH 8012.820 Hz
FIDRES 0.244532 Hz
AQ 2.0447731 sec
RG 574.7
DW 62.400 usec
DE 6.00 usec
TE 298.2 K
D1 1.00000000 sec
MCREST 0.00000000 sec
MCWRK 0.01500000 sec

===== CHANNEL f1 =====
NUC1 1H
P1 20.00 usec
PL1 0.00 dB
SFO1 400.1336012 MHz

F2 - Processing parameters
SI 32768
HZpPT 0.244532 Hz
SF 400.1300023 MHz
SR 2.31 Hz
WDW EM
LB 0.00 Hz
GB 0
SSB 0
PC 4.00

Figure S43. ^{13}C NMR spectrum of compound **5o**

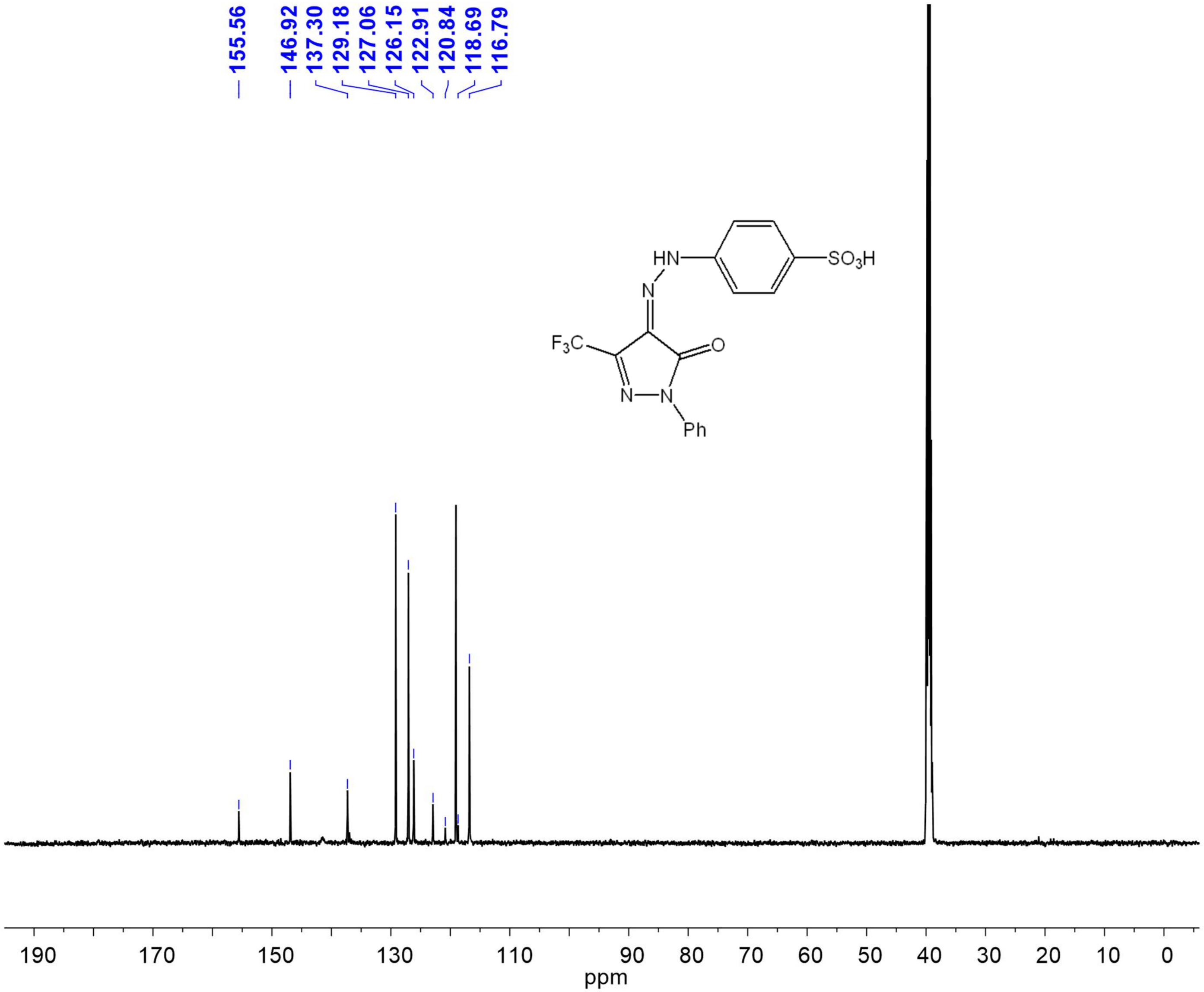

NAME ENA268
EXPNO 13
PROCNO 1
USER uralnmr
Date_ 20210824
Time 10.44
INSTRUM AV500
PROBHD 5 mm PABBO BB-
PULPROG zgpg30
SOLVENT DMSO
TD 32768
SW 200.7838 ppm
O1P 95.000 ppm
FIDRES 0.770646 Hz
NS 3072
DS 8
AQ 0.6488564 sec
RG 203
TE 297.0 K
DE 6.50 usec
D1 0.85000002 sec
D11 0.03000000 sec
TD0 1

==== CHANNEL f1 =====
NUC1 13C
P1 10.00 usec
PL1 0.00 dB
PL1W 115.29558563 W
SFO1 125.7697360 MHz

===== CHANNEL f2 =====
CPDPRG2 waltz16
NUC2 1H
PCPD2 75.00 usec
PL2 120.00 dB
PL12 16.30 dB
PL13 19.30 dB
PL2W 0.00000000 W
PL12W 0.47519693 W
PL13W 0.23816262 W
SFO2 500.1320005 MHz
SI 32768
HZpPT 0.770646 Hz
SR 61.18 Hz
WDW EM
LB 2.00 Hz
GB 0
SSB 0

Figure S44. ^{19}F NMR spectrum of compound **5o**

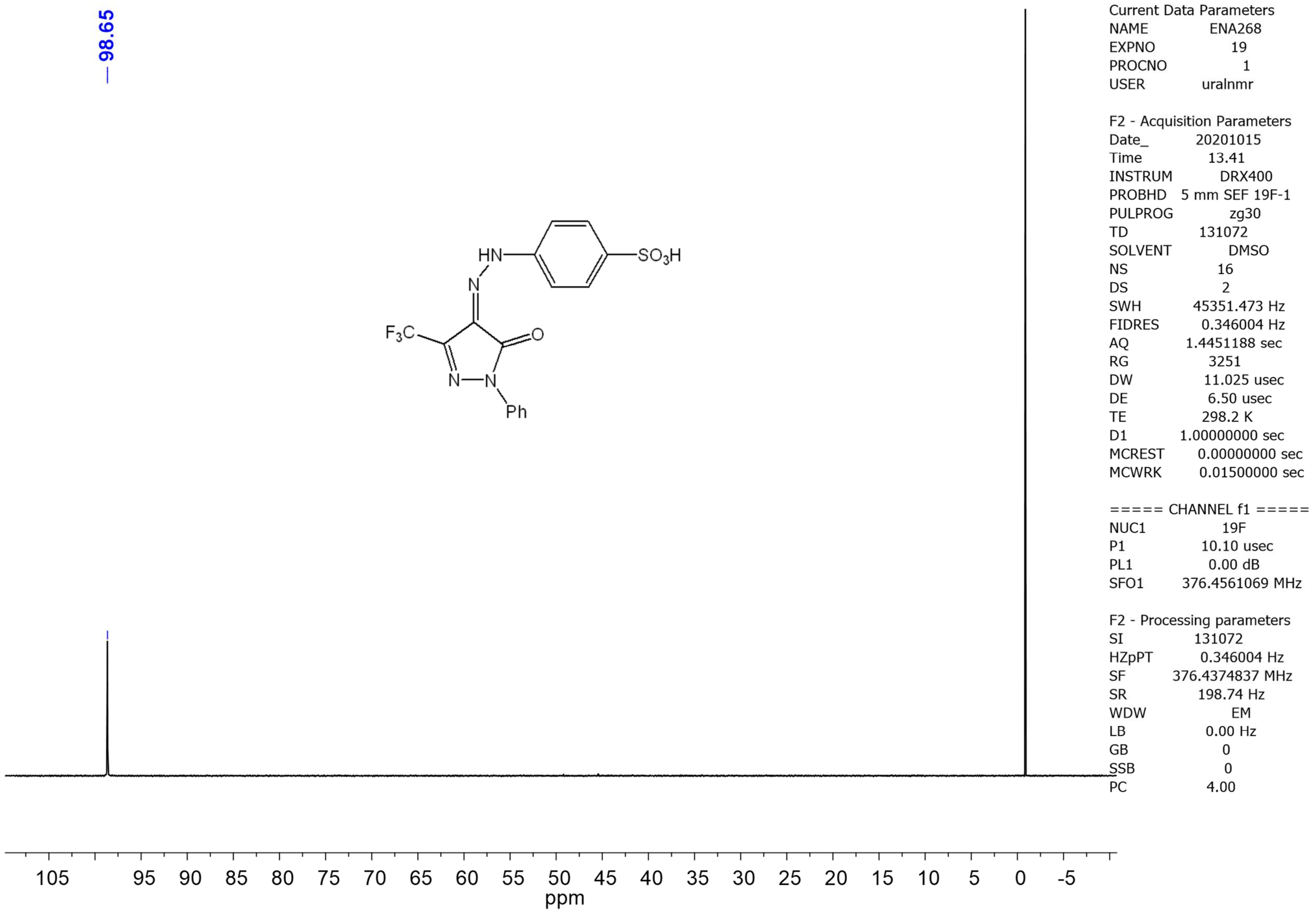

Figure S45. ^{1}H NMR spectrum of compound **5p**

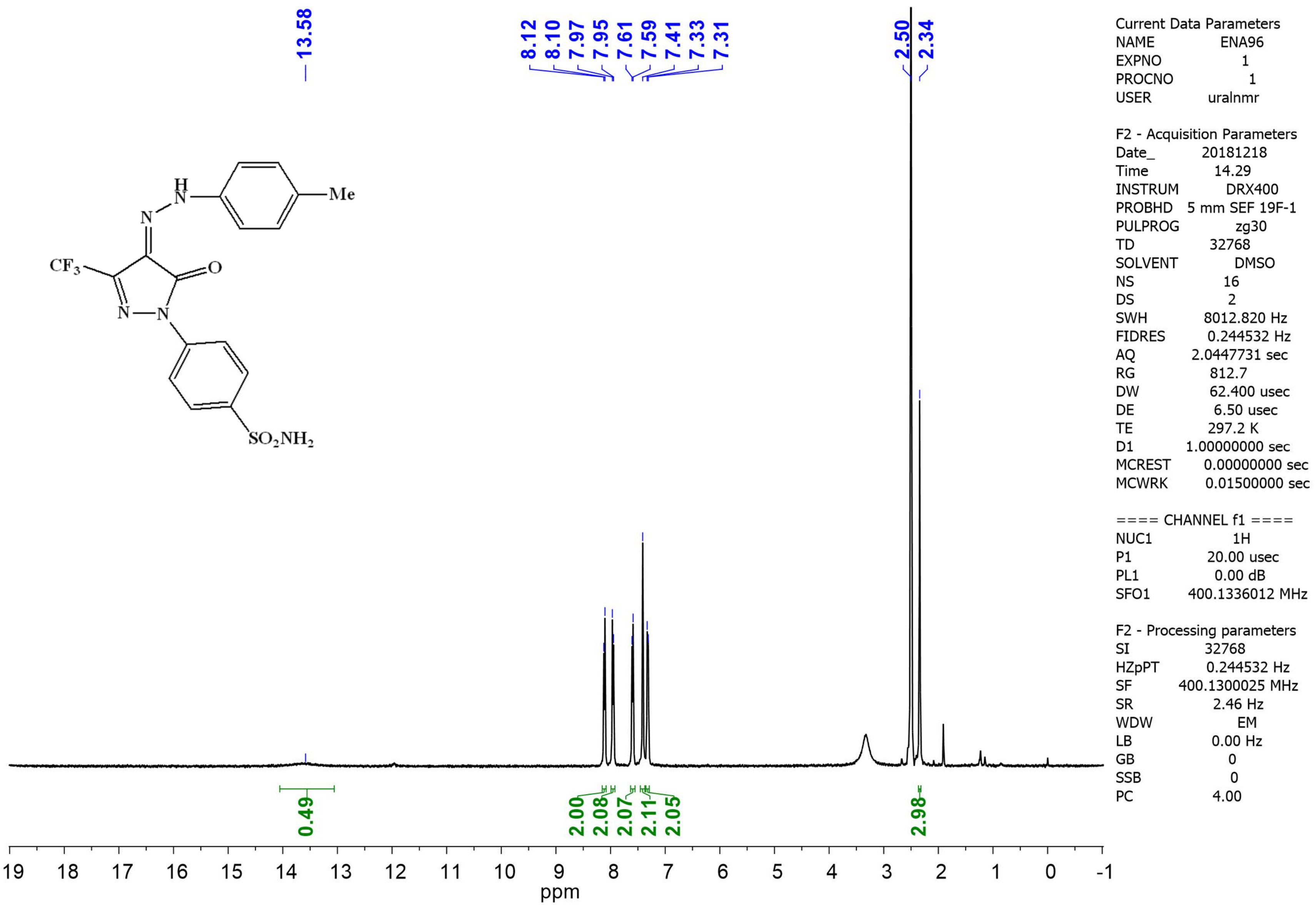

Figure S46. ^{13}C NMR spectrum of compound **5p**

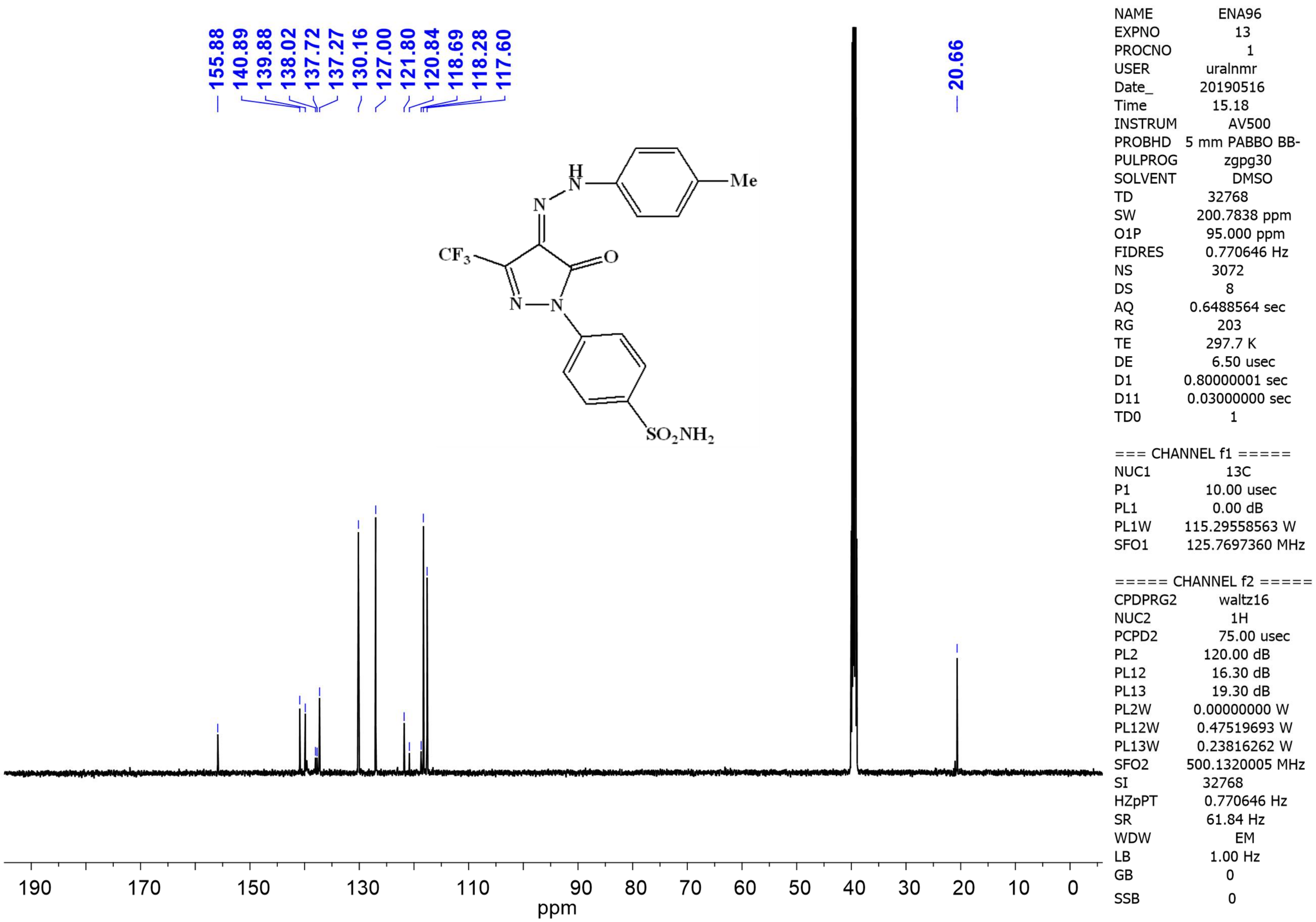

Figure S47. ^{19}F NMR spectrum of compound **5p**

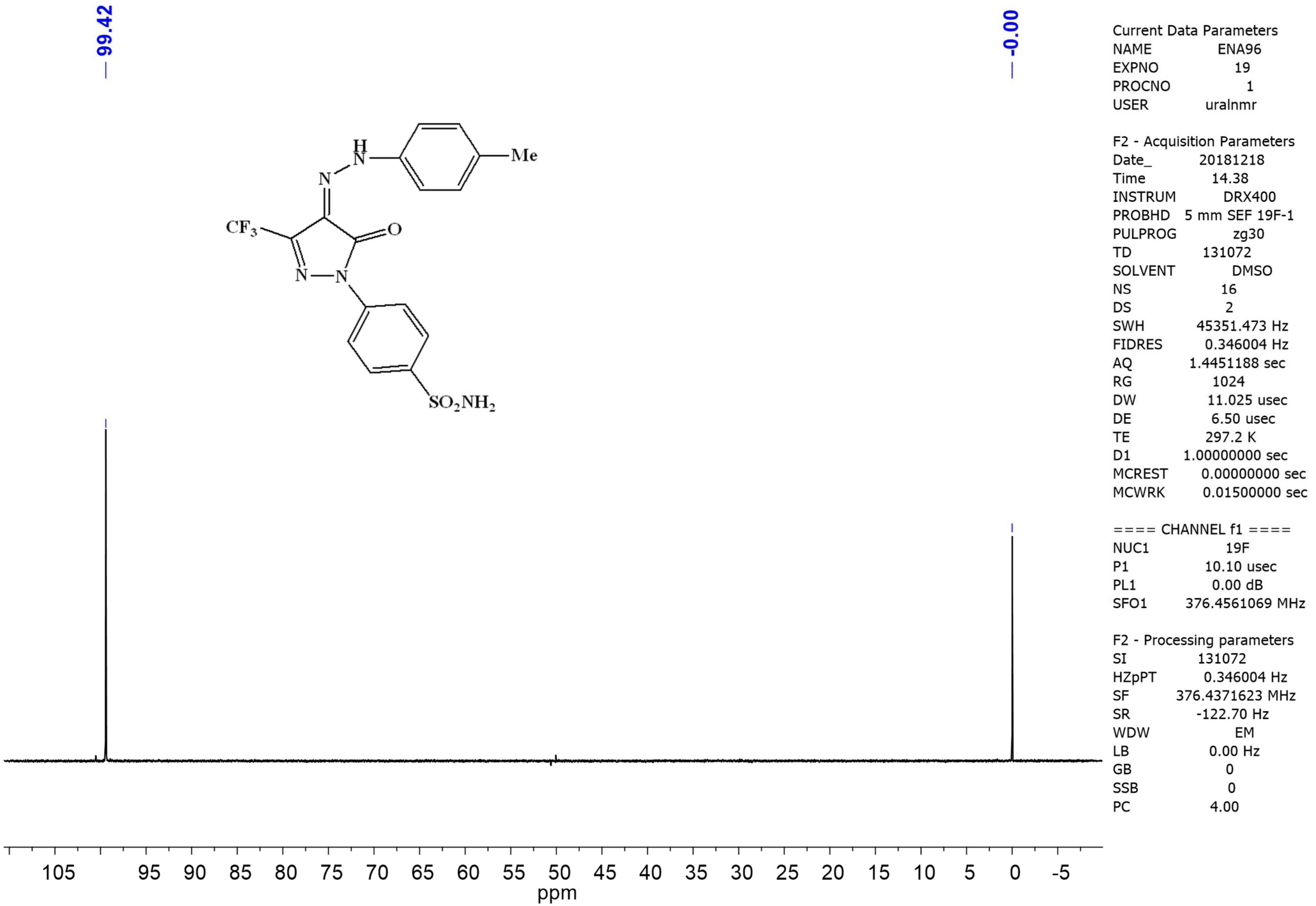

Figure S48. ^{1}H NMR spectrum of compound **5q**

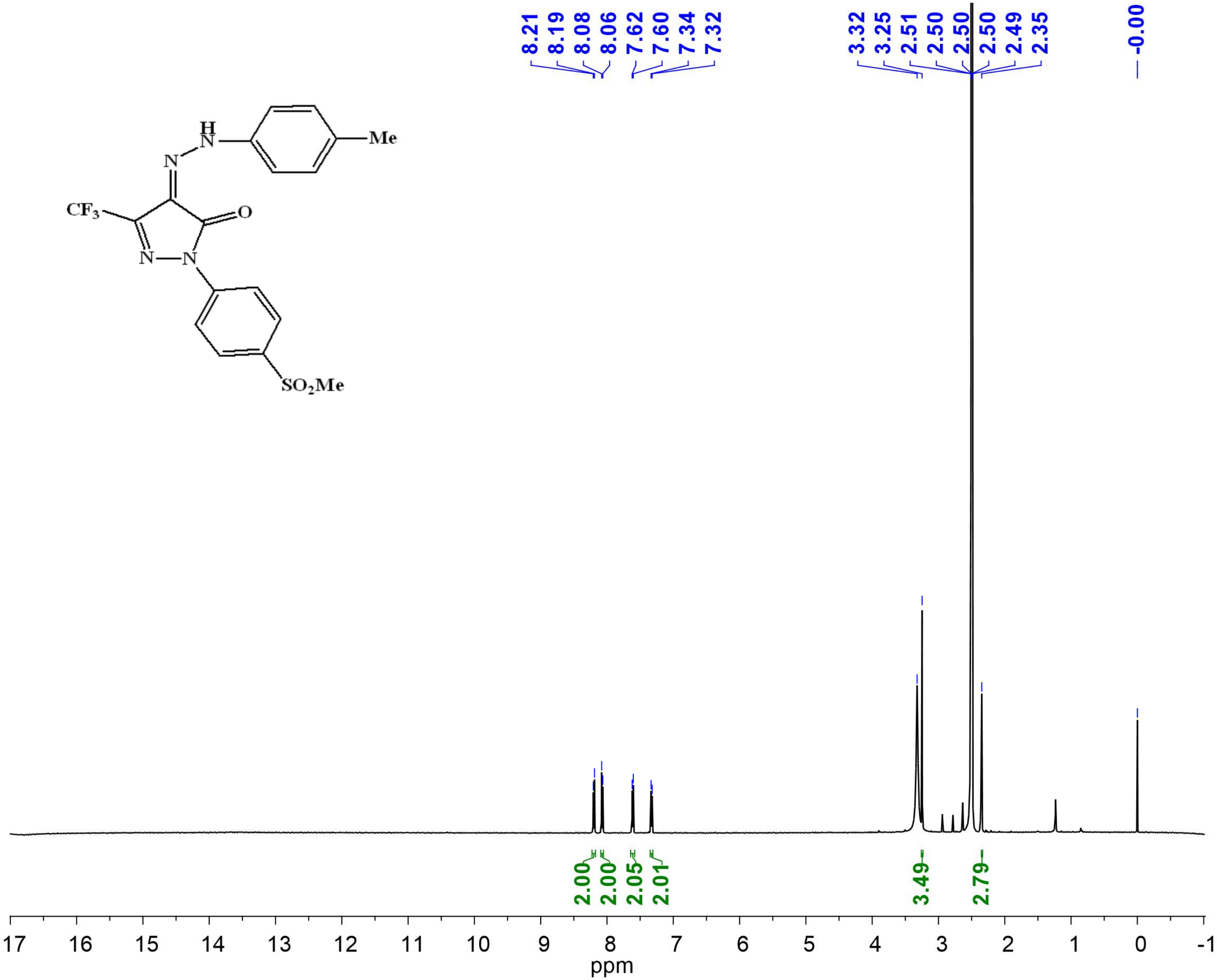

NAME ENA200a
EXPNO 1
PROCNO 1
USER uralnmr
Date_ 20200211
Time 12.47
INSTRUM AV500
PROBHD 5 mm PABBO BB-
PULPROG zg30
SOLVENT DMSO
TD 32768
SW 18.0240 ppm
O1P 8.000 ppm
FIDRES 0.275098 Hz
NS 16
DS 2
AQ 1.8175818 sec
RG 114
TE 295.9 K
DE 6.50 usec
D1 1.00000000 sec
TD0 1

===== CHANNEL f1 ====
NUC1 1H
P1 12.00 usec
PL1 0.30 dB
PL1W 18.91792679 W
SFO1 500.1340010 MHz
SI 32768
HZpPT 0.275098 Hz
SR 4.91 Hz
WDW EM
LB 0.00 Hz
GB 0
SSB 0

Figure S49. ^{19}F NMR spectrum of compound **5q**

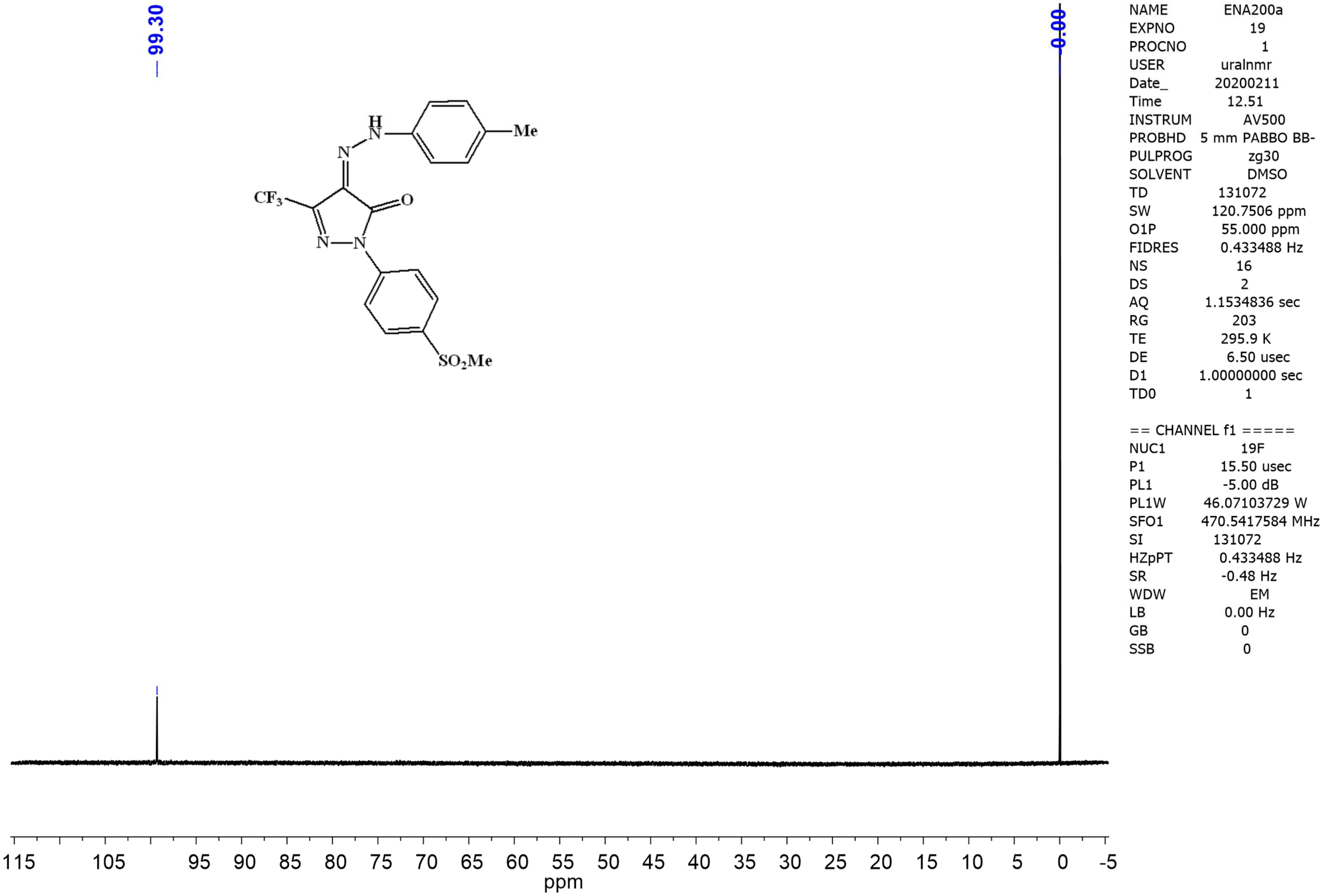

Figure S50. ^{1}H NMR spectrum of compound **5r**

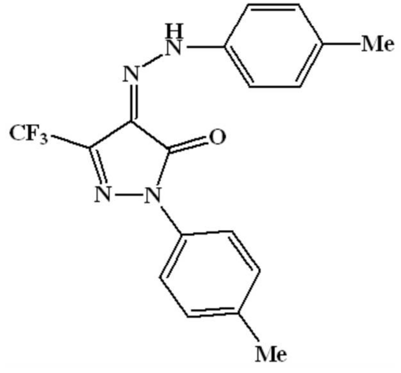

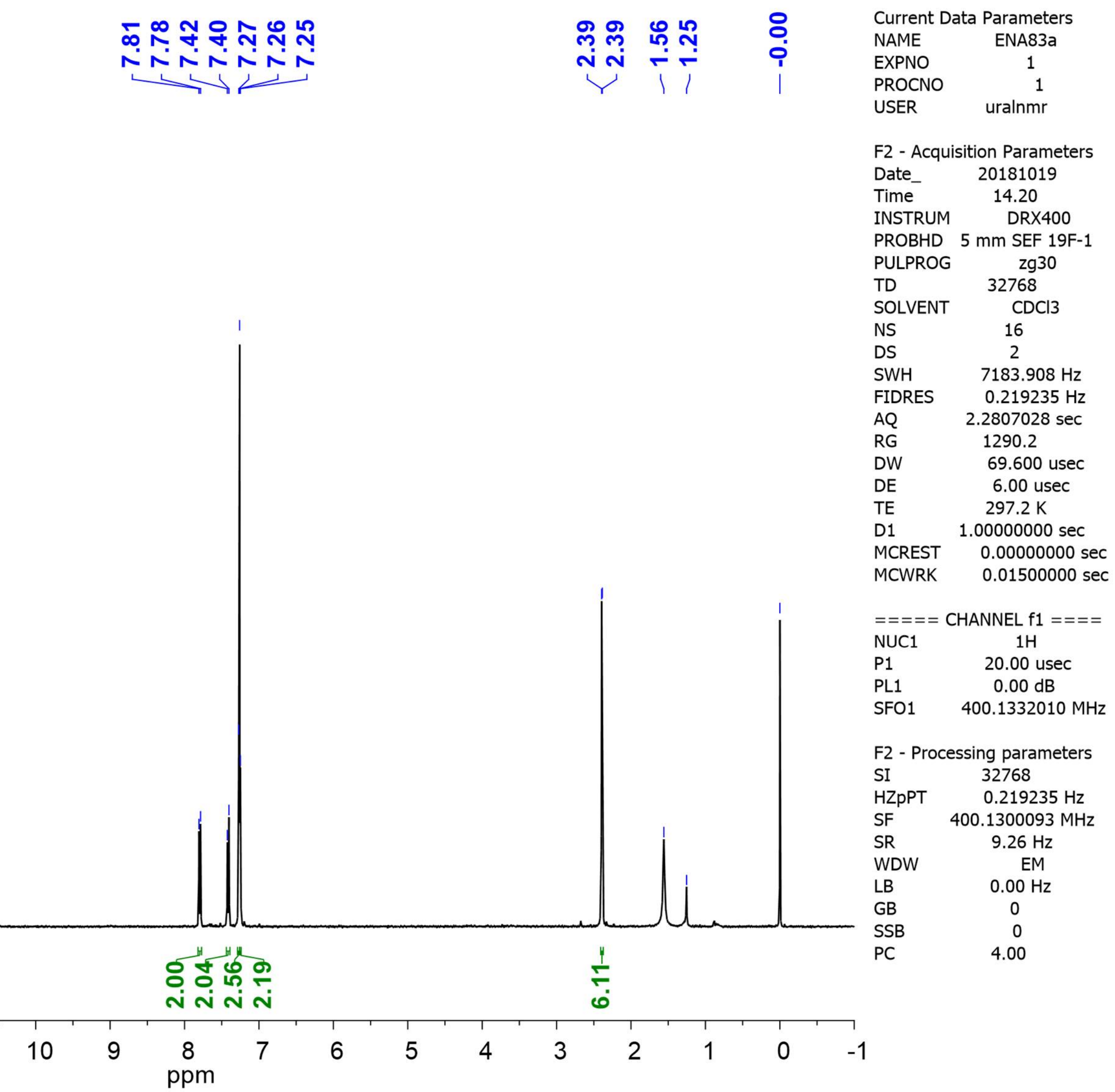

Figure S51. ^{13}C NMR spectrum of compound **5r**

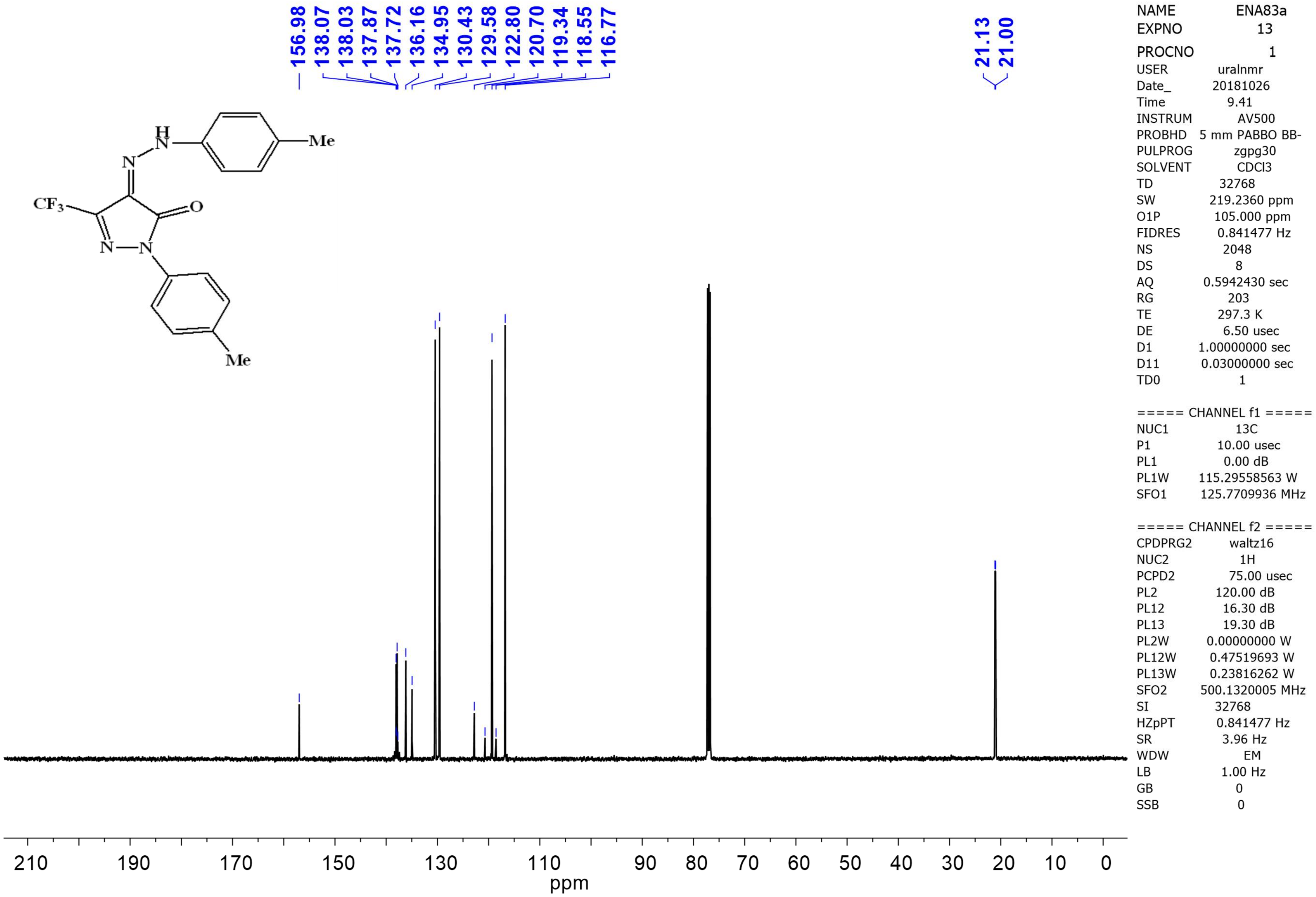

Figure S52. ^{19}F NMR spectrum of compound **5r**

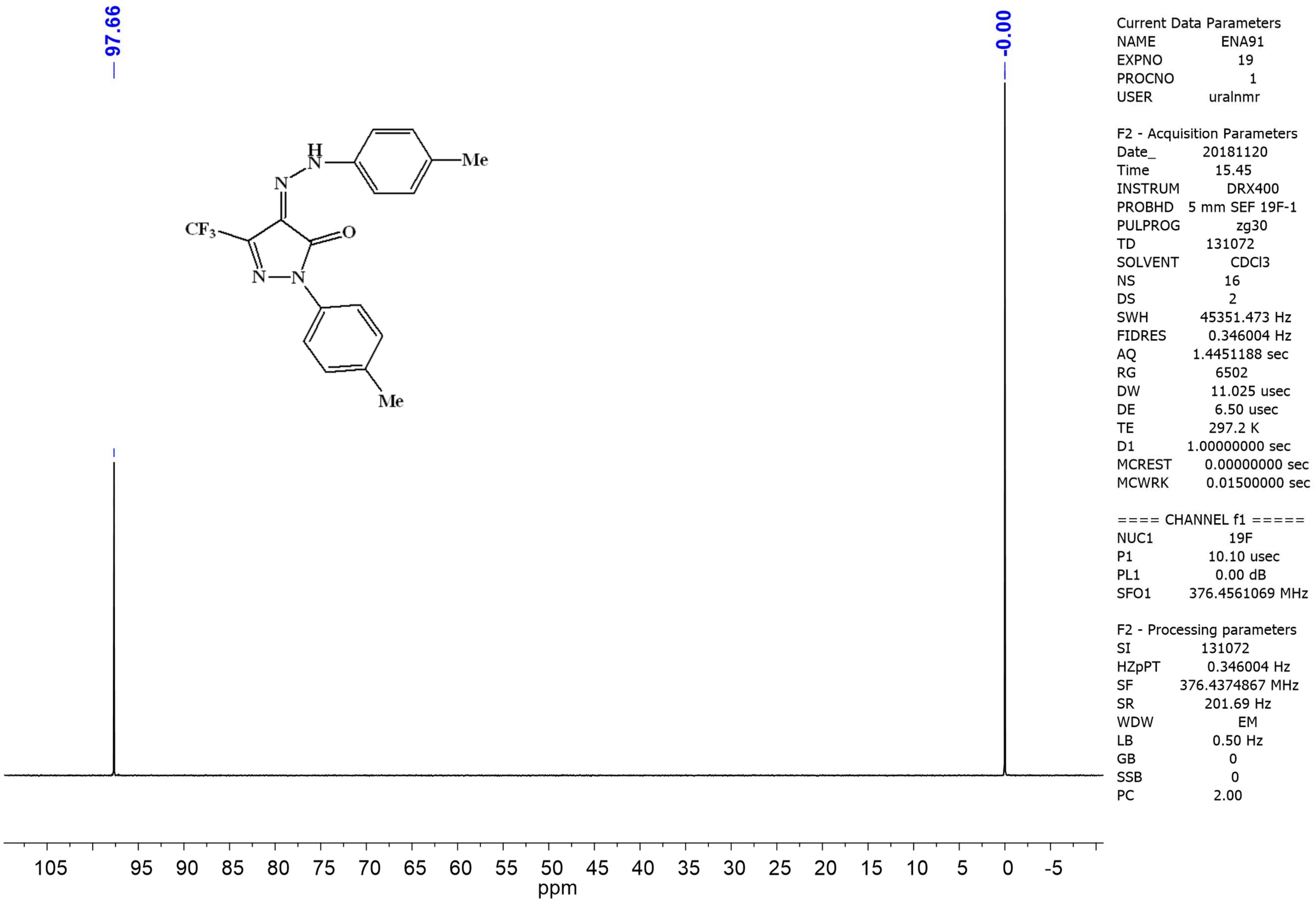

Figure S53. ^{1}H NMR spectrum of compound **6a**

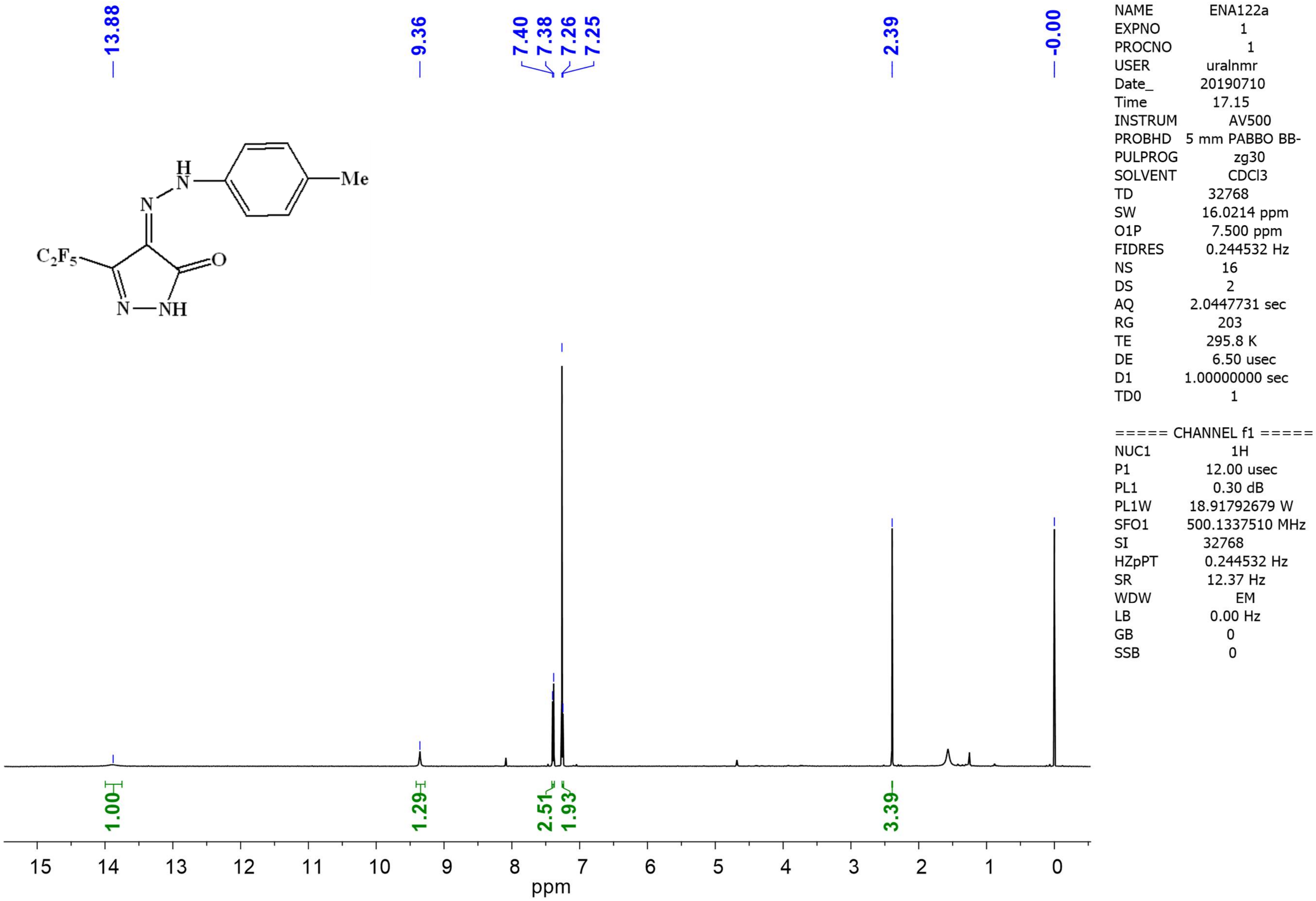

Figure S54. ^{13}C NMR spectrum of compound **6a**

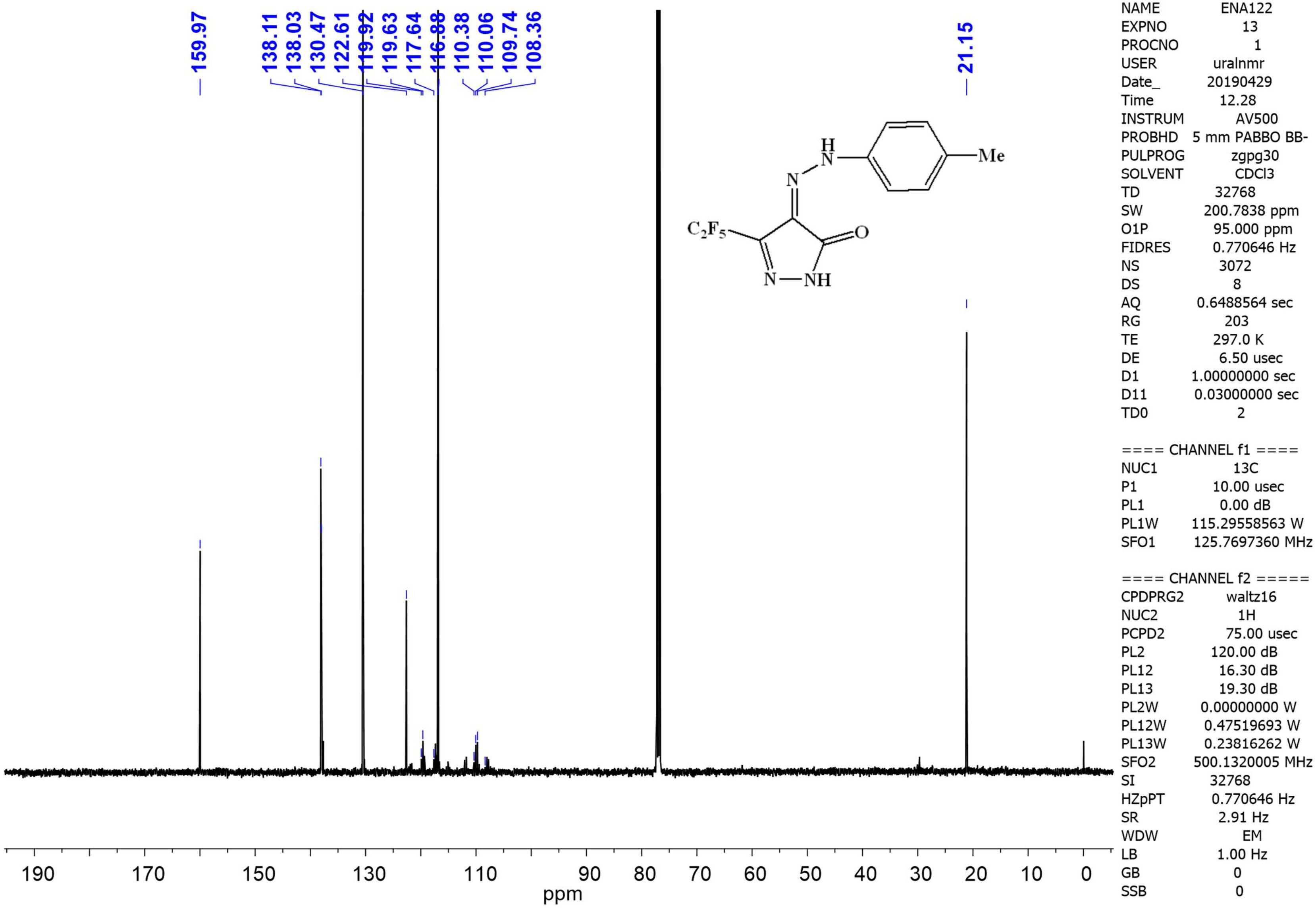

Figure S55. ^{19}F NMR spectrum of compound **6a**

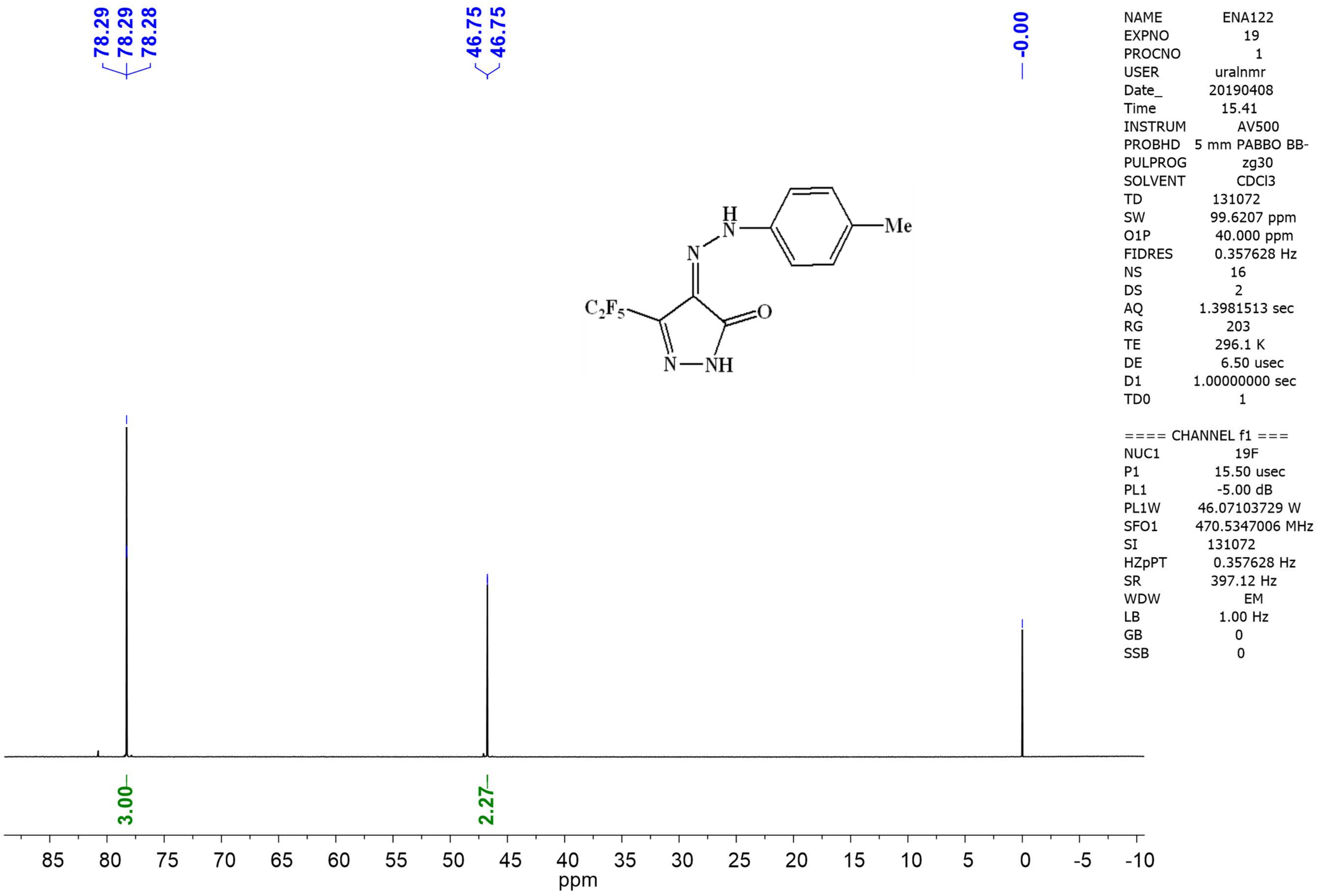

Figure S56. ^{1}H NMR spectrum of compound **6b**

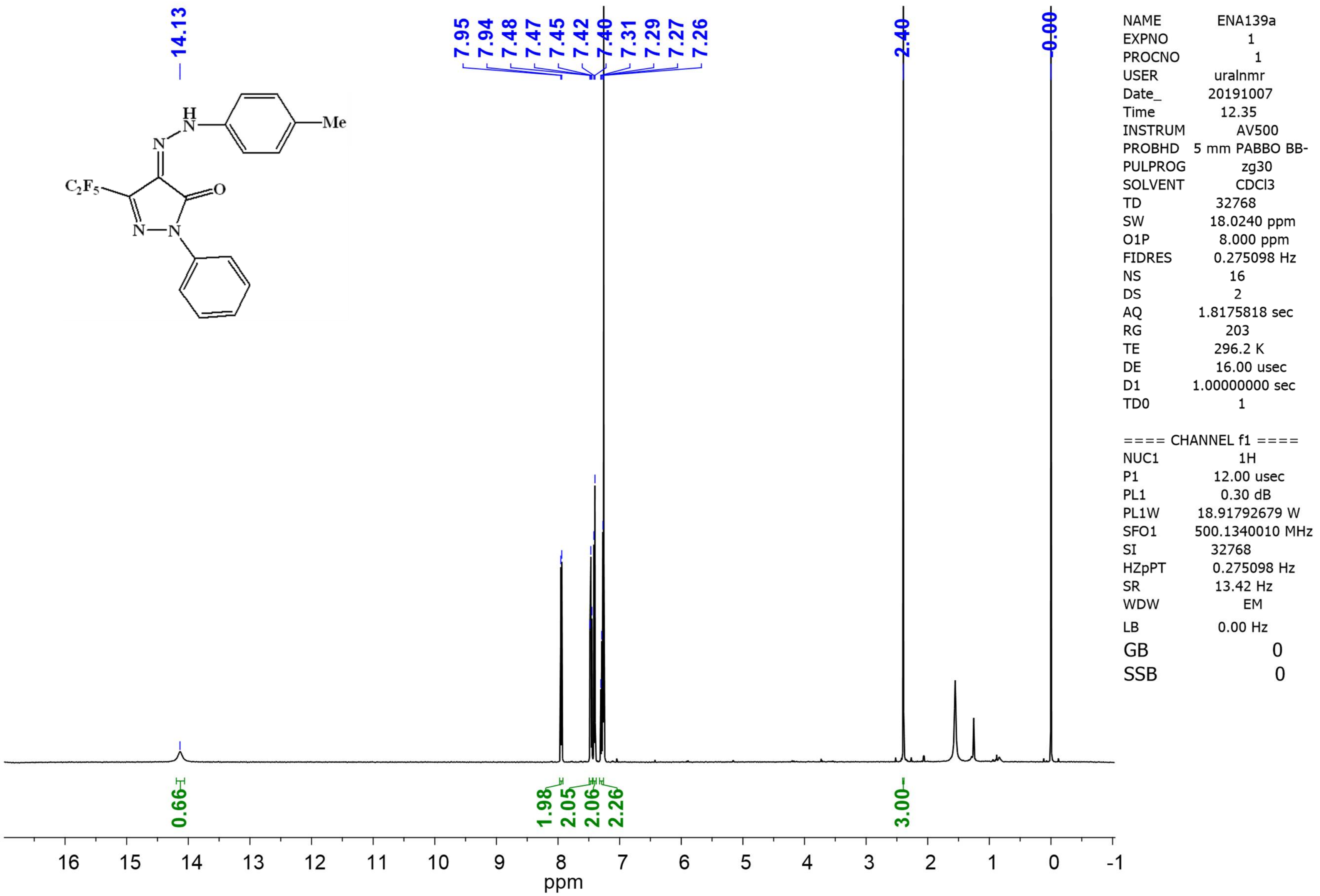

Figure S57. ^{13}C NMR spectrum of compound **6b**

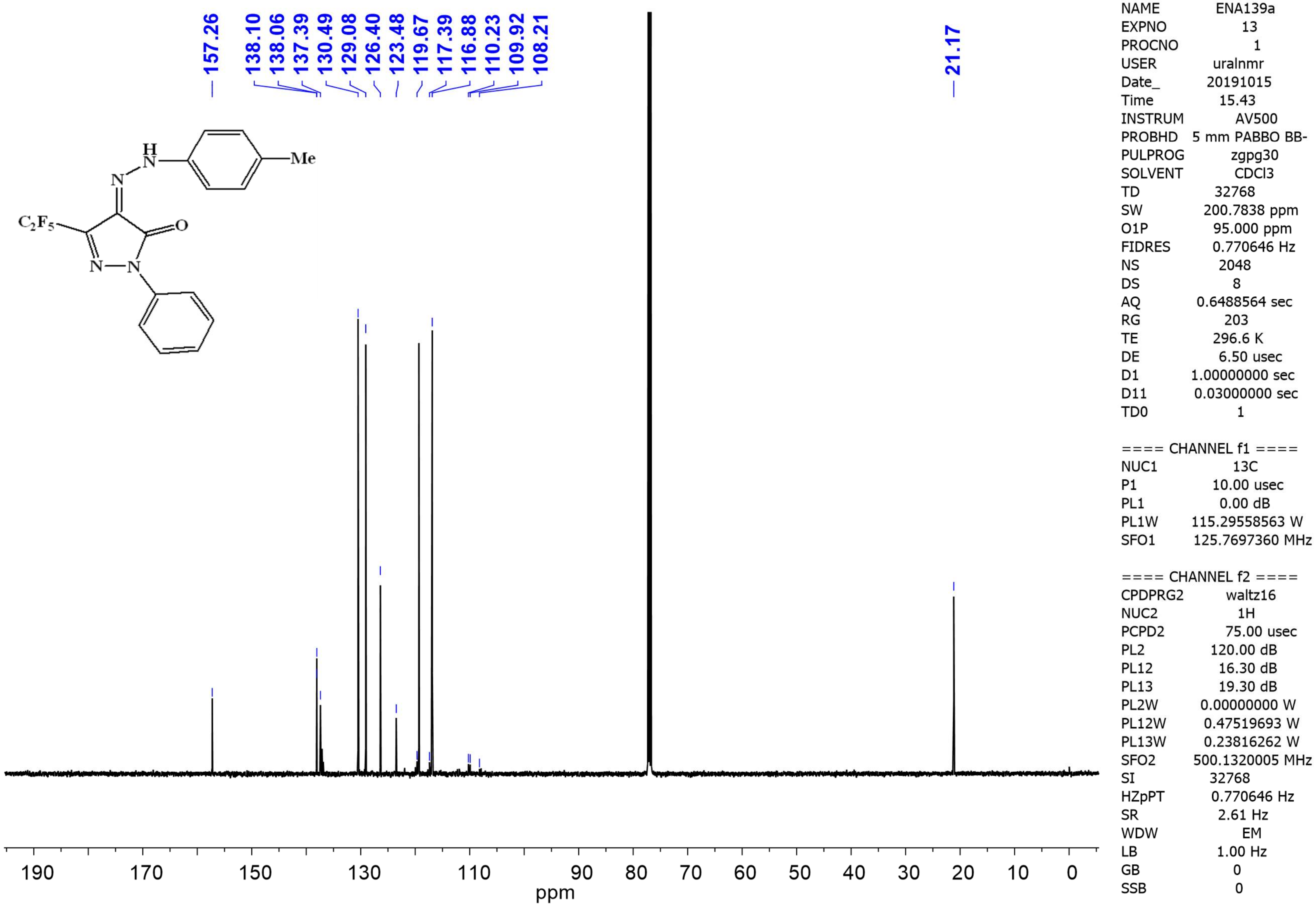

Figure S58. ^{19}F NMR spectrum of compound **6b**

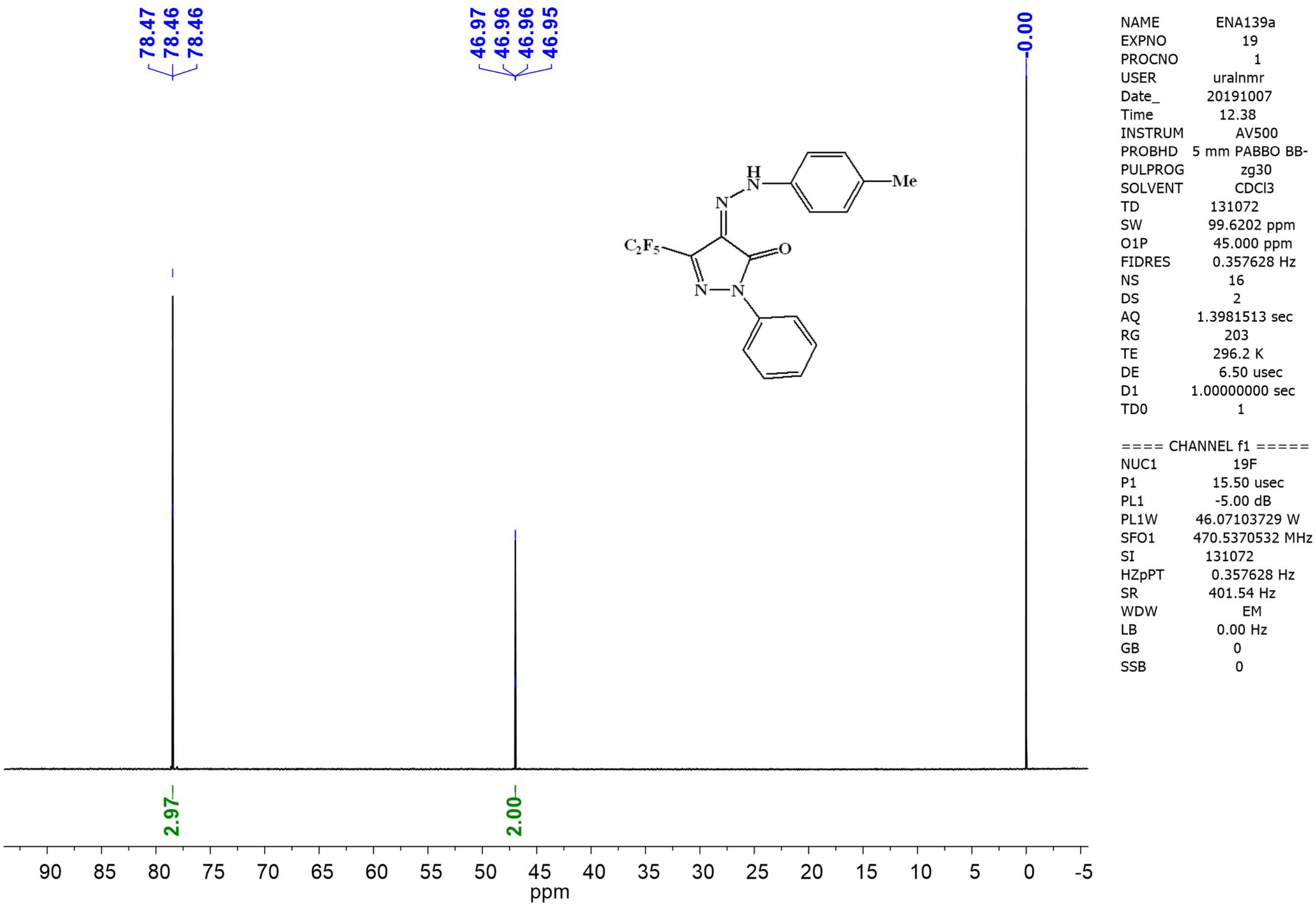

Figure S59. ^{1}H NMR spectrum of compound **6c**

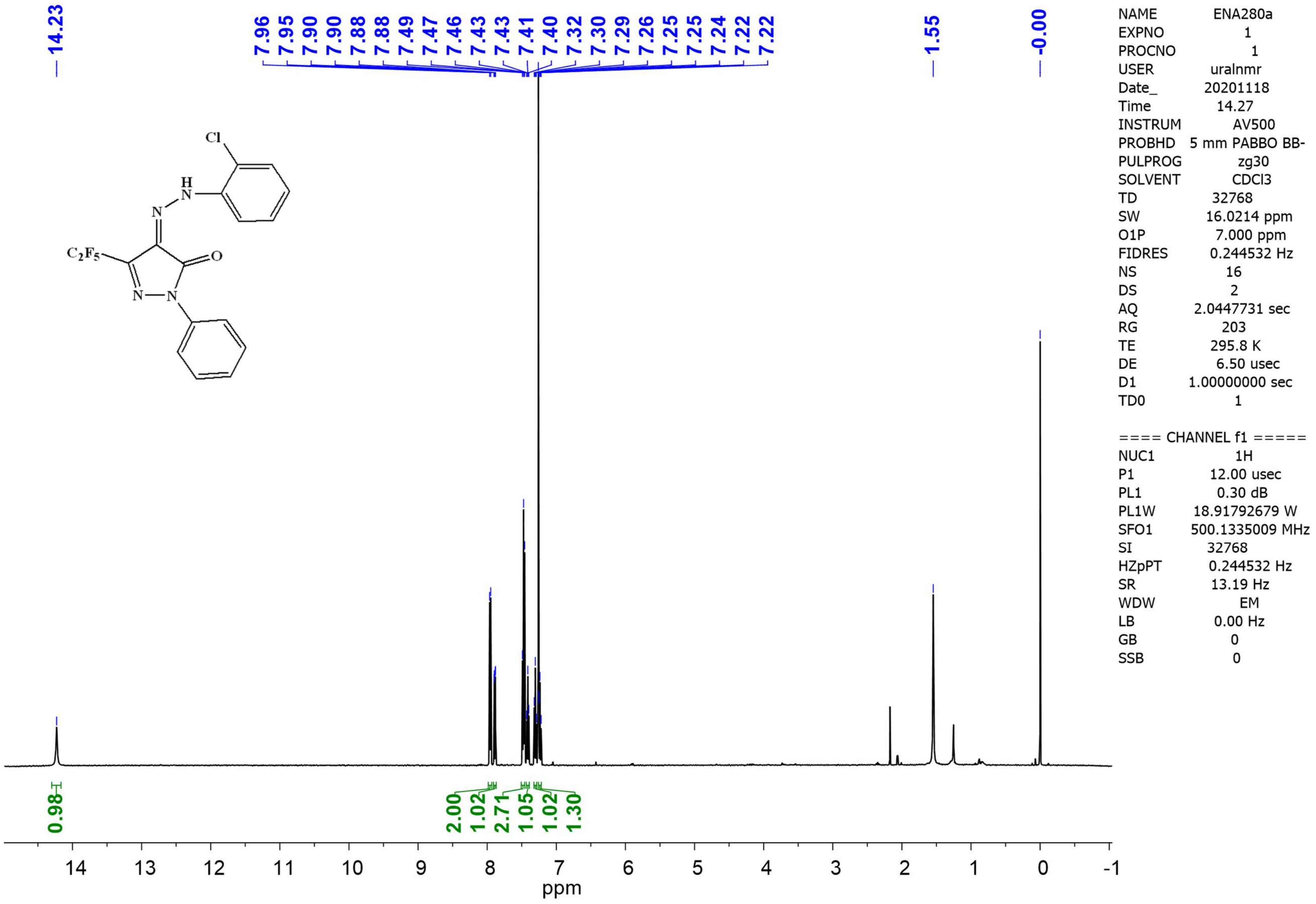

Figure S60. ^{13}C NMR spectrum of compound **6c**

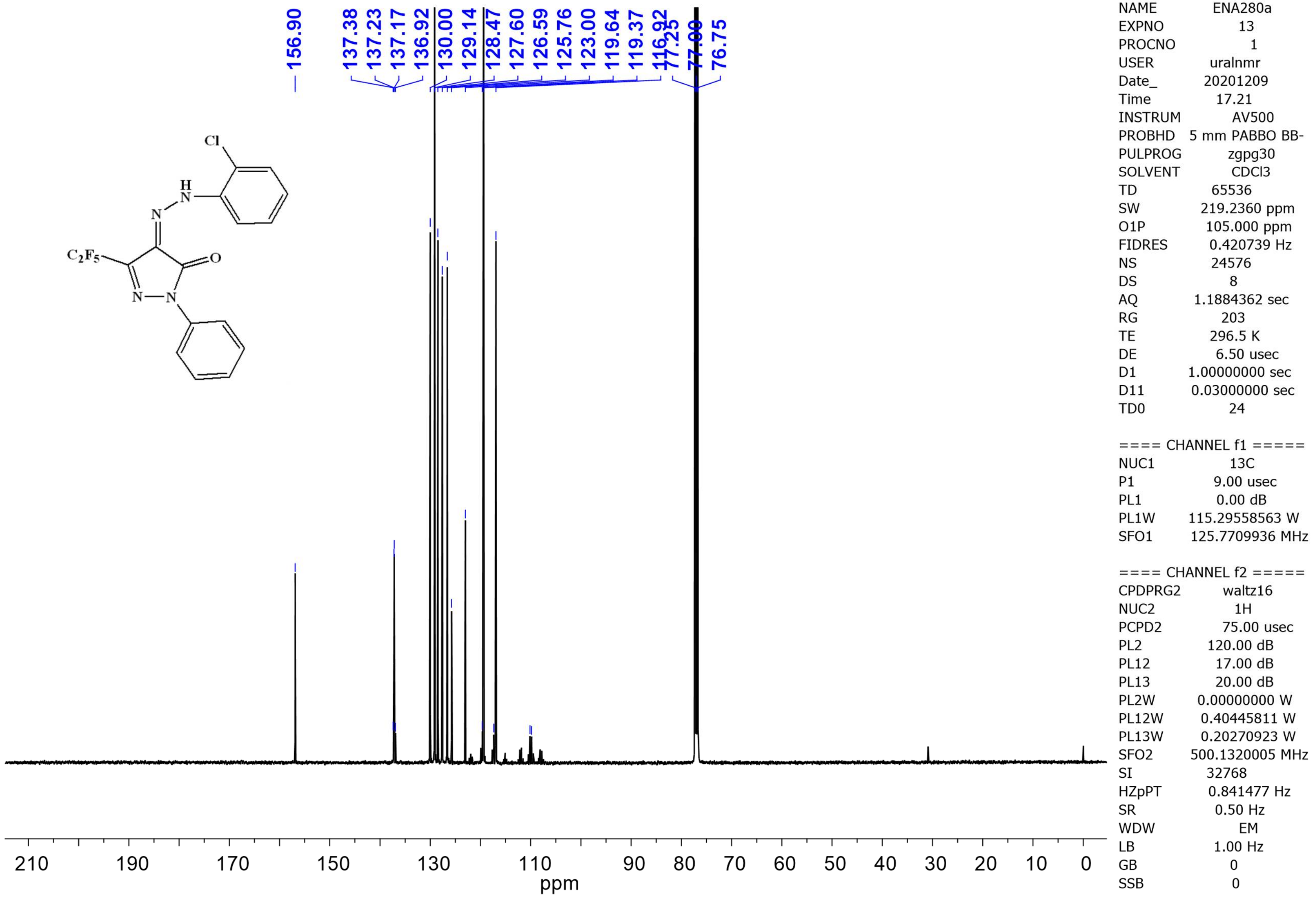

Figure S61. ^{19}F NMR spectrum of compound **6c**

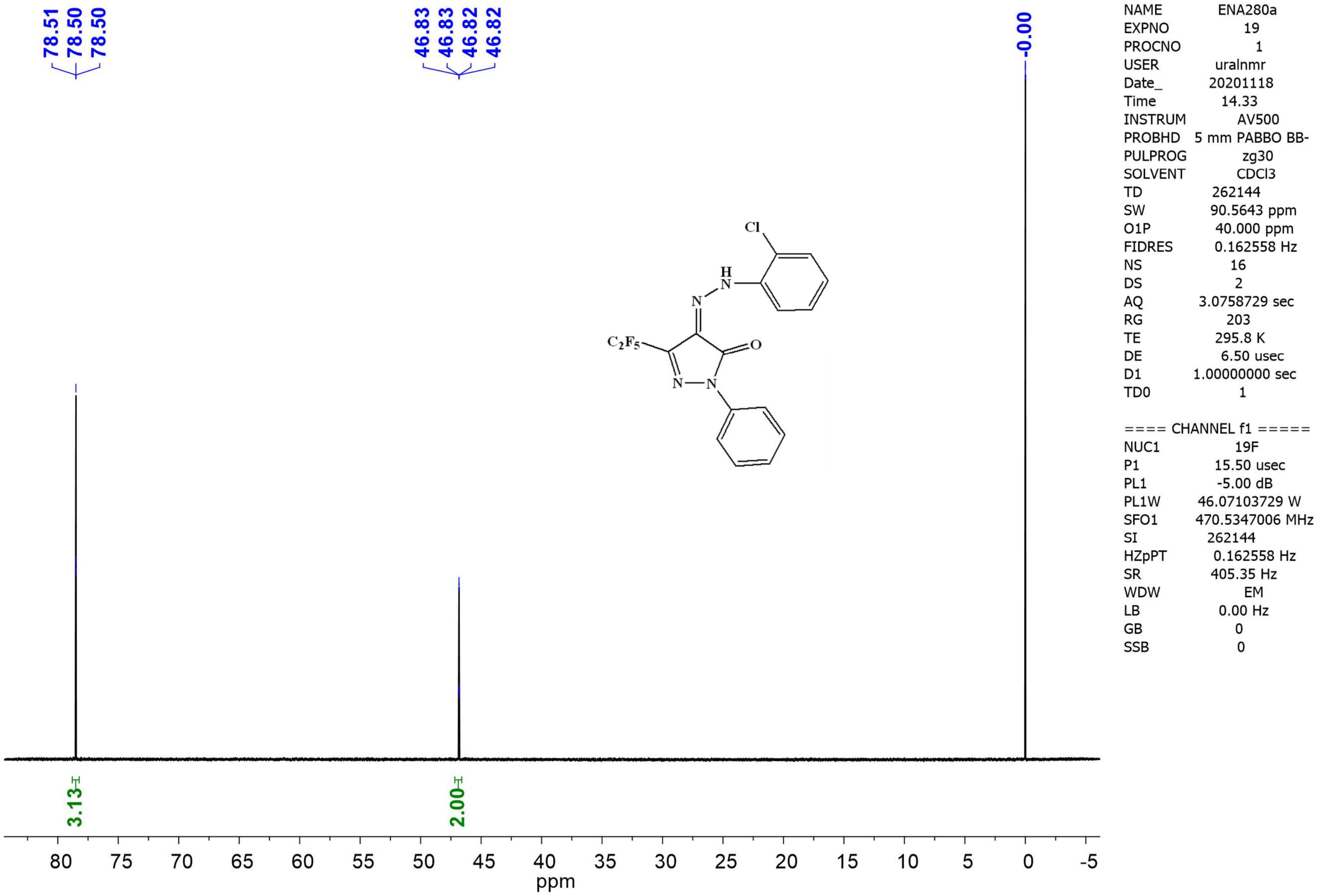

Figure S62. ^1H NMR spectrum of compound **6d**

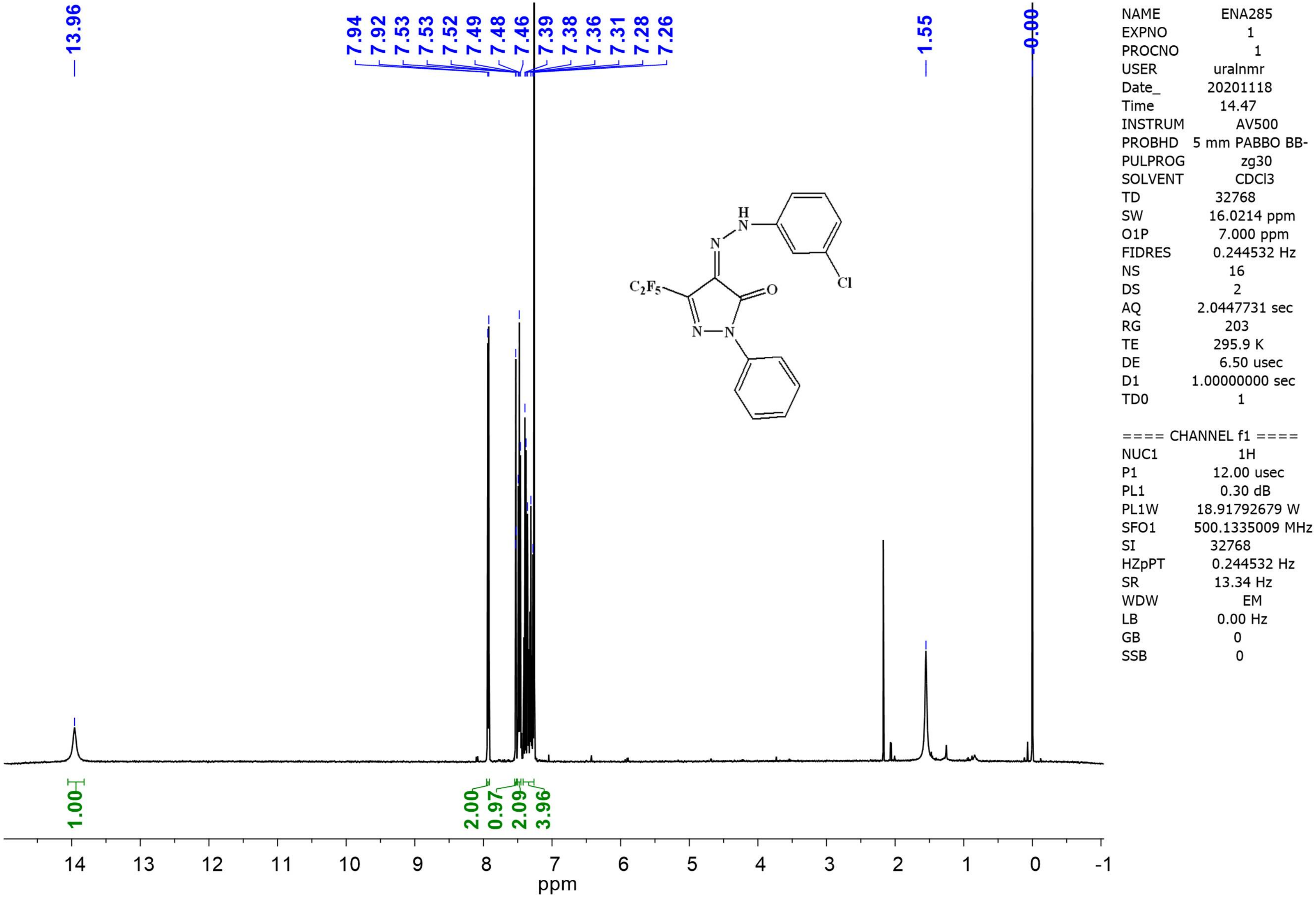

Figure S63. ^{13}C NMR spectrum of compound **6d**

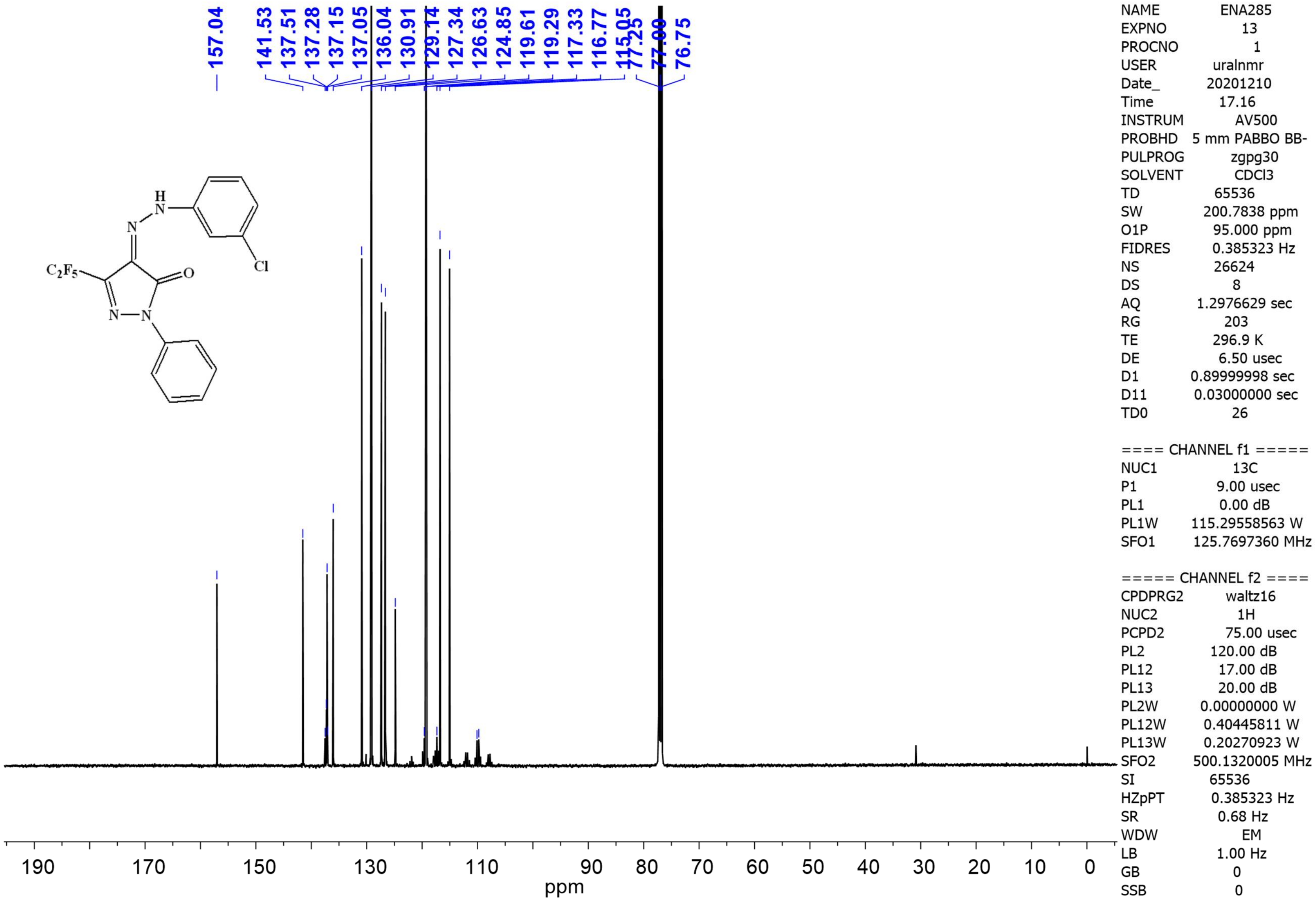

Figure S64. ^{19}F NMR spectrum of compound **6d**

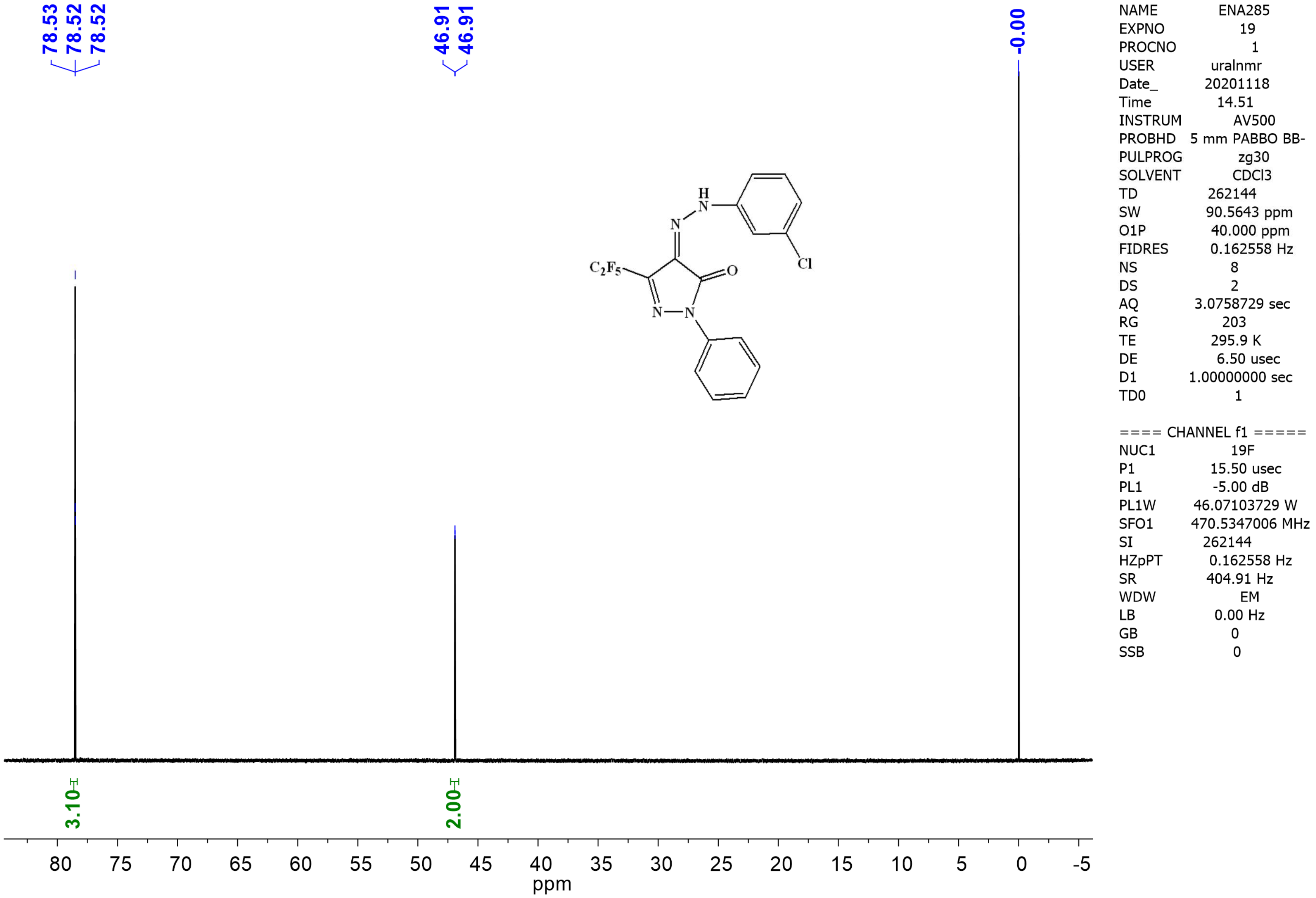

Figure S65. ^{1}H NMR spectrum of compound **6e**

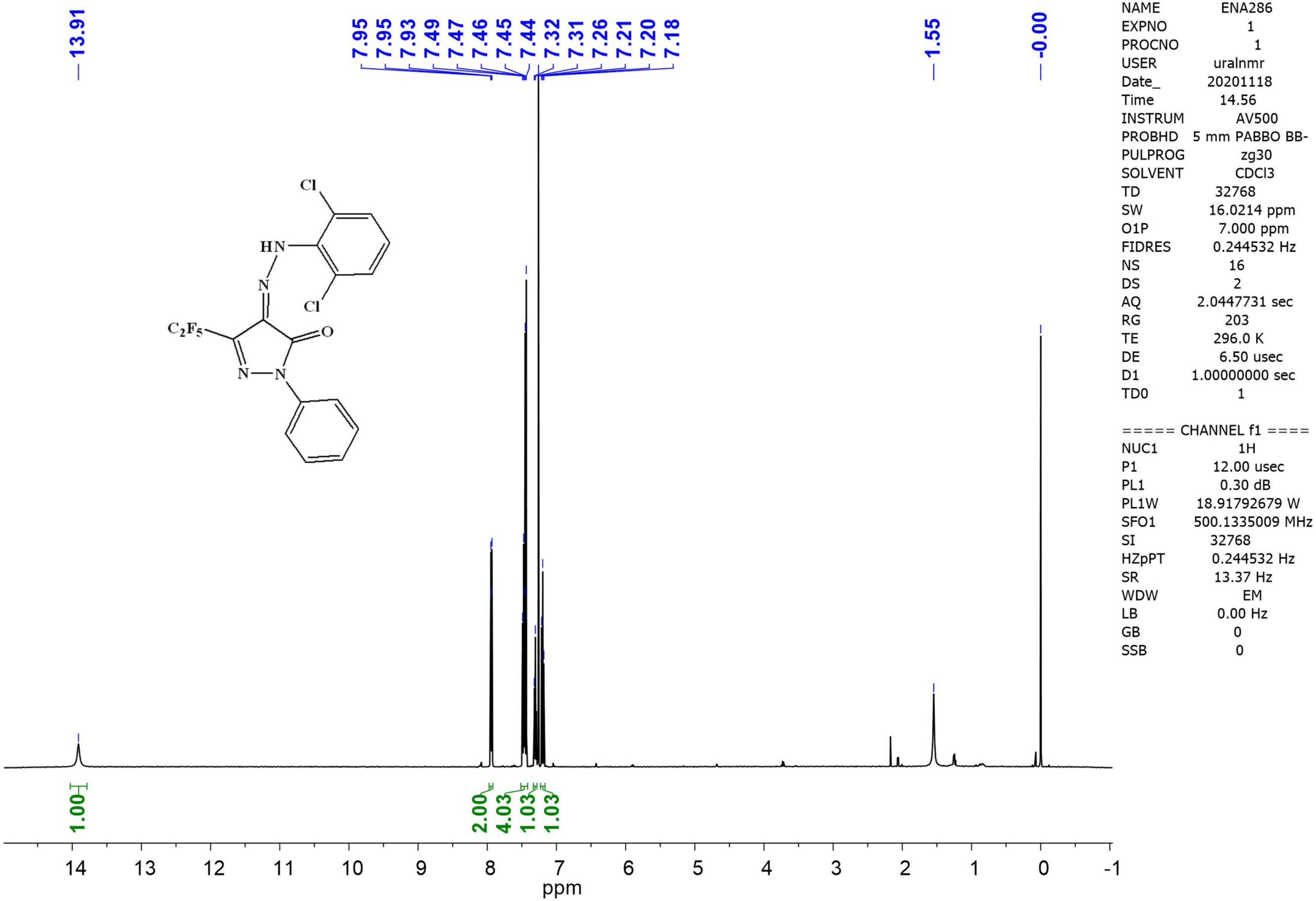

Figure S66. ^{13}C NMR spectrum of compound **6e**

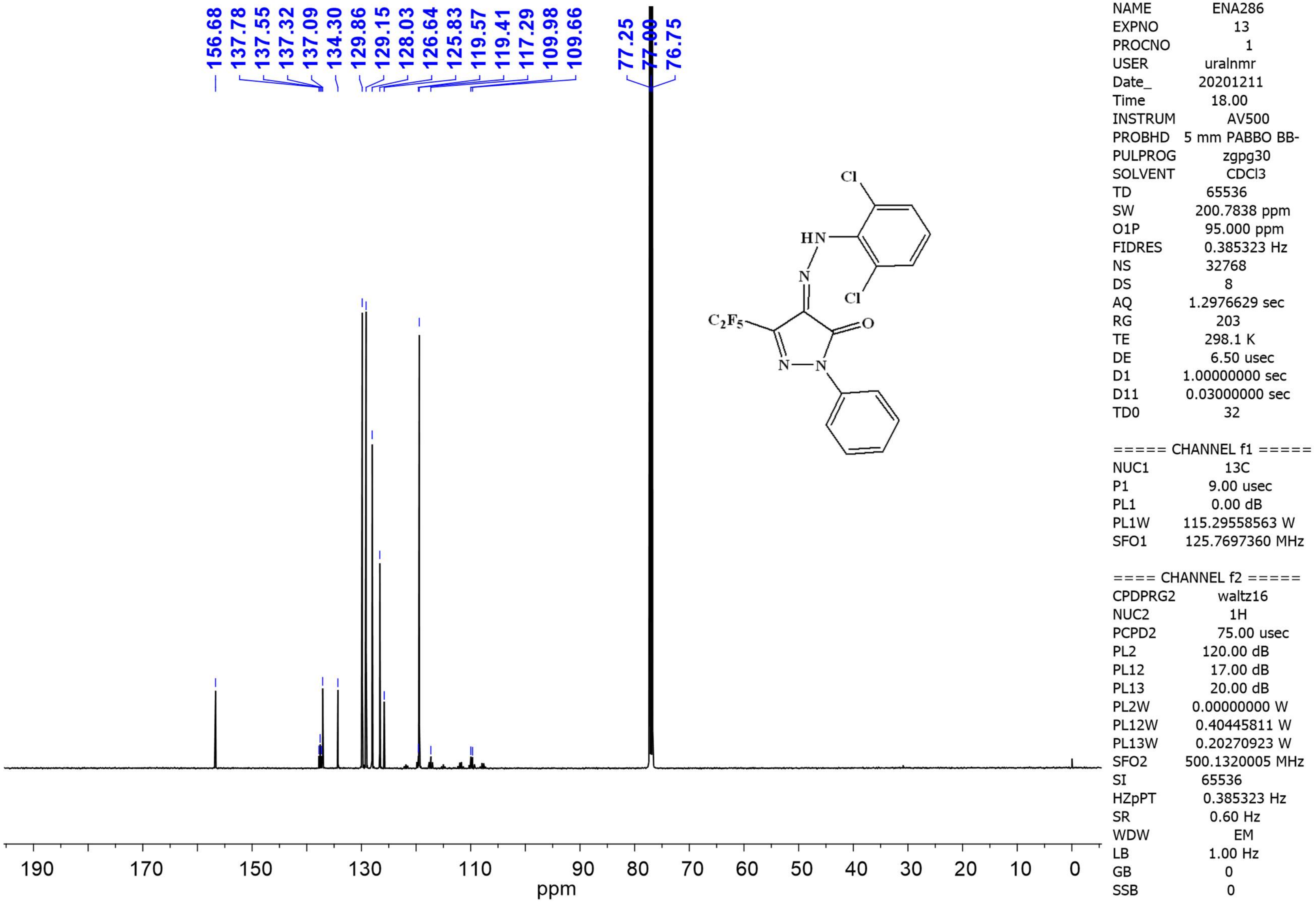

Figure S67. ^{19}F NMR spectrum of compound **6e**

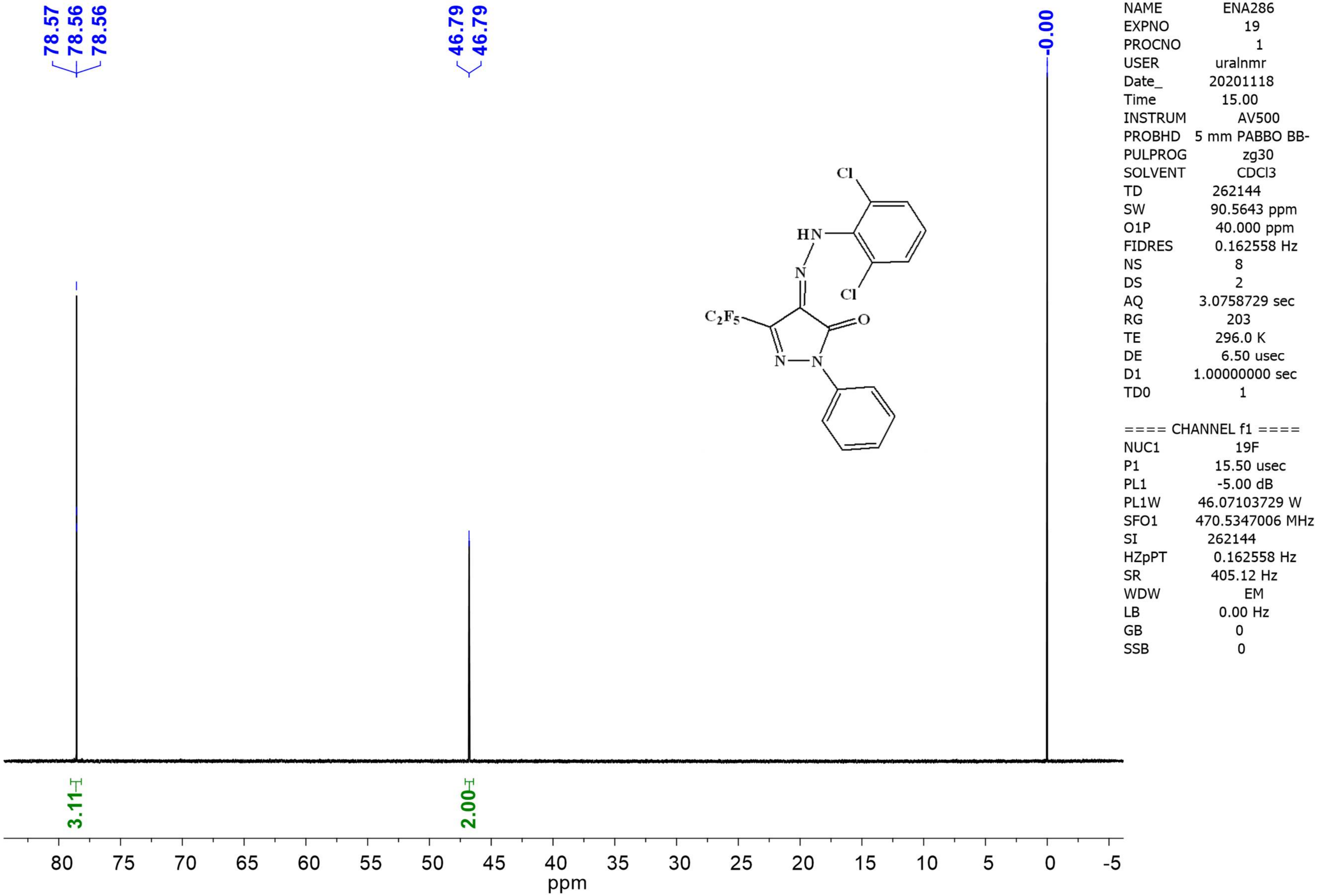

Figure S68. ^{1}H NMR spectrum of compound **6f**

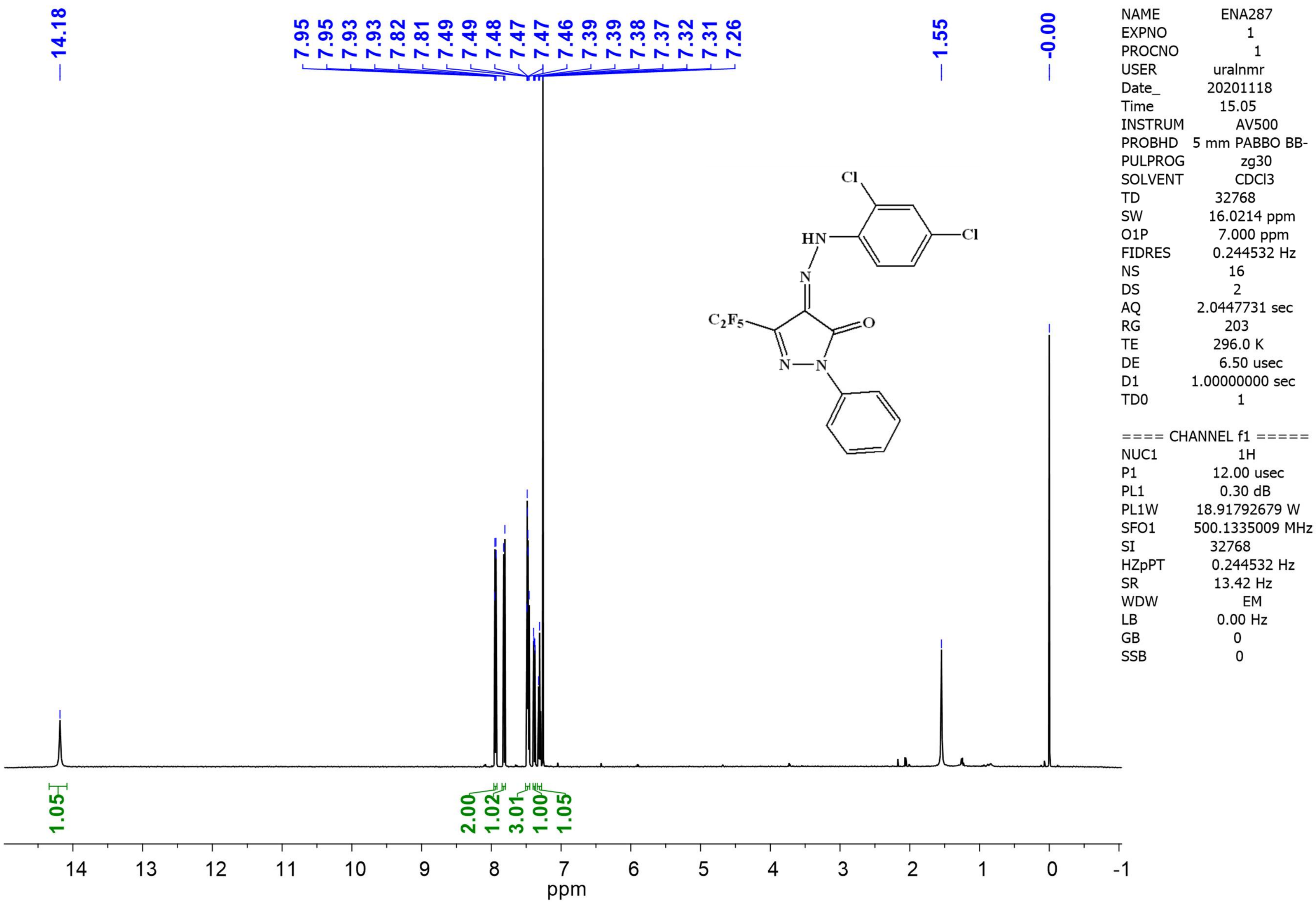

Figure S69. ^{13}C NMR spectrum of compound **6f**

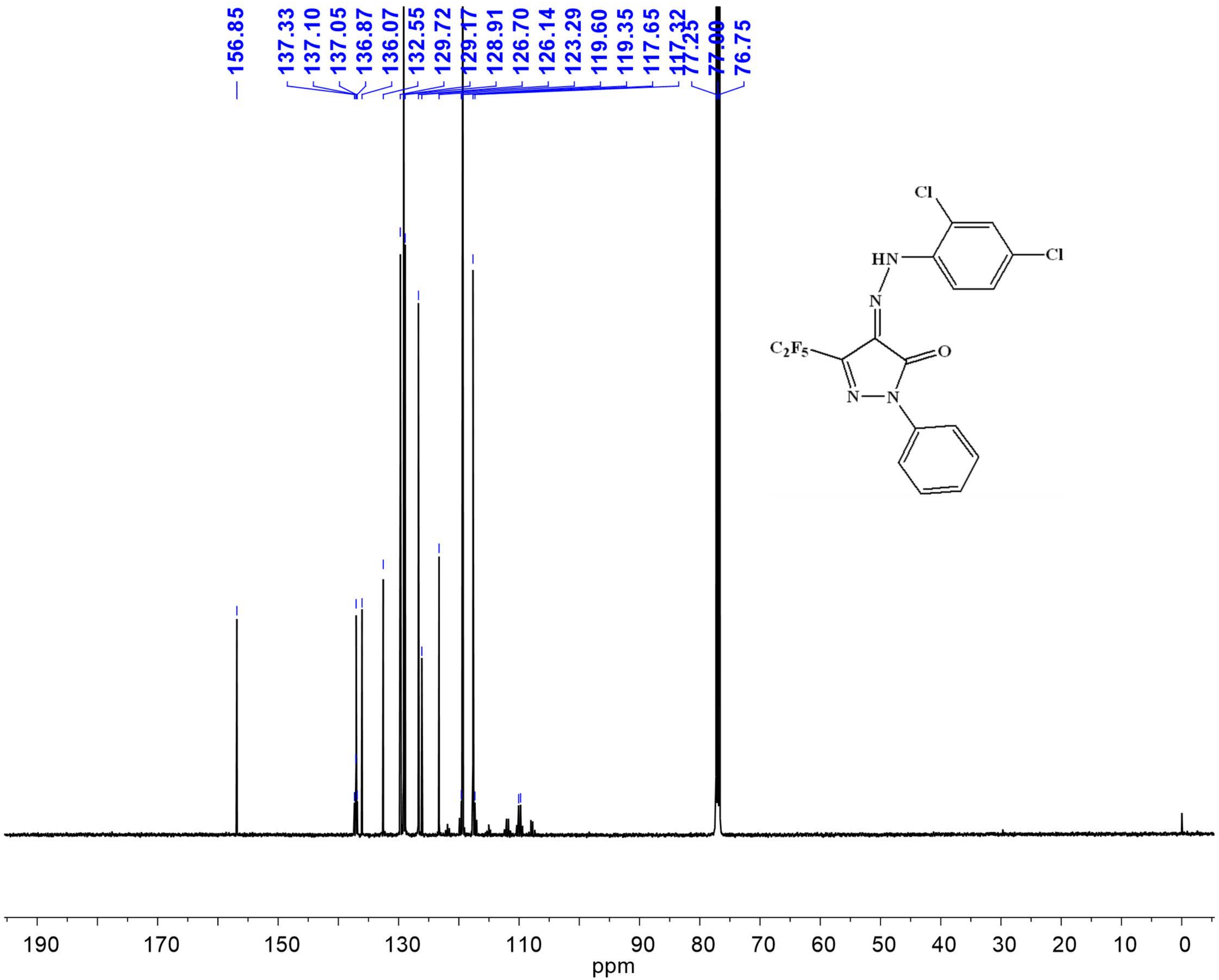

NAME ENA287
EXPNO 13
PROCNO 1
USER uralnmr
Date_ 20201214
Time 17.43
INSTRUM AV500
PROBHD 5 mm PABBO BB-
PULPROG zgpg30
SOLVENT CDCl3
TD 65536
SW 200.7838 ppm
O1P 95.000 ppm
FIDRES 0.385323 Hz
NS 24576
DS 8
AQ 1.2976629 sec
RG 203
TE 296.8 K
DE 6.50 usec
D1 0.89999998 sec
D11 0.03000000 sec
TD0 24
==== CHANNEL f1 ====
NUC1 13C
P1 9.00 usec
PL1 0.00 dB
PL1W 115.29558563 W
SFO1 125.7697360 MHz

==== CHANNEL f2 =====
CPDPRG2 waltz16
NUC2 1H
PCPD2 75.00 usec
PL2 120.00 dB
PL12 17.00 dB
PL13 20.00 dB
PL2W 0.00000000 W
PL12W 0.40445811 W
PL13W 0.20270923 W
SFO2 500.1320005 MHz
SI 65536
HZpPT 0.385323 Hz
SR 0.32 Hz
WDW EM
LB 1.00 Hz
GB 0
SSB 0

Figure S70. ^{19}F NMR spectrum of compound **6f**

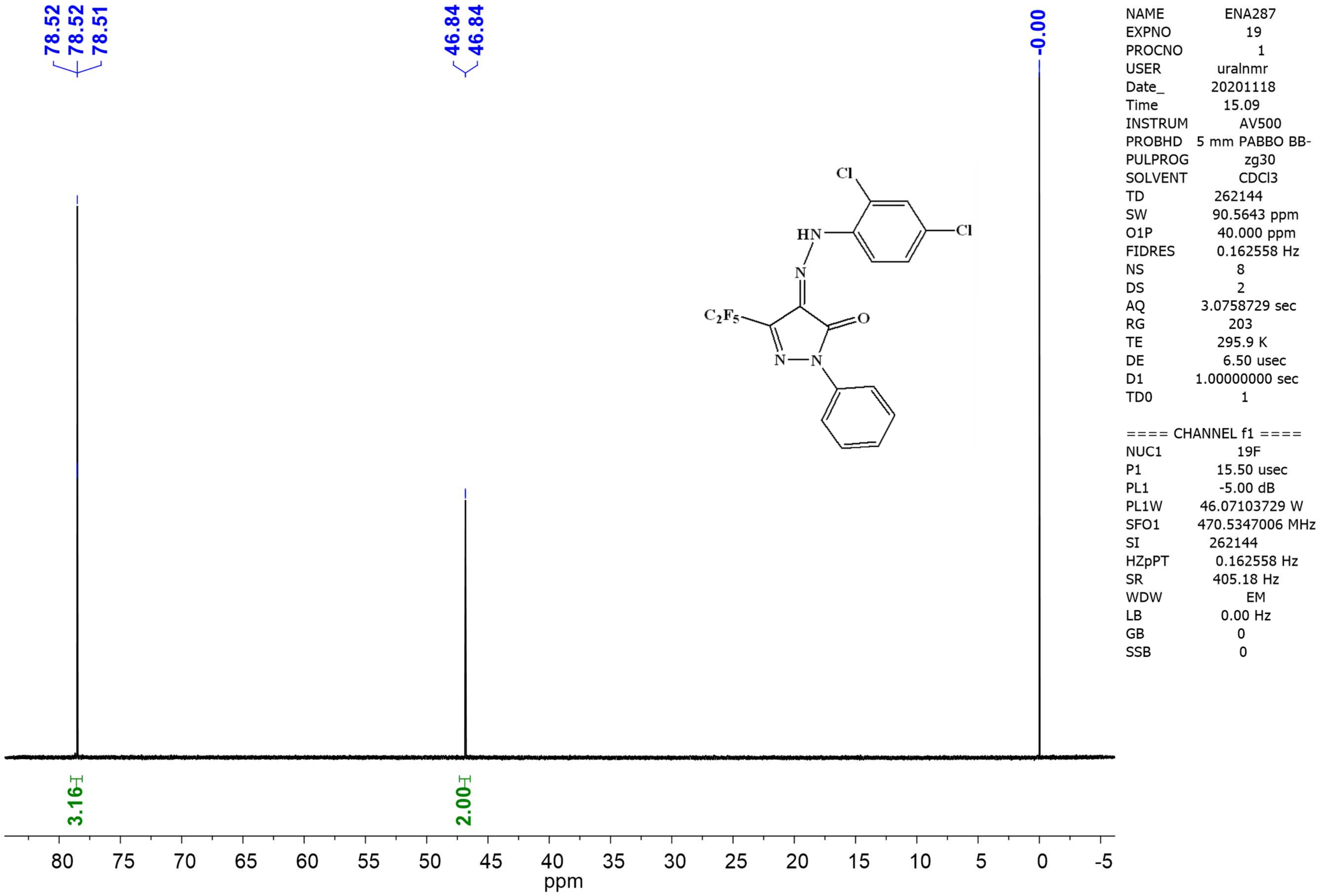

Figure S71. ^{1}H NMR spectrum of compound **6g**

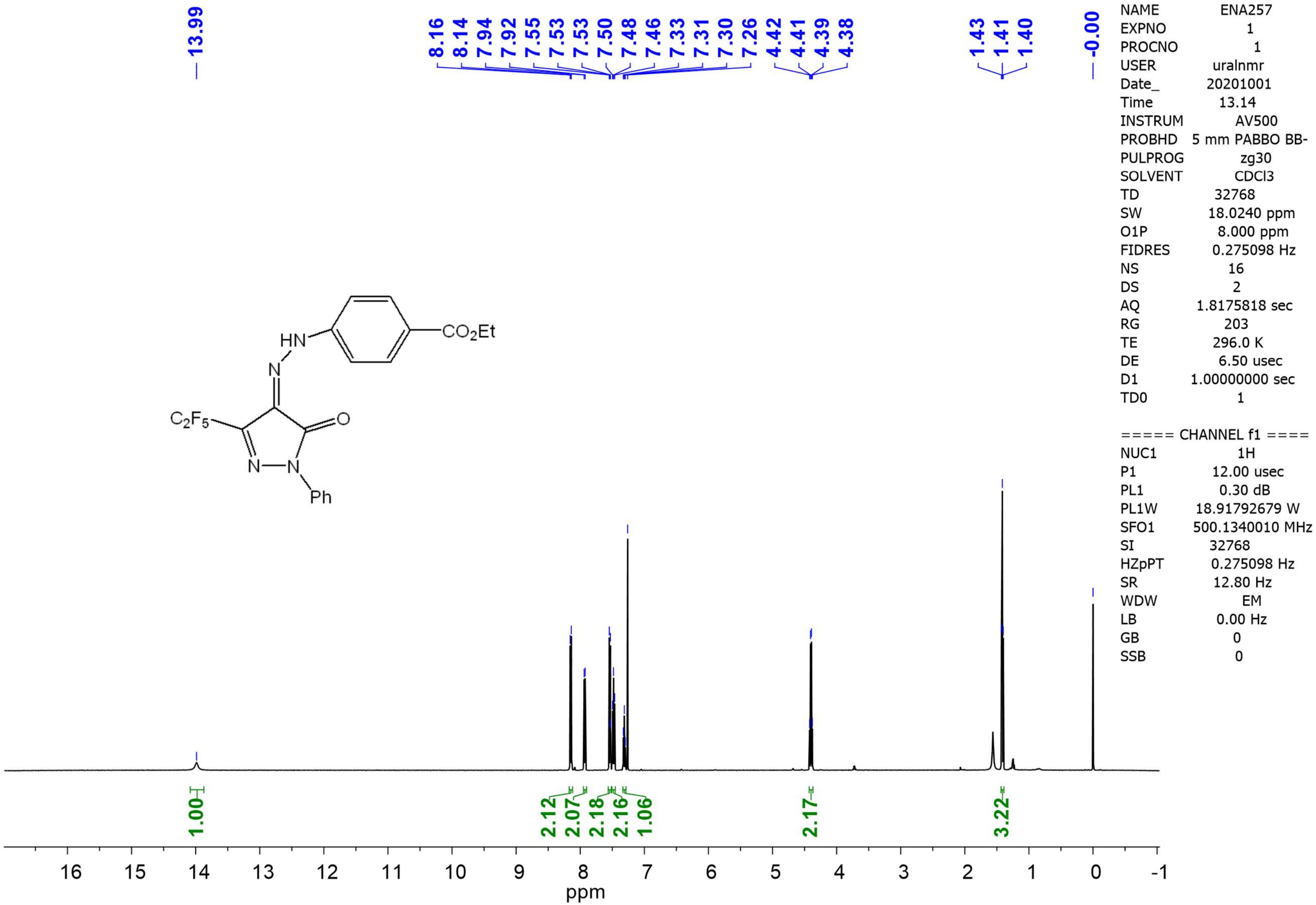

Figure S72. ^{13}C NMR spectrum of compound **6g**

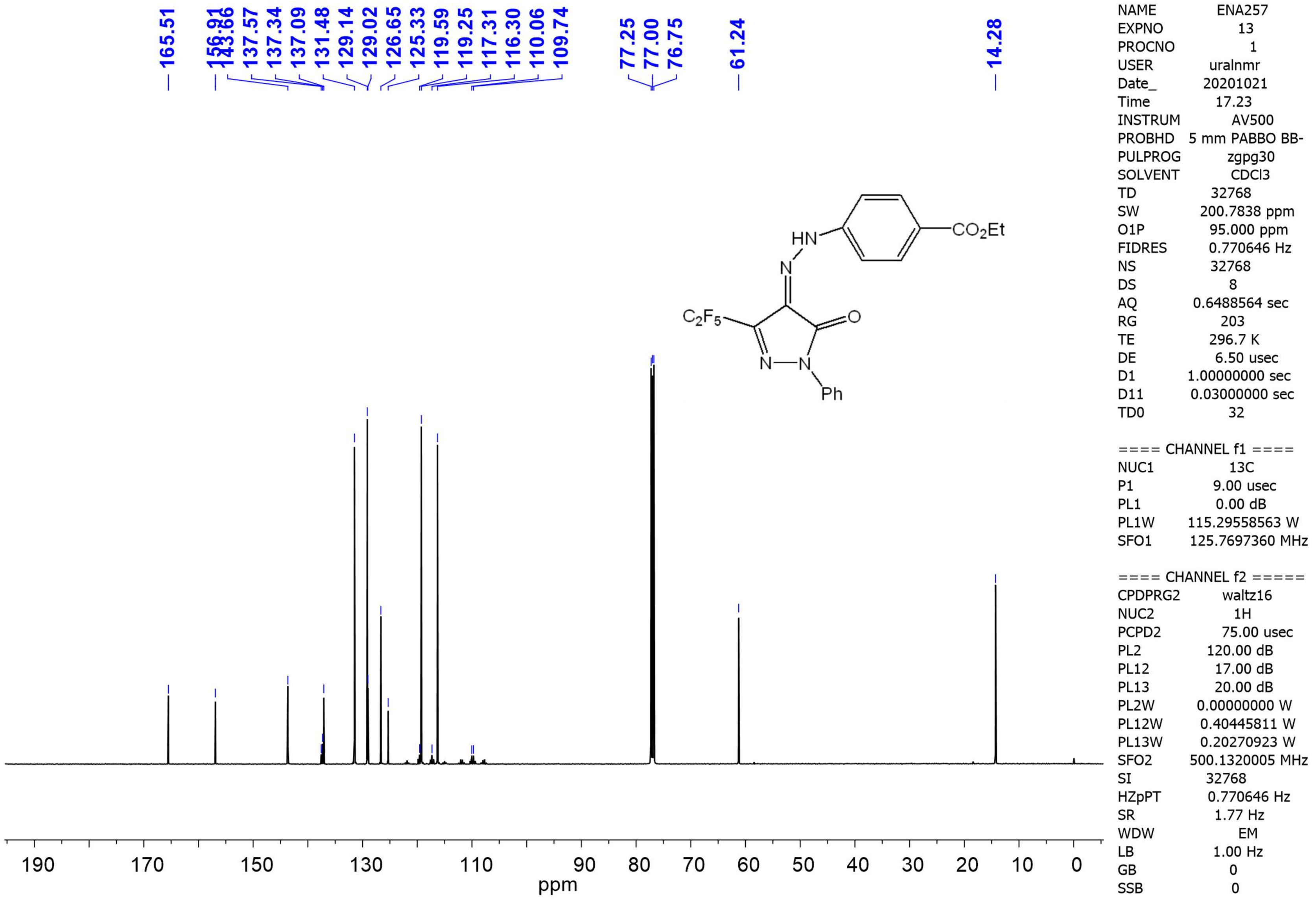

Figure S73. ^{19}F NMR spectrum of compound **6g**

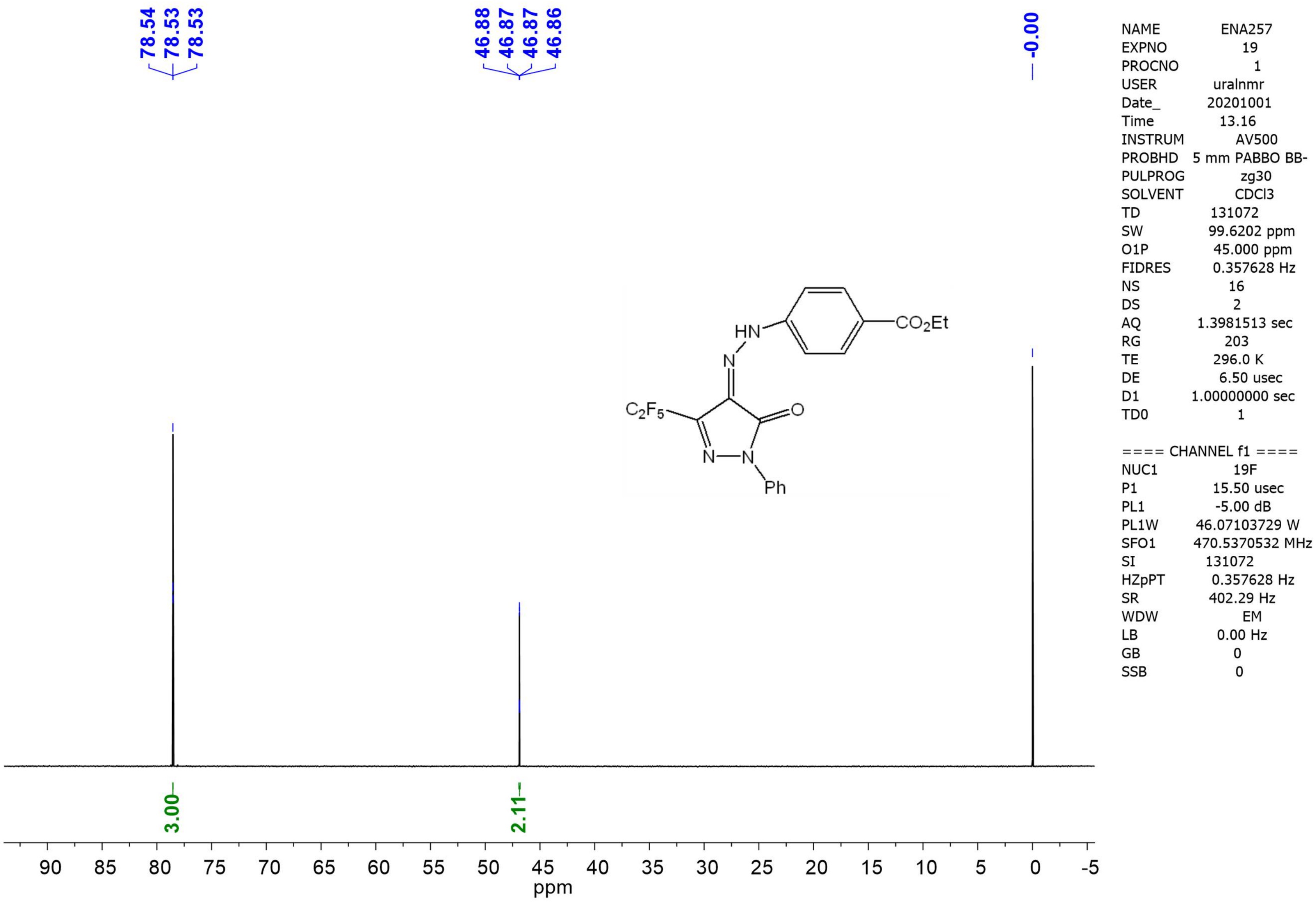

Figure S74. ^{1}H NMR spectrum of compound **6h**

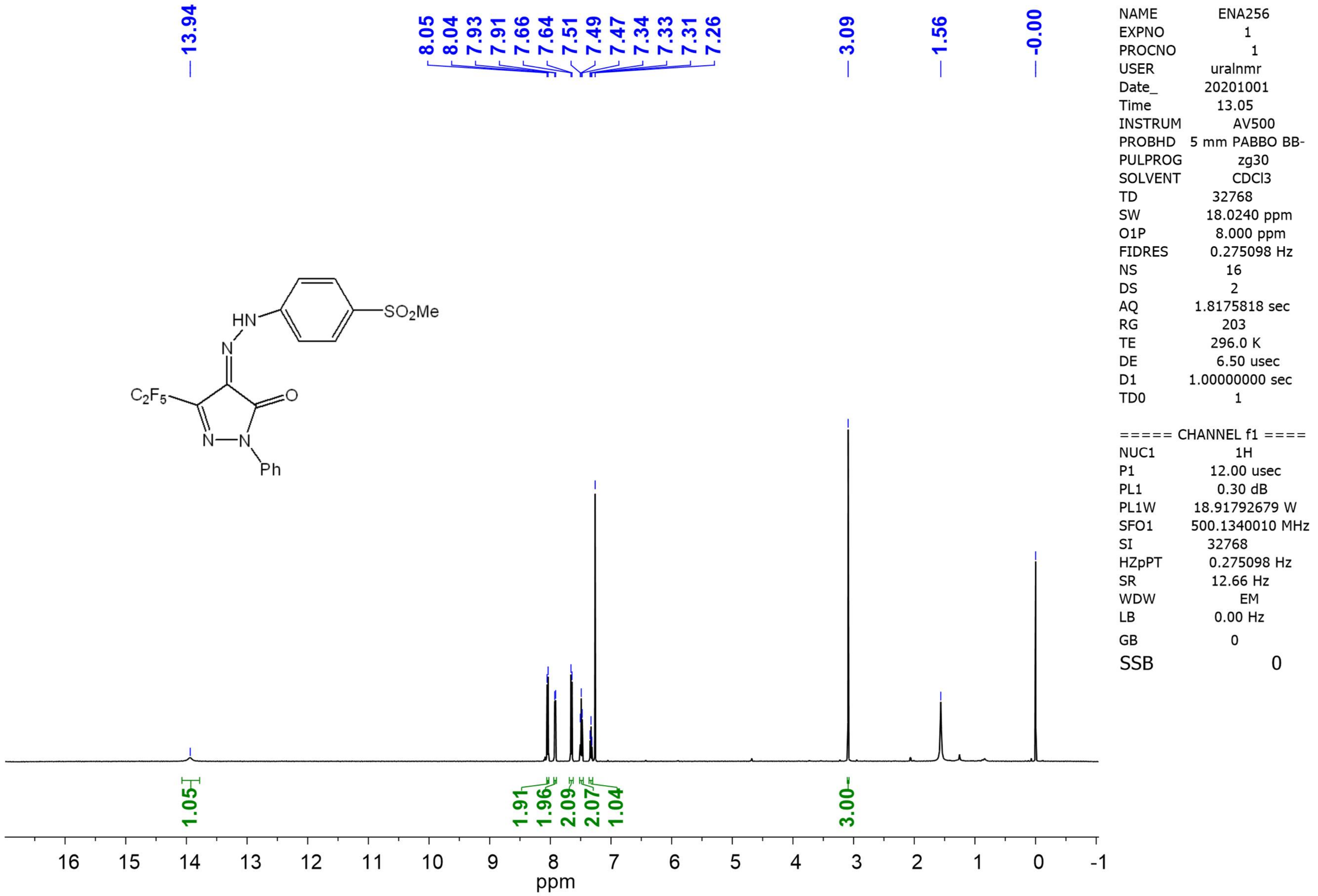

Figure S75. ^{13}C NMR spectrum of compound **6h**

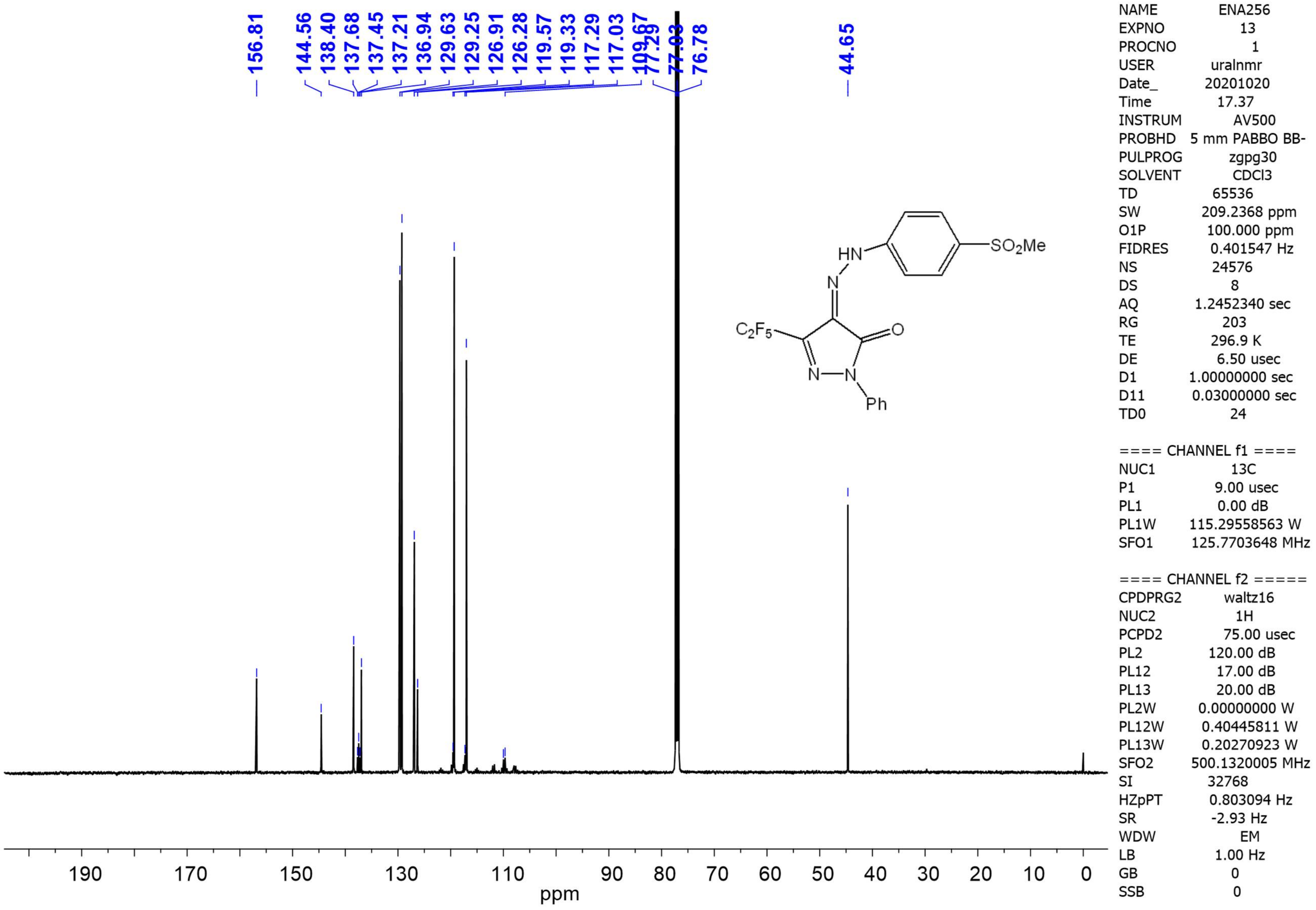

Figure S76. ^{19}F NMR spectrum of compound **6h**

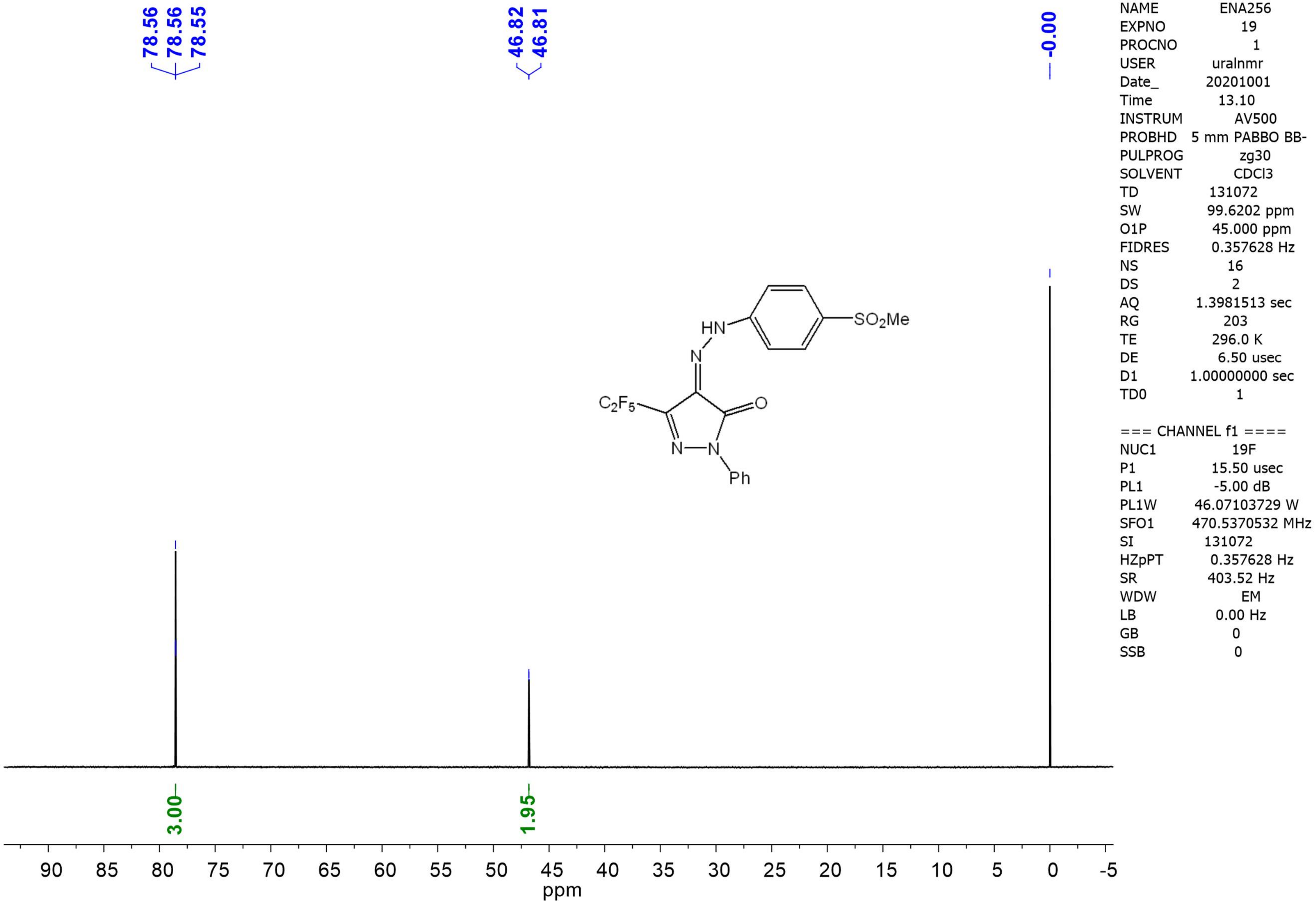

Figure S77. ^{1}H NMR spectrum of compound **6i**

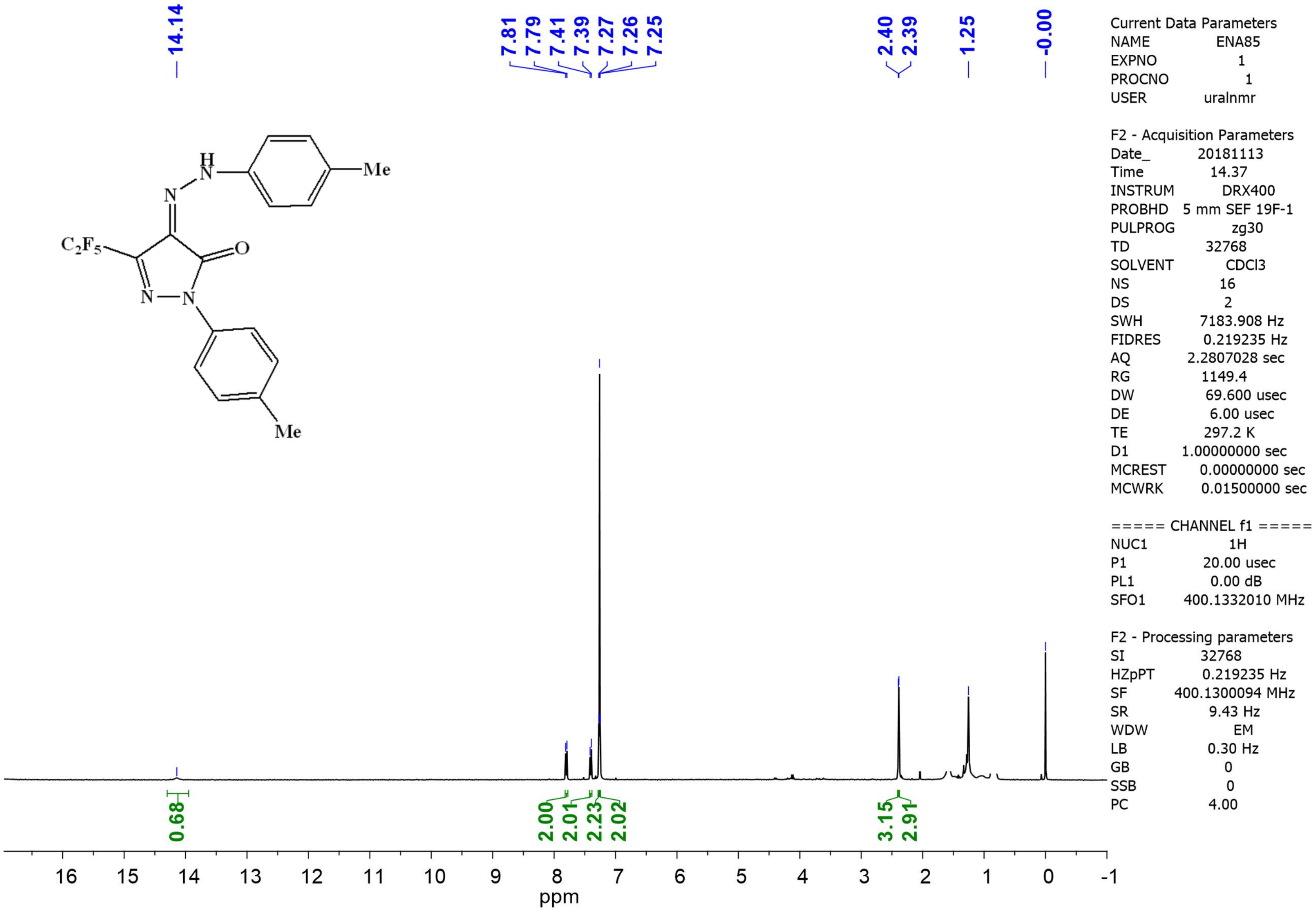

Figure S78. ^{19}F NMR spectrum of compound **6i**

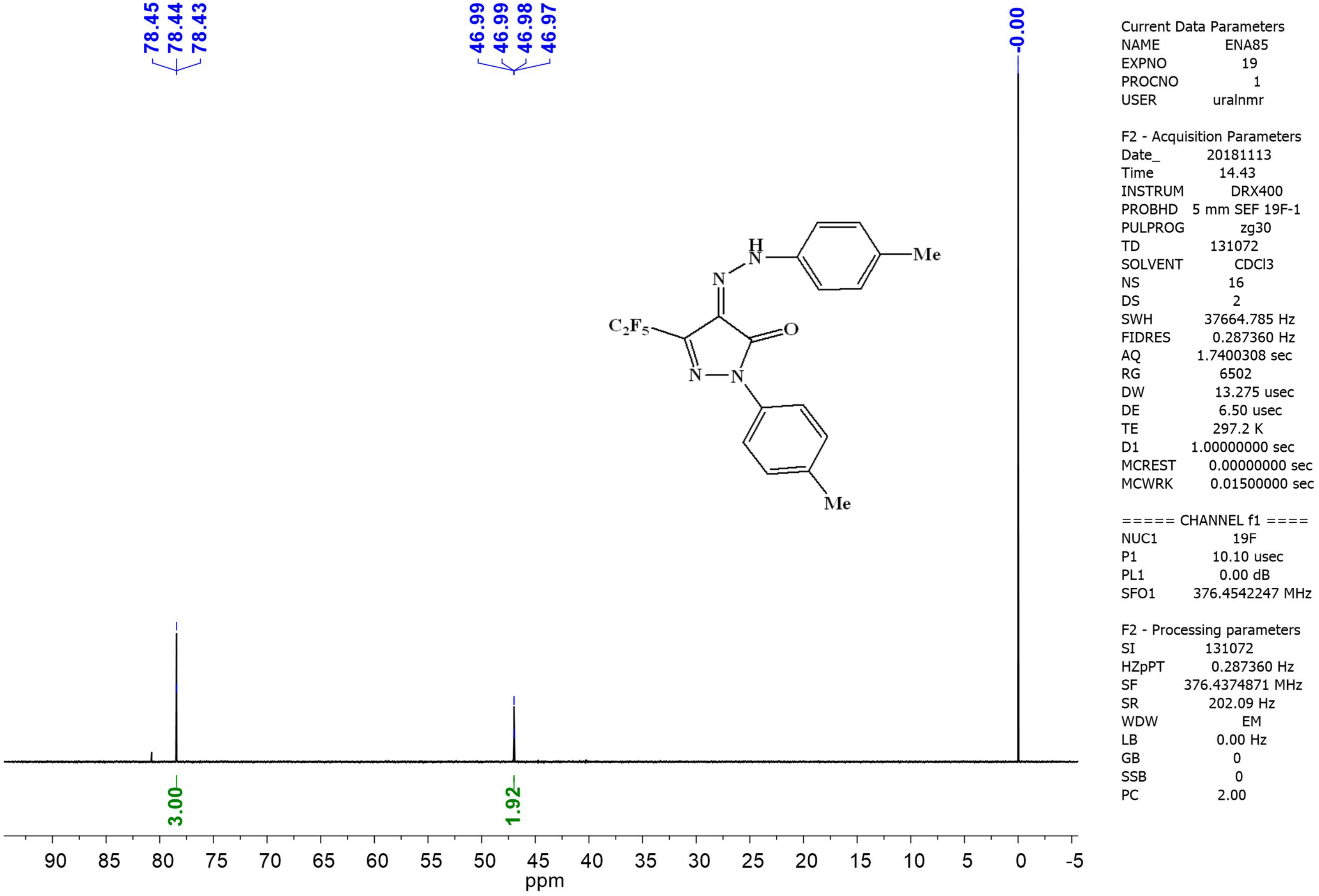

Figure S79. ^{1}H NMR spectrum of compound **7a**

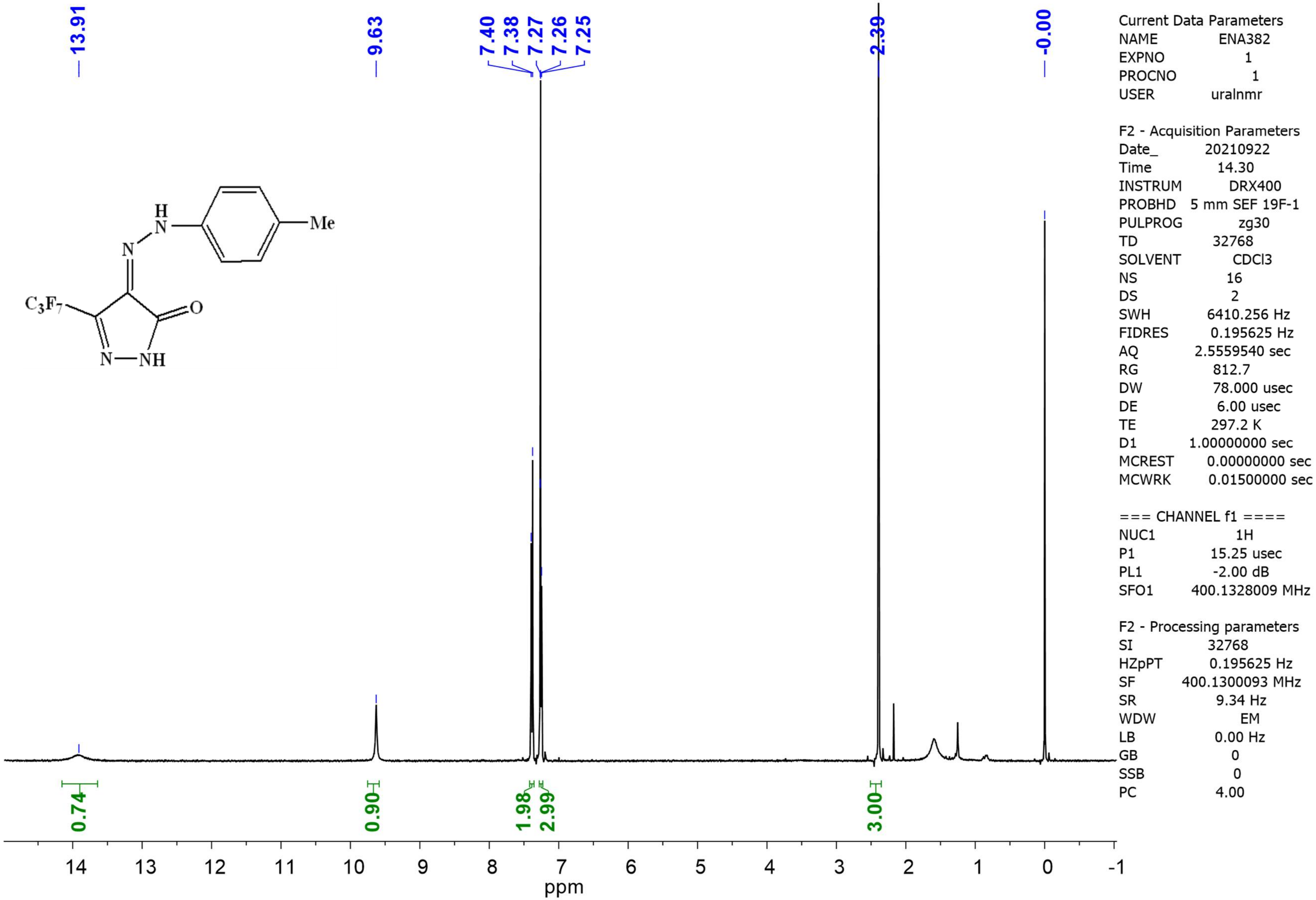

Figure S80. ¹³C NMR spectrum of compound **7a**

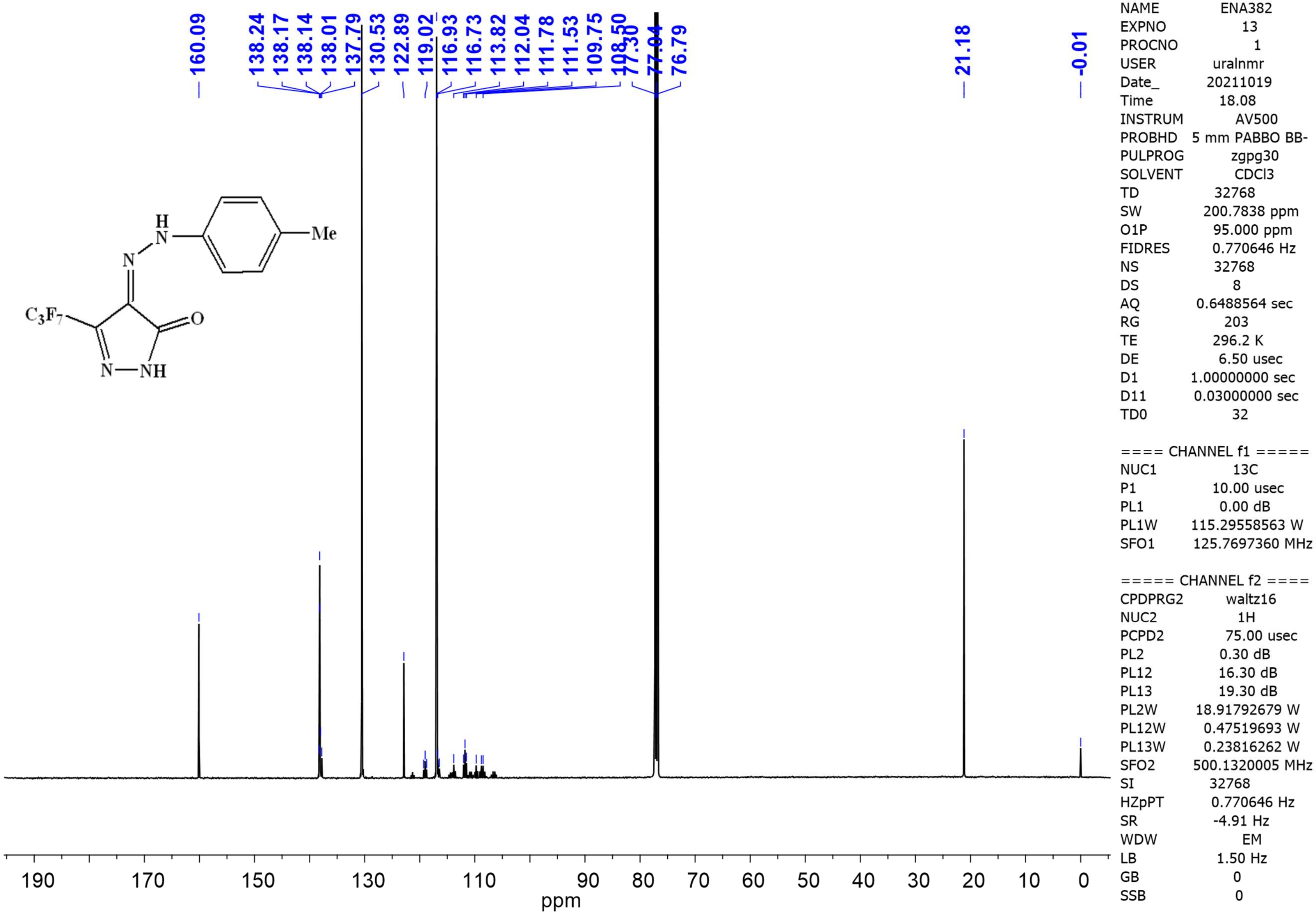

Figure S81. ^{19}F NMR spectrum of compound **7a**

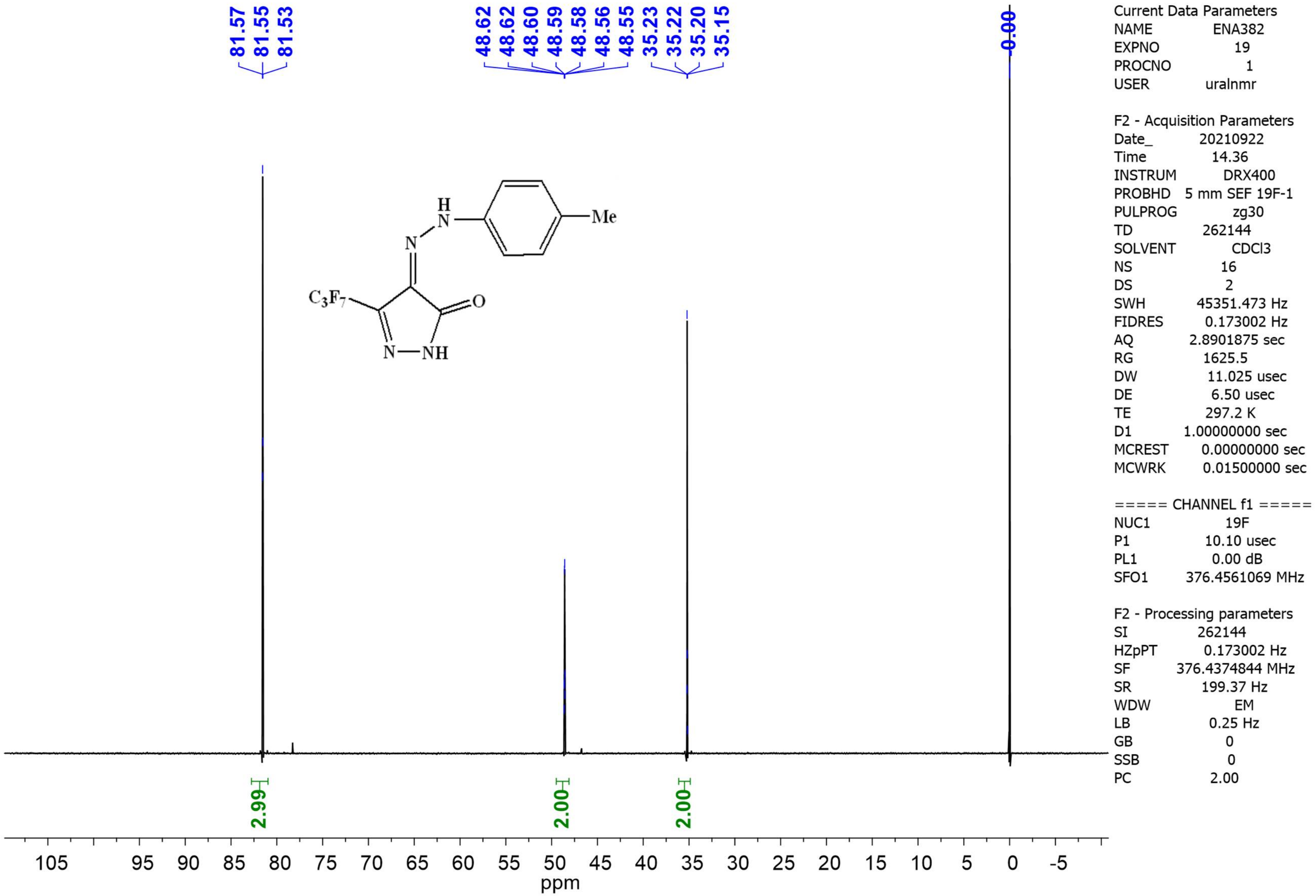

Figure S82. ^{1}H NMR spectrum of compound **7b**

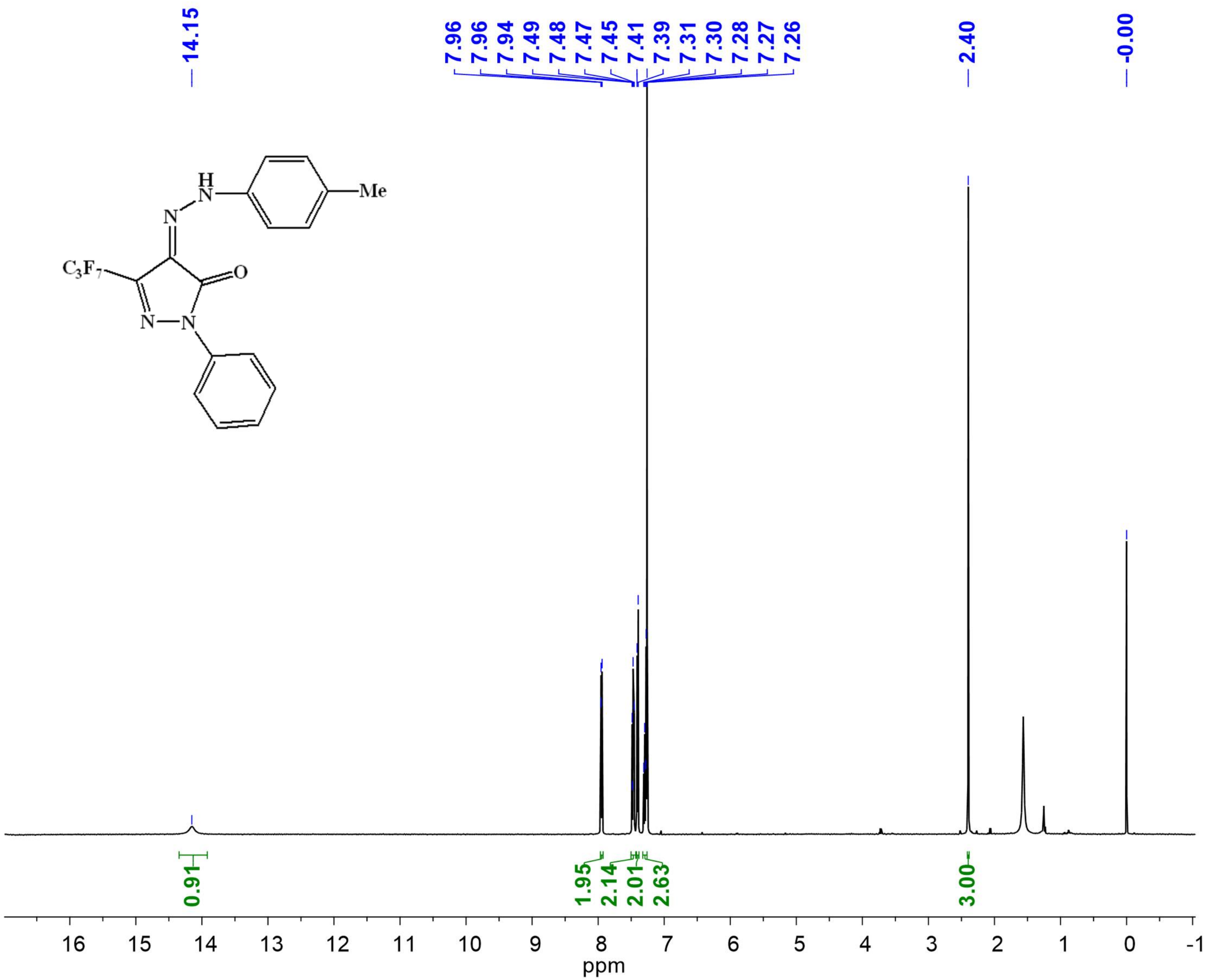

NAME	ENA178
EXPNO	1
PROCNO	1
USER	uralnmr
Date_	20191009
Time	13.39
INSTRUM	AV500
PROBHD	5 mm PABBO BB-
PULPROG	zg30
SOLVENT	CDCl3
TD	32768
SW	18.0240 ppm
O1P	8.000 ppm
FIDRES	0.275098 Hz
NS	16
DS	2
AQ	1.8175818 sec
RG	203
TE	296.0 K
DE	16.00 usec
D1	1.00000000 sec
TD0	1
==== CHANNEL f1 ====	
NUC1	1H
P1	12.00 usec
PL1	0.30 dB
PL1W	18.91792679 W
SFO1	500.1340010 MHz
SI	32768
HZpPT	0.275098 Hz
SR	12.93 Hz
WDW	EM
LB	0.00 Hz
GB	0
SSB	0

Figure S83. ^{13}C NMR spectrum of compound **7b**

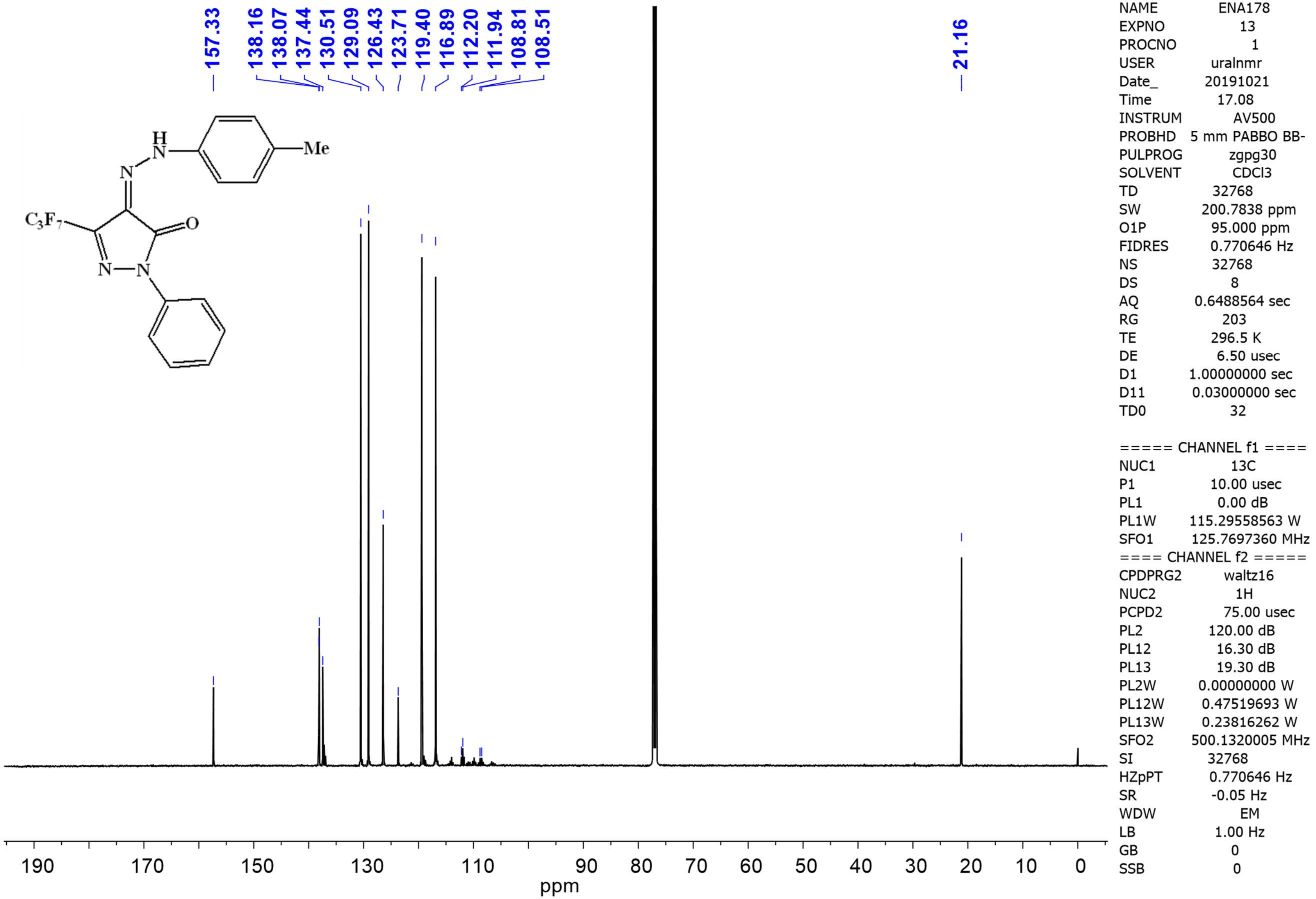

Figure S84. ^{19}F NMR spectrum of compound **7b**

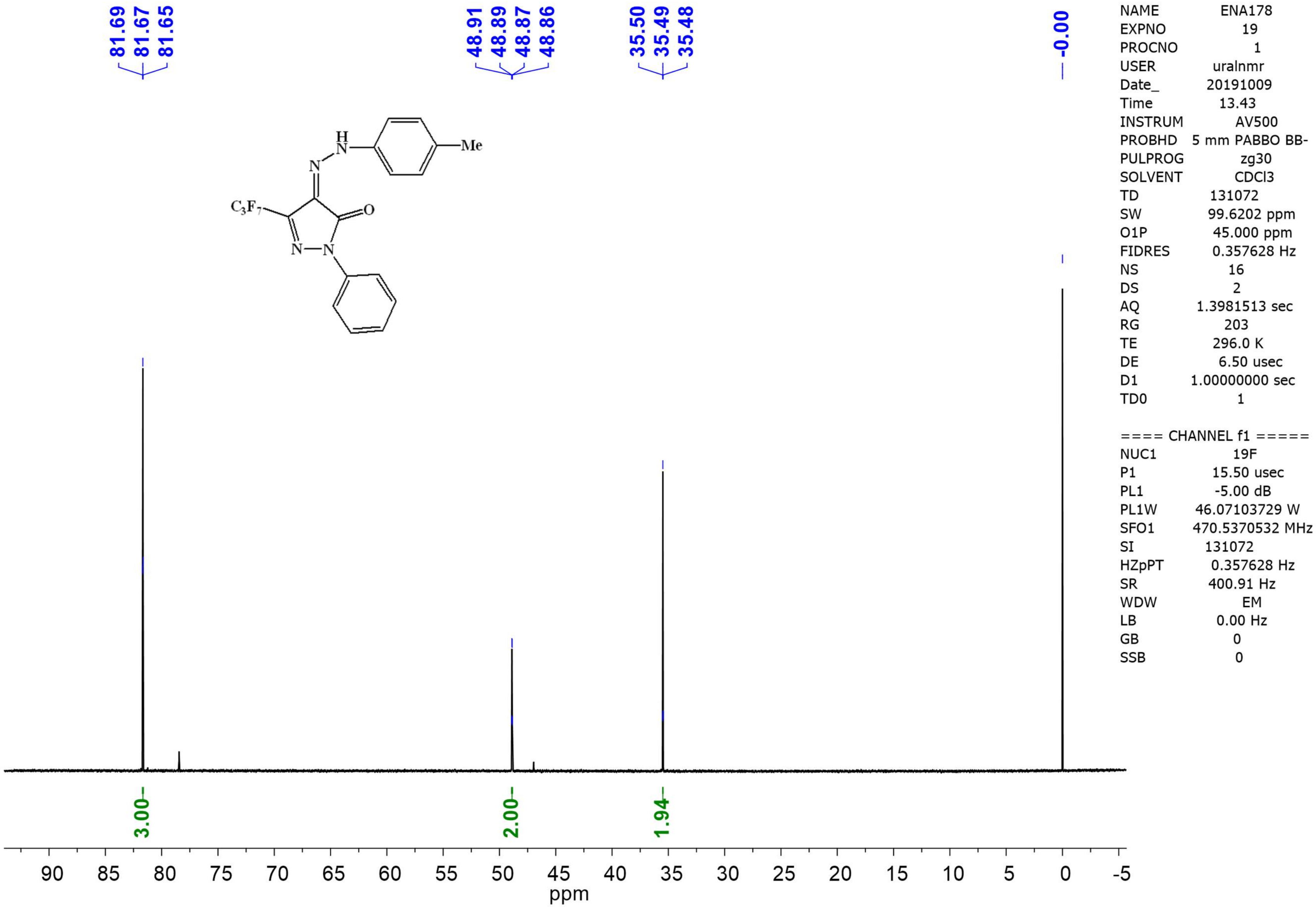

Figure S85. ^{1}H NMR spectrum of compound **8a**

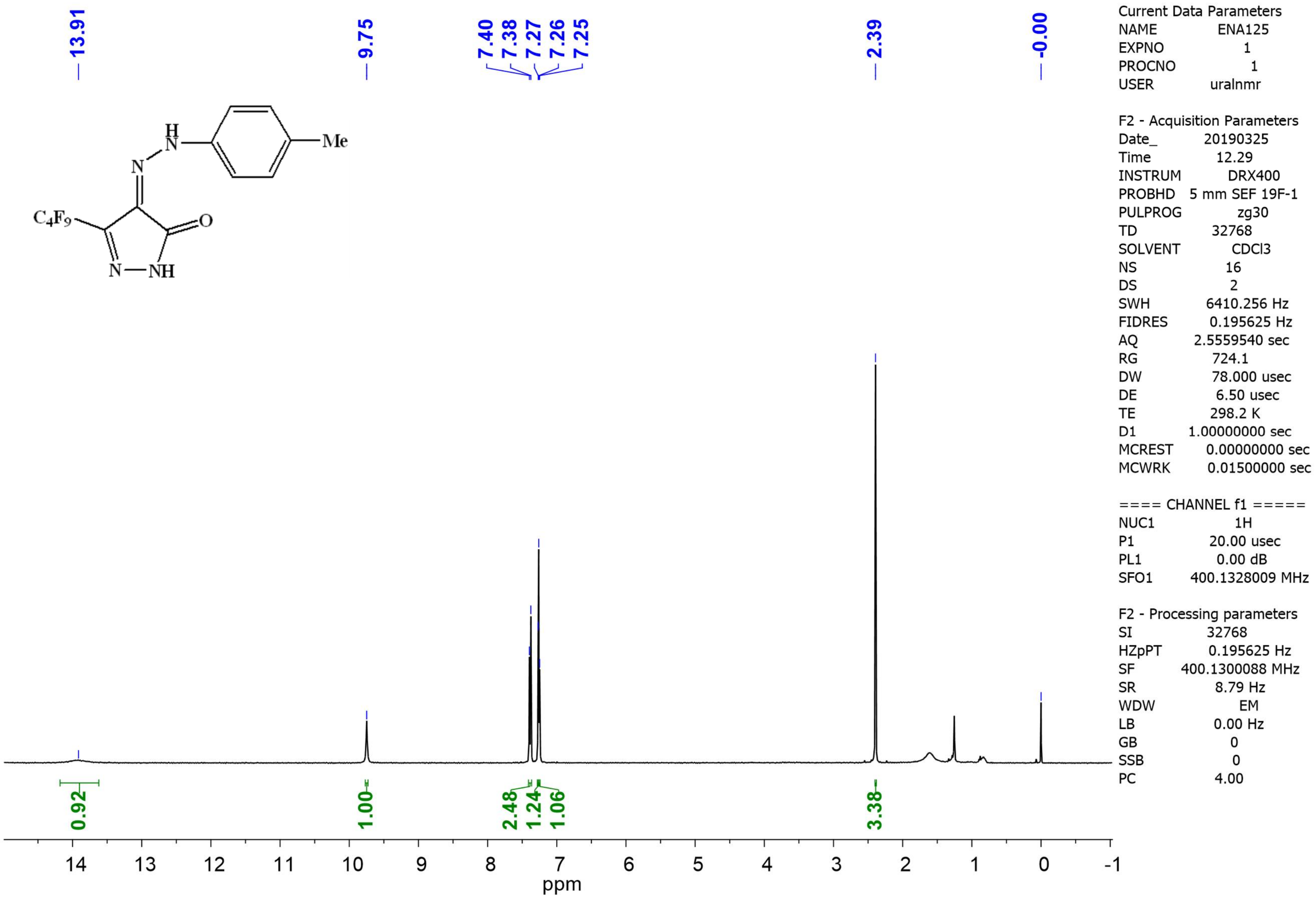

Figure S86. ^{13}C NMR spectrum of compound **8a**

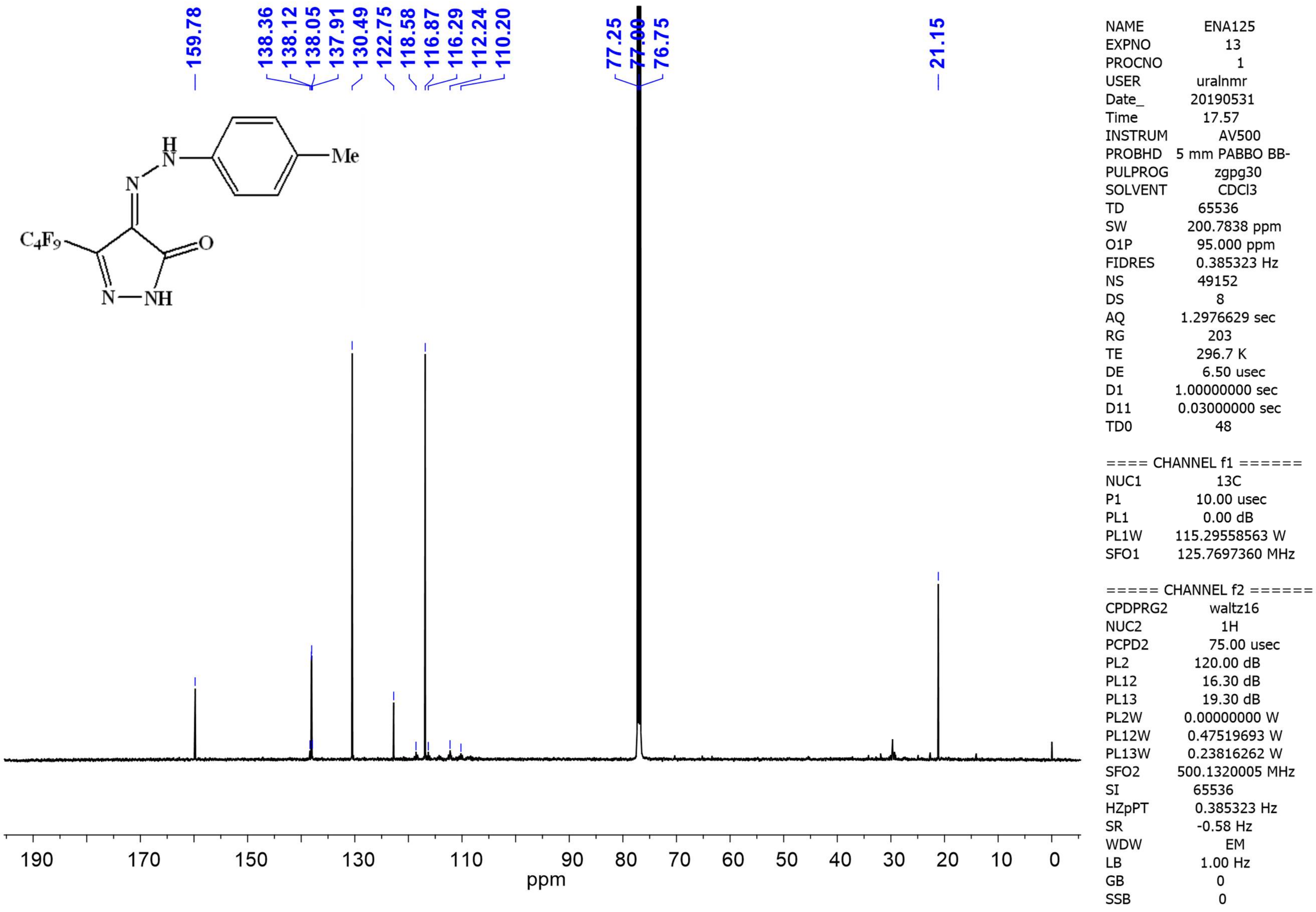

Figure S87. ^{19}F NMR spectrum of compound **8a**

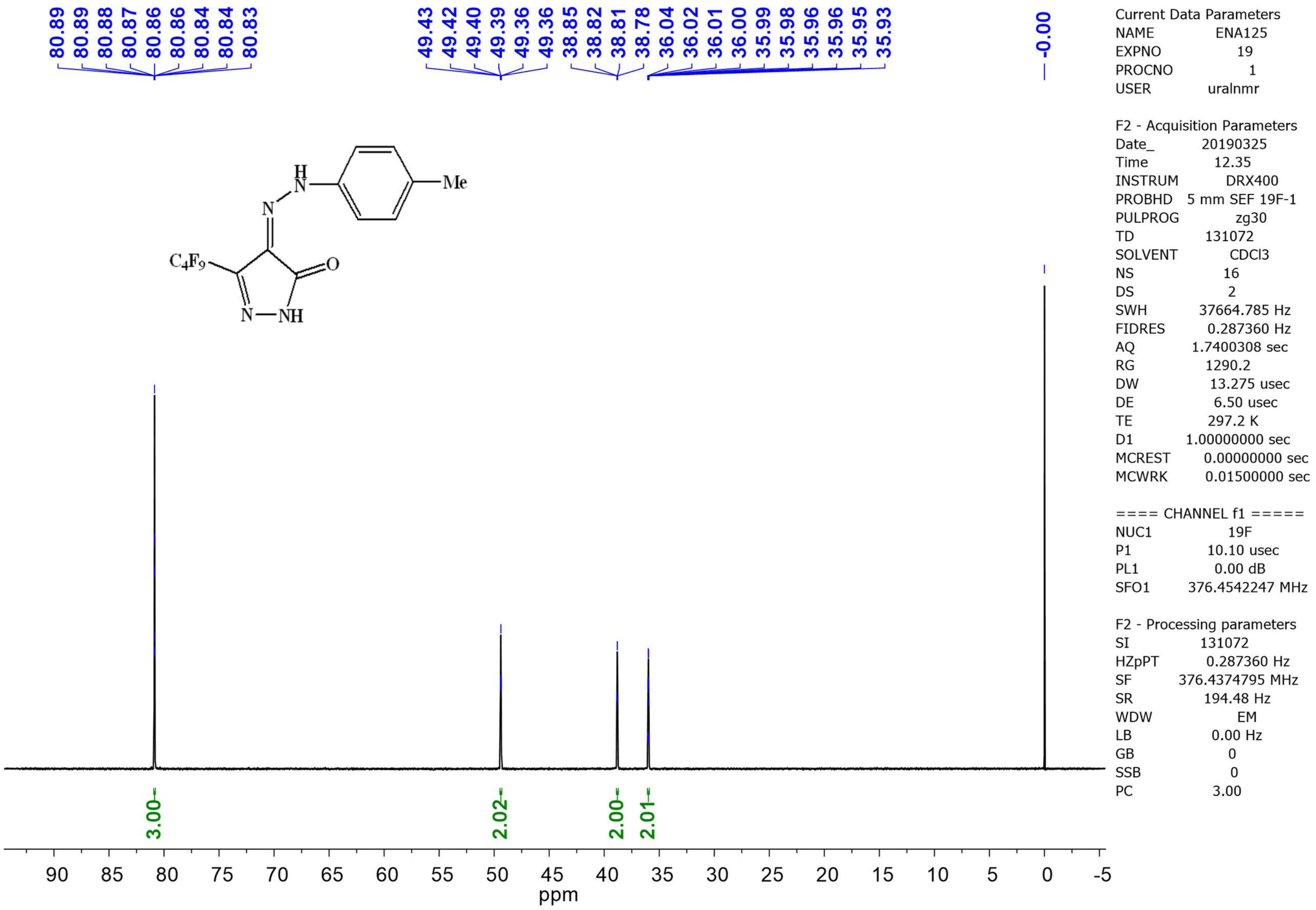

Figure S88. ^{1}H NMR spectrum of compound **8b**

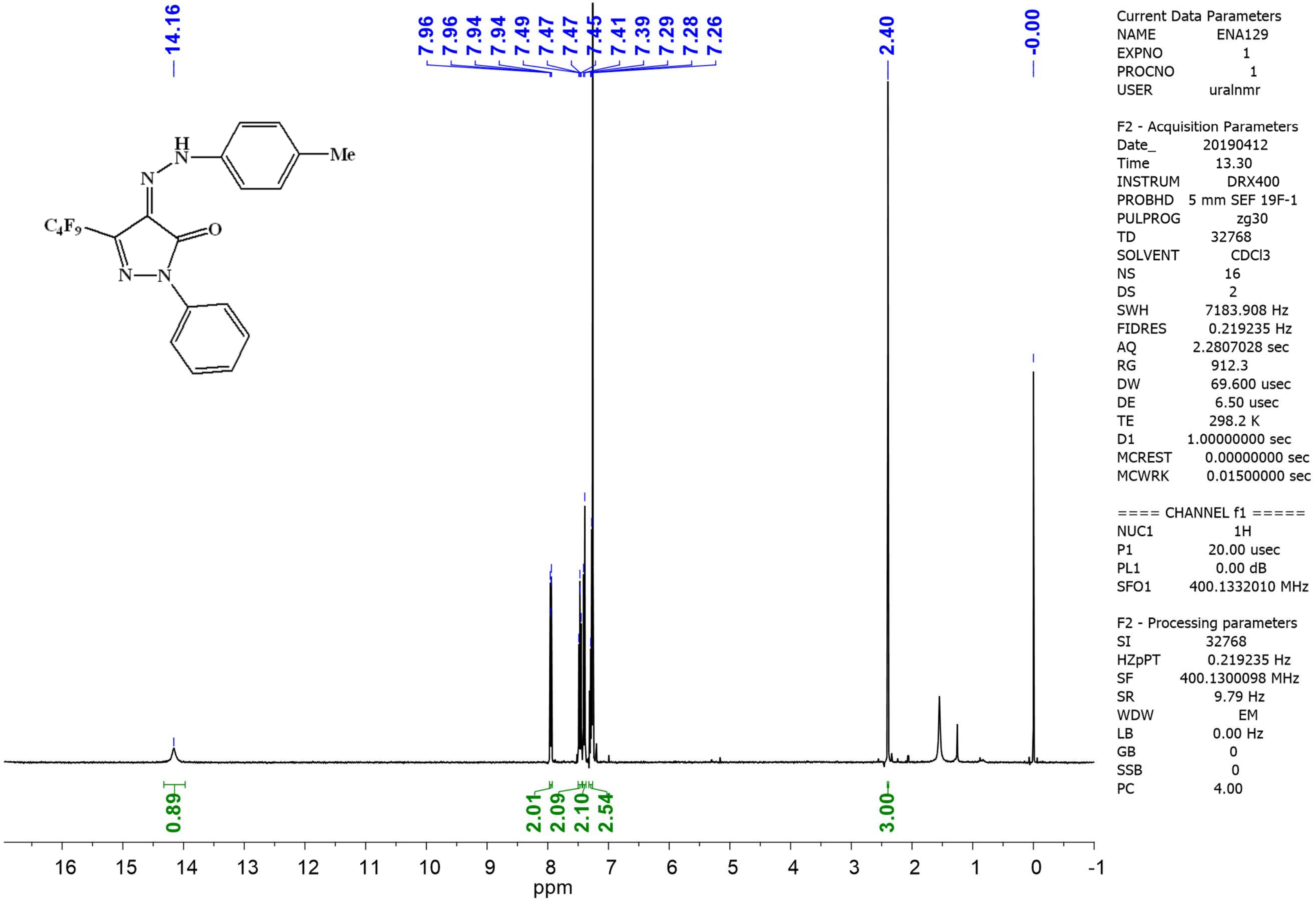

Figure S89. ^{13}C NMR spectrum of compound **8b**

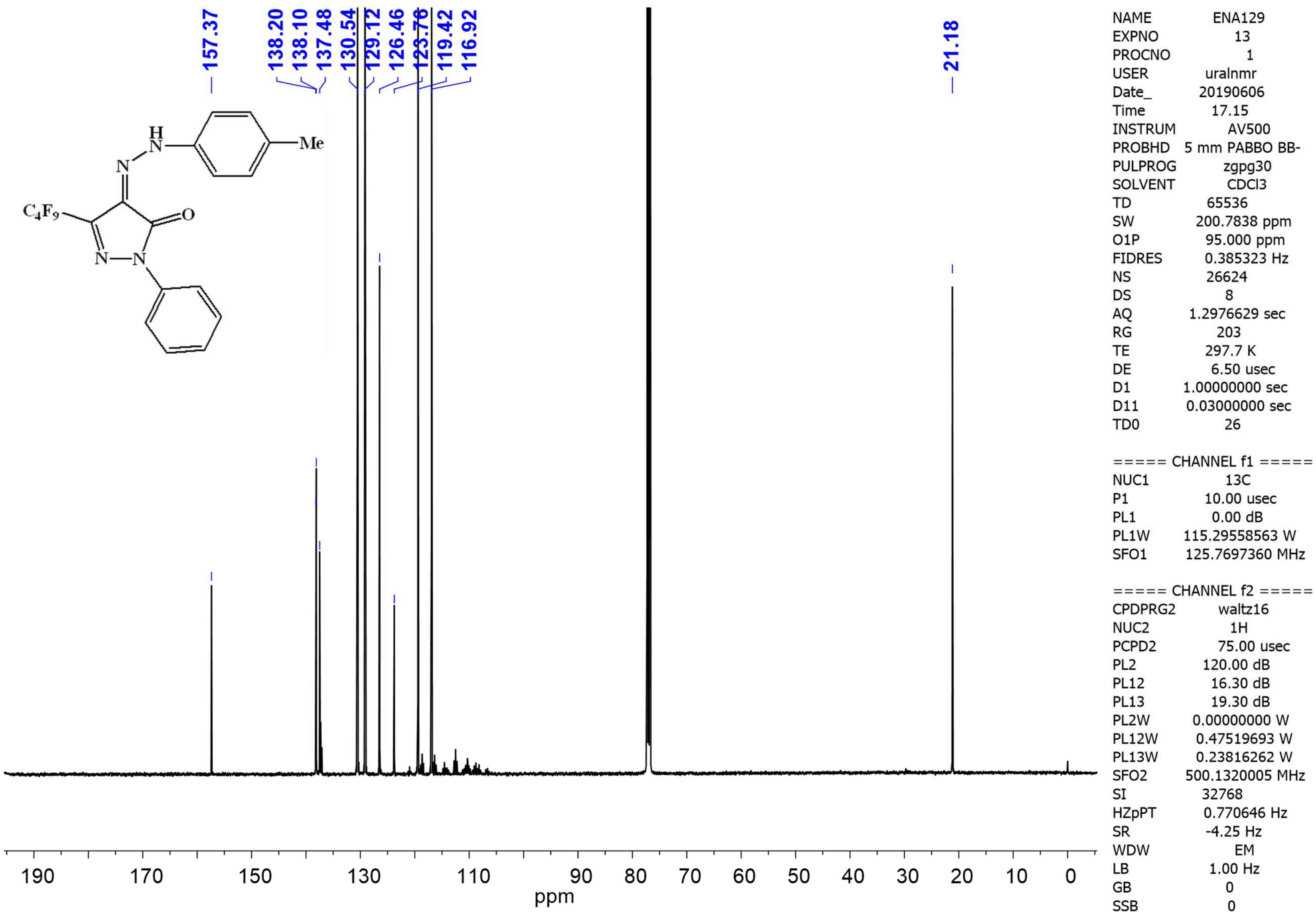

Figure S90. ^{19}F NMR spectrum of compound **8b**

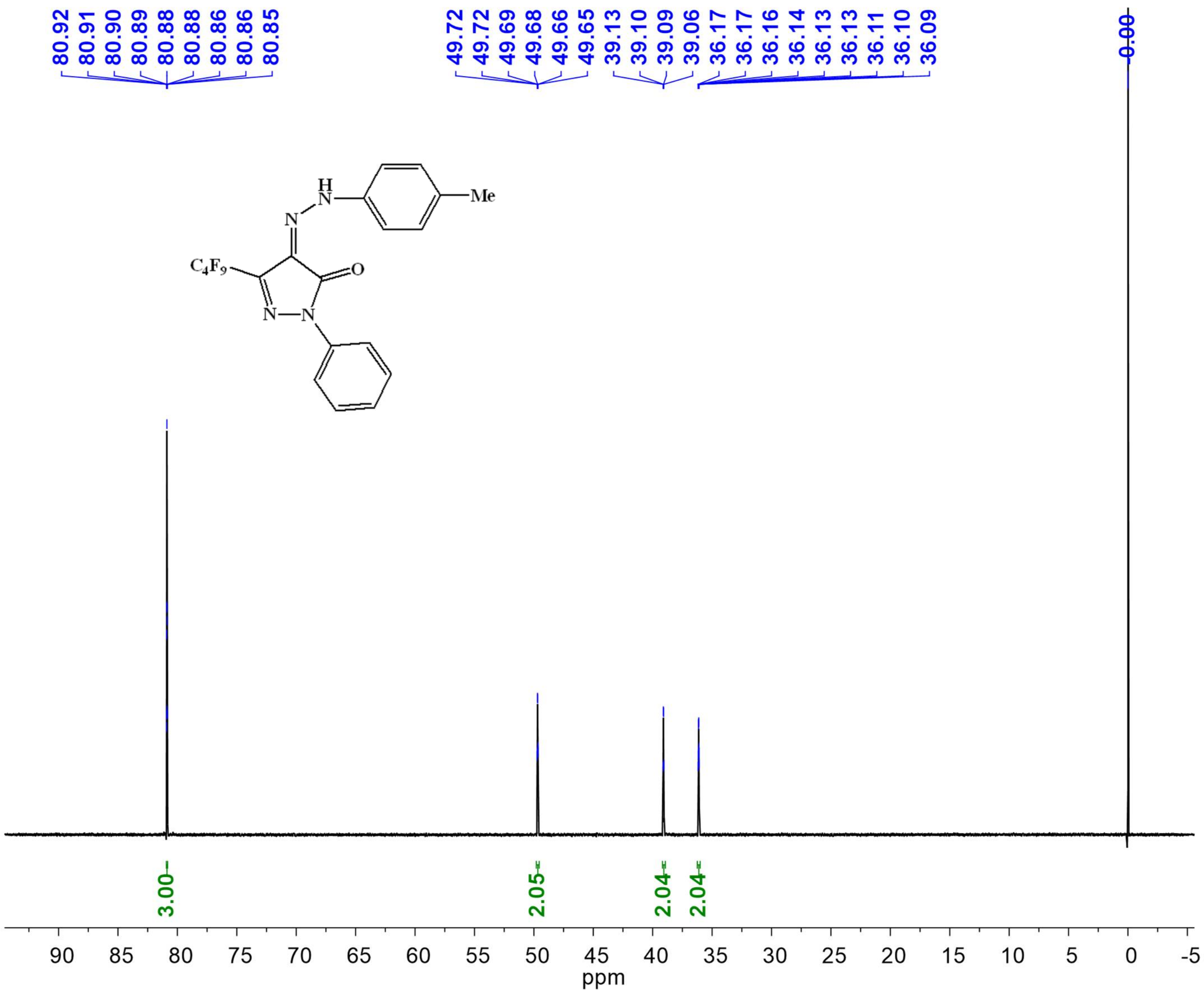

Current Data Parameters
NAME ENA129
EXPNO 19
PROCNO 1
USER uralnmr

F2 - Acquisition Parameters
Date_ 20190412
Time 13.34
INSTRUM DRX400
PROBHD 5 mm SEF 19F-1
PULPROG zg30
TD 131072
SOLVENT CDCl3
NS 16
DS 2
SWH 37664.785 Hz
FIDRES 0.287360 Hz
AQ 1.7400308 sec
RG 1824.6
DW 13.275 usec
DE 6.50 usec
TE 298.2 K
D1 1.00000000 sec
MCREST 0.00000000 sec
MCWRK 0.01500000 sec

===== CHANNEL f1 ======
NUC1 19F
P1 10.10 usec
PL1 0.00 dB
SFO1 376.4542247 MHz

F2 - Processing parameters
SI 131072
HZpPT 0.287360 Hz
SF 376.4374821 MHz
SR 197.08 Hz
WDW EM
LB 0.00 Hz
GB 0
SSB 0
PC 4.00

Figure S91. ^{1}H NMR spectrum of compound **9a**

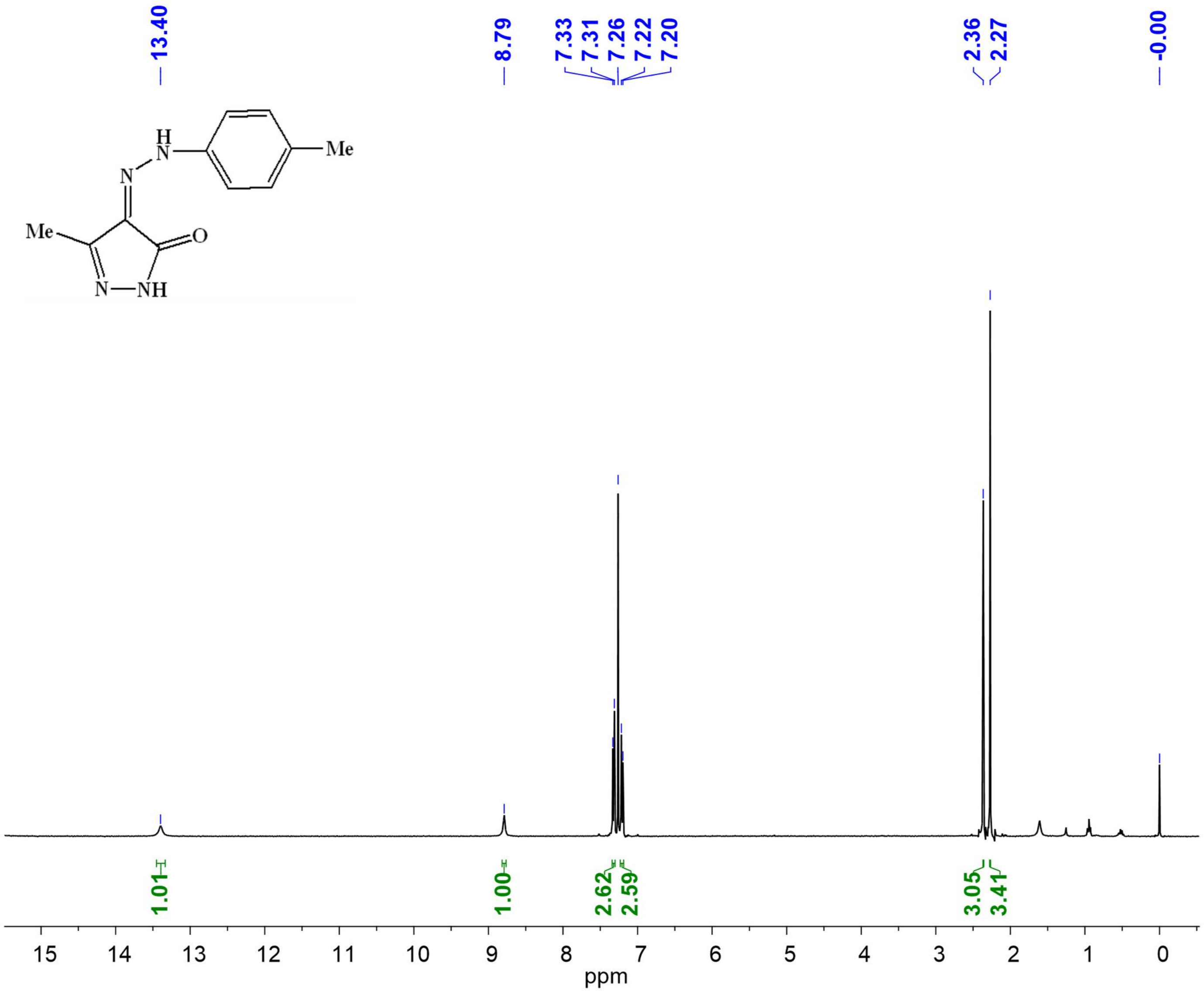

Current Data Parameters
NAME ENA124
EXPNO 1
PROCNO 1
USER uralnmr

F2 - Acquisition Parameters
Date_ 20190321
Time 13.12
INSTRUM DRX400
PROBHD 5 mm SEF 19F-1
PULPROG zg30
TD 32768
SOLVENT CDCl3
NS 16
DS 2
SWH 6410.256 Hz
FIDRES 0.195625 Hz
AQ 2.5559540 sec
RG 724.1
DW 78.000 usec
DE 6.50 usec
TE 298.2 K
D1 1.00000000 sec
MCREST 0.00000000 sec
MCWRK 0.01500000 sec

===== CHANNEL f1 ====
NUC1 1H
P1 20.00 usec
PL1 0.00 dB
SFO1 400.1330010 MHz

F2 - Processing parameters
SI 32768
HZpPT 0.195625 Hz
SF 400.1300091 MHz
SR 9.08 Hz
WDW EM
LB 0.00 Hz
GB 0
SSB 0
PC 4.00

Figure S92. ^{13}C NMR spectrum of compound **9a**

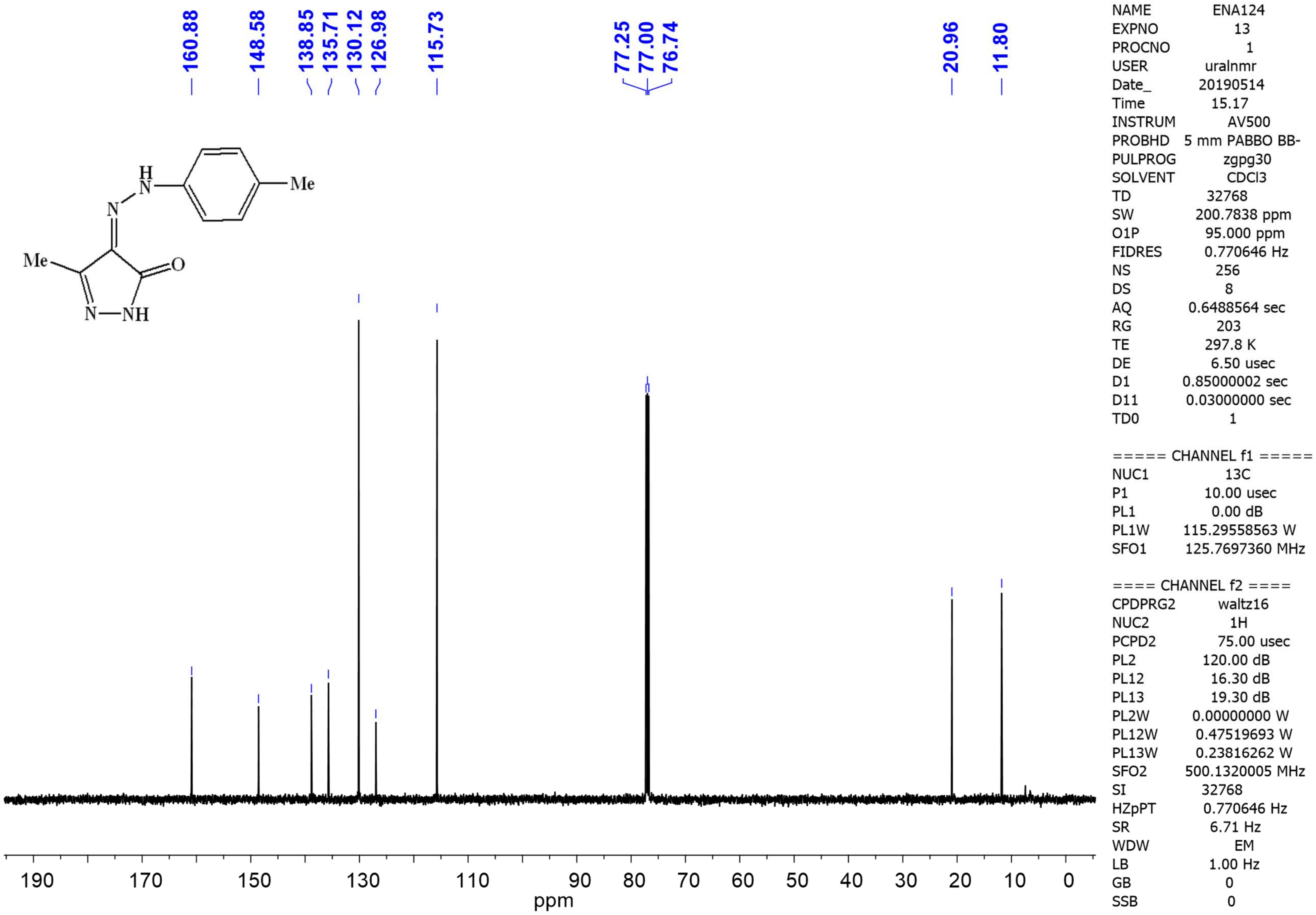

Figure S93. ^{1}H NMR spectrum of compound **9b**

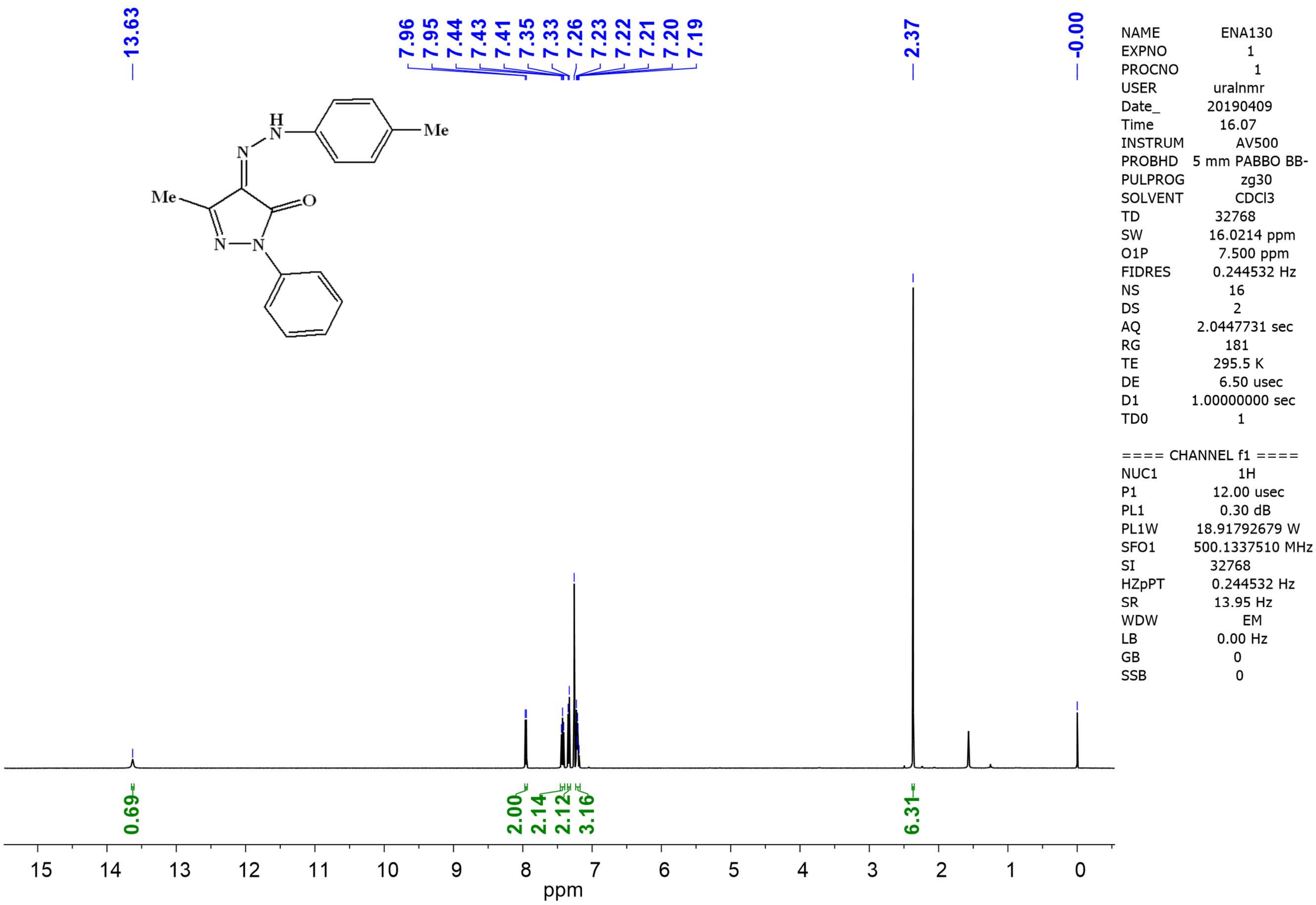

Figure S94. ^{13}C NMR spectrum of compound **9b**

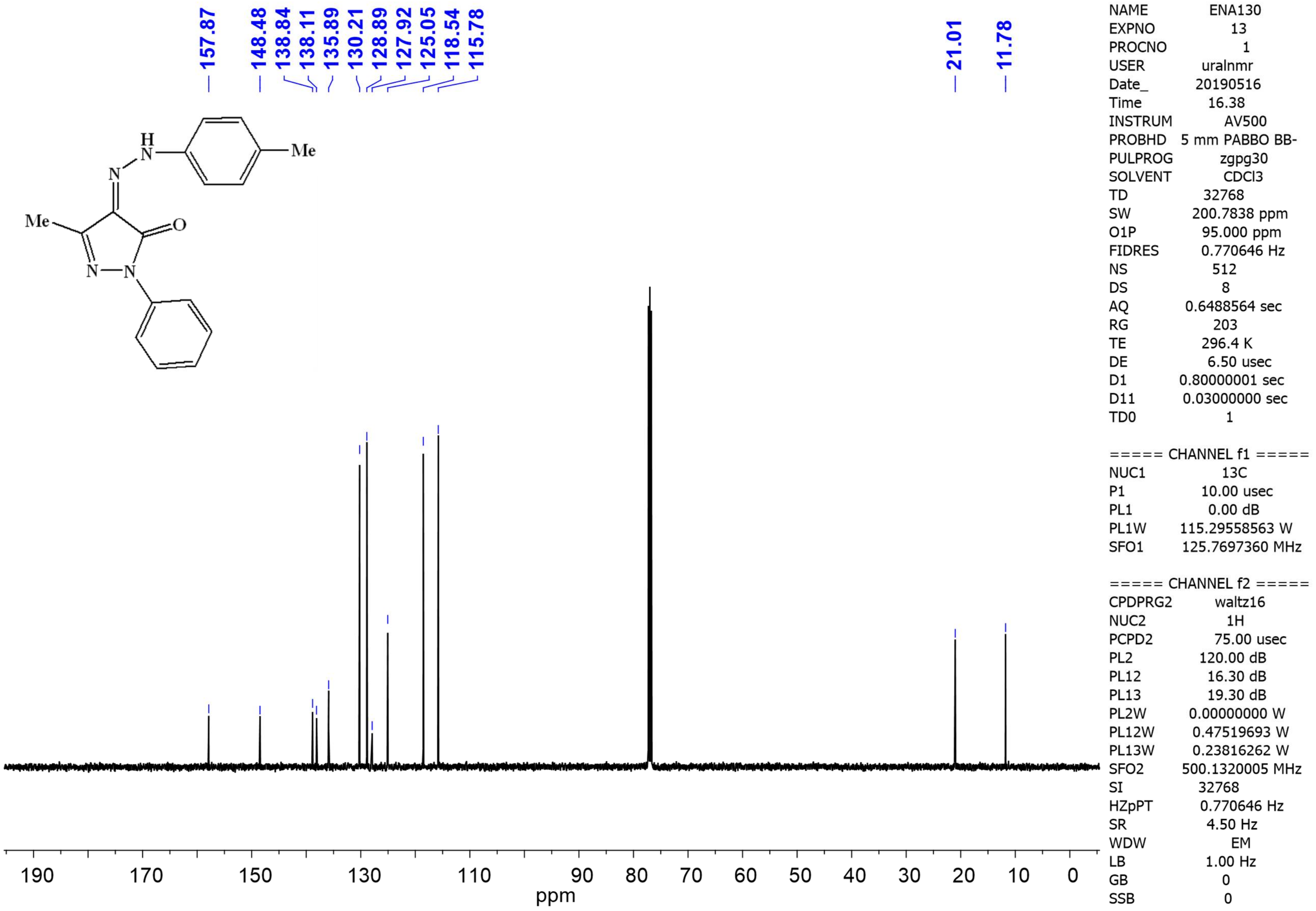

Supplement: Supplementary file 1 [file molecules-28-00059-s001.zip › molecules-2079579-supplementary.pdf]
